# Supplementary material for: The phylogenetic affinities of the bizarre Late Cretaceous Romanian theropod Balaur bondoc (Dinosauria, Maniraptora): dromaeosaurid or flightless bird?
Source: PeerJ. 2015 Jun 18;3:e1032. doi: 10.7717/peerj.1032 (PMC4476167; doi:10.7717/peerj.1032)
Supplement: Supplemental Information 1 — Details of phylogenetic analyses. Implied weighting analyses. Updated datasets. [file peerj-03-1032-s001.doc]

Electronic supplementary material for

# The phylogenetic affinities of the bizarre Late Cretaceous Romanian theropod *Balaur bondoc* (Dinosauria, Maniraptora): dromaeosaurid or flightless bird?

Andrea Cau, Tom Brougham, Darren Naish

This file contains:

1. Details of phylogenetic analyses
2. Results of implied weighting analyses
3. Modified dataset of Brusatte et al. (2014) - Data matrix
4. Modified dataset of Lee et al. (2014) - Data matrix
5. References for supplementary material

## Details of phylogenetic analyses

### Phylogenetic taxonomy

Our use of phylogenetic taxonomy follows Longrich and Currie (2009), Turner et al. (2012) and Godefroit et al. (2013). Accordingly, Paraves is the most inclusive clade containing crown birds but not *Oviraptor philoceratops*; Avialae is the most inclusive clade containing crown birds but not *Dromaeosaurus albertensis* and *Troodon formosus*; Dromaeosauridae is the most inclusive clade containing *Dromaeosaurus albertensis* but not crown birds and *Troodon formosus*; and Eudromaeosauria is the least inclusive clade containing *Deinonychus antirrhopus*, *Dromaeosaurus albertensis*, *Saurornitholestes langstoni* and *Velociraptor mongoliensis*.

**Tree search strategy**

Importing each dataset in TNT (Goloboff et al. 2008b), we performed 100 “New Technology” search replicates with paramaters set using default values (i.e., Sectorial Search set on, Tree Fusing set on; both with default parameters). The result of the first search was then explored performing Tree-Bisection-Reconnection (TBR) heuristic searches, and saving all shortest trees found. For each analysis, no more than 99999 trees were saved to reduce computational time. Nodal support was calculated performing 1000 TBR heuristic search replicates and saving all tree up to ten steps longer than the best score found.

Alternative placements and the Templeton's tests were performed in PAUP (Swofford 2002).

Three iterations of the Implied Weighting Analysis were performed with each data matrix in TNT, setting the concavity parameter *k* (Goloboff et al. 2008a) to 1 (strong downweighting of homoplastic characters), 3 (default value in TNT, Goloboff et al. 2008b), and 9 (moderate downweighting of homoplastic characters) respectively. Concavity parameters greater than 9 were also tested for both matrices, however, because the topologies of the resultant trees converged closely on the unweighted topologies, they were not considered in the final analyses.

### Brusatte et al. (2014) matrix

Turner et al. (2012) suggested that *Linheraptor* is a junior synonym of *Tsaagan*, an interpretation recently challenged by Xu et al. (2015). We agree with the latter authors that *Linheraptor* may eventually be a taxon distinct from *Tsaagan*, and that any Operational Taxonomic Unit (OTU) based on combined information from both *Tsaagan* and *Linheraptor* may represent a chimaera (if future analyses using the two taxa separately do not find them as sister taxa relative to other dromaeosaurids). Nevertheless, it is unclear whether some of the character scores of the *Tsaagan* OTUs present in both the Turner et al. (2012) and Brusatte et al. (2014) datasets include information based on *Linheraptor exquisitus* holotype. Although Turner et al. (2012) explicitly suggested the synonymy between these two dromaeosaurids, we note that the *Tsaagan* OTU in both the Turner et al. (2012) and Brusatte et al. (2014) datasets seem as scored solely on the *Tsaagan mangas* holotype (a specimen which includes a complete skull as well as partial postcranial bones): after checking the scores of the *Tsaagan* OTU in the Brusatte et al. (2014) dataset, we were unable to find any character state that could be unequivocally based on the well complete holotype skeleton of *Linheraptor exquisitus* instead of *Tsaagan mangas* type specimen. As a single example, although character “118.1” of Brusatte et al. (2015: describing the elongation of the distal caudal prezygapophyses – a well-established synapomorphy of non-unenlagiine dromaeosaurids, Turner et al. 2012) is clearly evident in the *Linheraptor* type specimen based on the published literature (Xu et al. 2010), the *Tsaagan* OTU in that dataset is cored as “118.?”, which is the state expected if *T. mangas* is scored using the only holotype of the latter species (a specimen missing the tail). Therefore, we conclude that the *Tsaagan* OTU in the Turner et al. (2012) and Brusatte et al. (2014) datasets were probably scored exclusively on the type specimen of *Tsaagan*, with no scores from *Linheraptor*. This suggests that the *Tsaagan* OTU in both datasets is not a potential chimaera, regardless of the taxonomic interpretation of *Linheraptor* discussed by Xu et al. (2015).

The following characters were modified from their original definitions. Comments regarding the nature of the modifications are included in brackets.

Char. 145: Semilunate carpal, placement relative to metacarpals: medially placed, mostly overlapping metacarpals I and II, marginally or not overlapping metacarpal III (0); laterally shifted, marginally or not overlapping metacarpal I, significantly overlapping metacarpal III (1). [Previously, the character included four states that partially overlapped, describing size and placement of the semilunate carpal. Here, the character is simplified to describe exclusively the relative placement of the semilunate carpal instead of the combination of size and position].

Char. 147: Third manual digit, number of phalanges: (0) four; (1) three; (2) two; (3) one; or (4) splint metacarpal bearing no phalanges. The character is ordered. [States redefined to better describe variation among theropods].

Char. 436: Metatarsal V length: (0) less; or (1) more than 40% of metatarsal III’s length (Brusatte et al. 2013). [Previously, the character did not quantitatively define metatarsal V’s elongation]. The derived state is present exclusively in microraptorine and eudromaeosaurian dromaeosaurids among Coelurosauria. *Balaur* shows the plesiomorphic state.

The following new characters were included:

Char. 854: Metacarpal I, proximal half: mediolaterally expanded, width comparable to rest of bone (0); narrower than distal half, medial margin sloping proximolaterally (1).

Char. 855: Distally closed intermetacarpal space between metacarpals II and III: (0) absent, metacarpals not contacting distally; (1) present. This character is not redundant with character 391, which describes the extent of the intermetacarpal space among the taxa bearing the closed intermetacarpal space and is scored as ‘inapplicable’ among the other taxa.

Char. 856: Metacarpal III, distal end: (0) bicondylar; or (1) simple convexity.

Char. 857: Dorsal margin of manual unguals: (0) does not; or (1) does arch dorsally above level of articular facet (Senter 2007; Agnolín and Novas 2013).

Char. 858: Interpubic space, width between conjoined pubes: (0) gradually narrowing distally; or (1) wide pubic canal and laterally bowed pubis, followed by an abrupt narrowing at the symphysis.

Char. 859: Length of pedal phalanx I-1: (0) < 66% III-1; or (1) > 66% III-1. The derived state is present exclusively in a subset of avialans among Coelurosauria. *Balaur* shows the apomorphic state.

Char. 860: Metatarsal II, distal condyles, plantar projection: (0) medial and lateral condyles with comparable projection; (1) medial condyle much further projected ventrally than lateral. (O’Connor et al. 2014). This character describes the marked medial projection of the distal condyles of metatarsal II present in *Balaur* and some avialans.


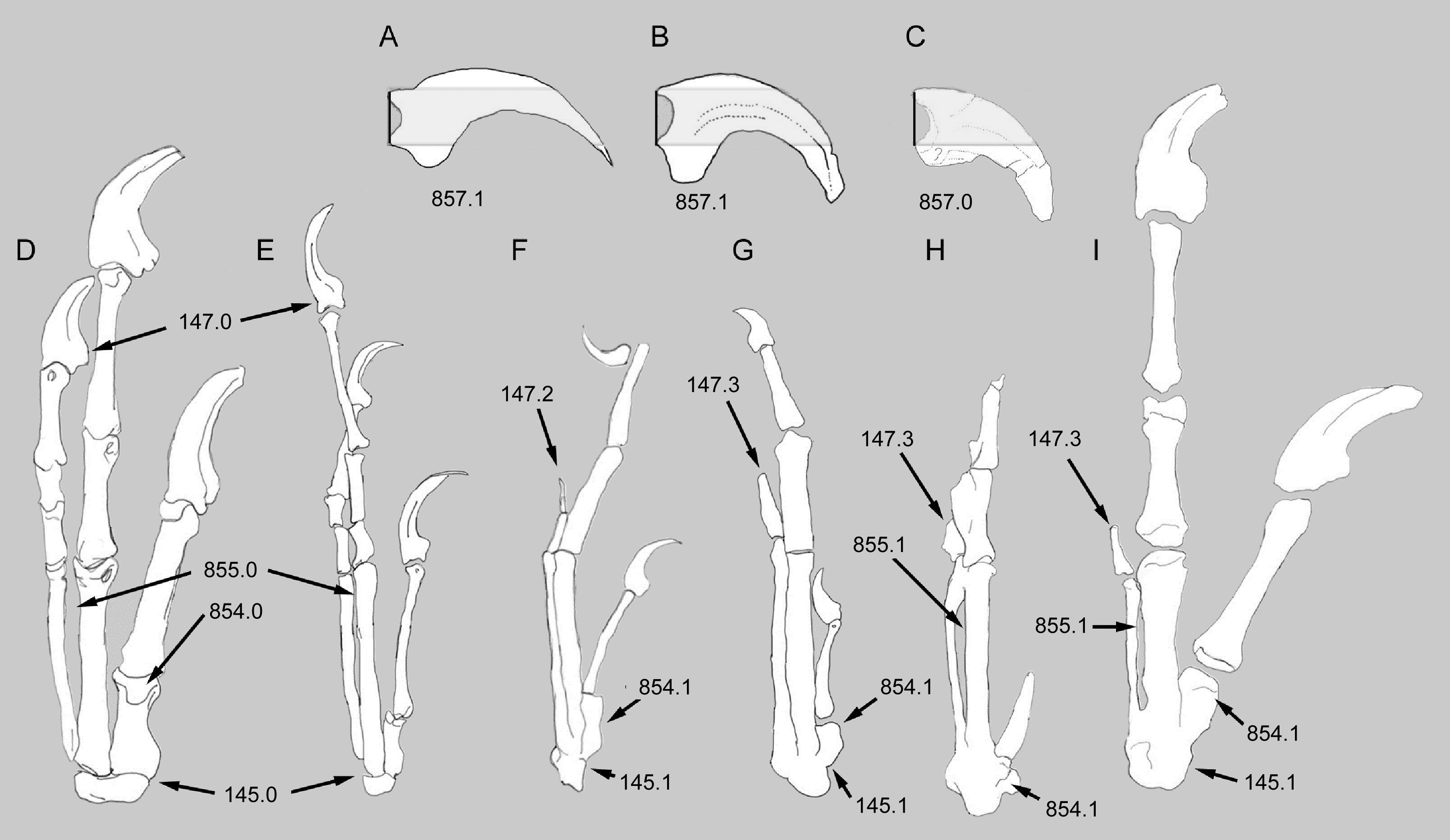


Figure S1. Manual ungual I of *Microraptor* (A), *Velociraptor* (B) and *Balaur* (C), in side view, all drawn at the same proximal facet depth, with articular facet dorsoventral axis oriented vertically. Left hand of *Deinonychus* (D), *Archaeopteryx* (E), *Sapeornis* (F), *Zhouornis* (G), *Nothura* (H), and *Balaur* (I), in extensor view, all drawn at the same metacarpal II length. Modified from (A), Senter (2007); (B), Norell and Makovicky (1999); (C, I), Brusatte et al. (2013); (D, E, H), Wagner and Gauthier (1999); (F), Zhou and Zhang (2003); (G), Zhang et al. (2013). Numbers refer to character states in the character list modified from Turner et al. (2012).


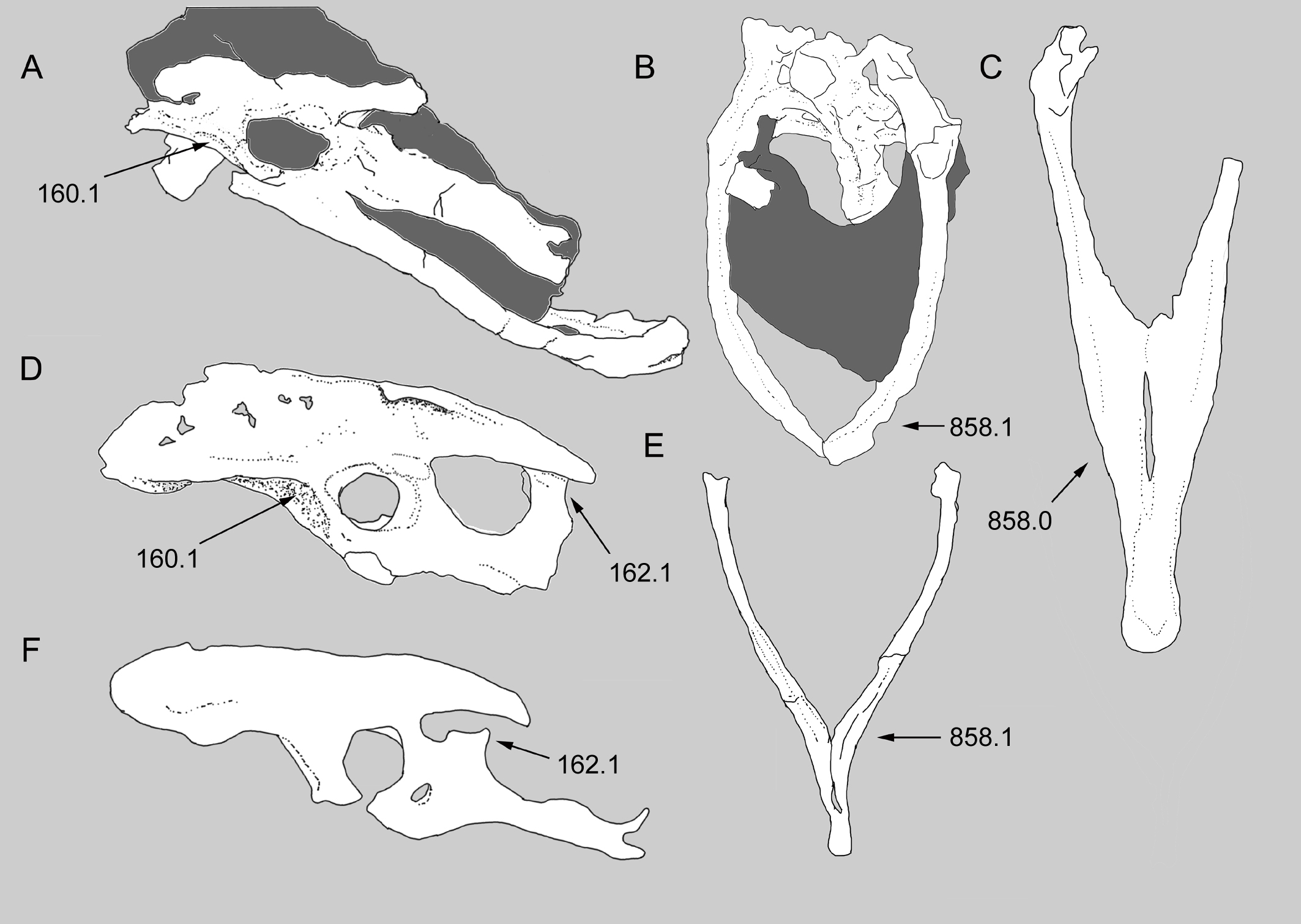


Figure S2. Pelvis of *Balaur*, in lateral (A) and anteroventral (B) views. Pubis of *Velociraptor*, in posterior view (C). Pelvis of an unnamed enantiornithine from the Maastrichtian of Argentina, in lateral view (D). Pubis of *Sapeornis*, in anterior view (E). Pelvis of *Archaeopteryx* (London Specimen), in lateral view (F). Modified from (A, B), Brusatte et al. (2013); (C), Norell and Makovicky (1999); (D), Walker and Dyke (2009); (E), Zhou and Zhang (2003). Numbers refer to character states in the character list modified from Turner et al. (2012).


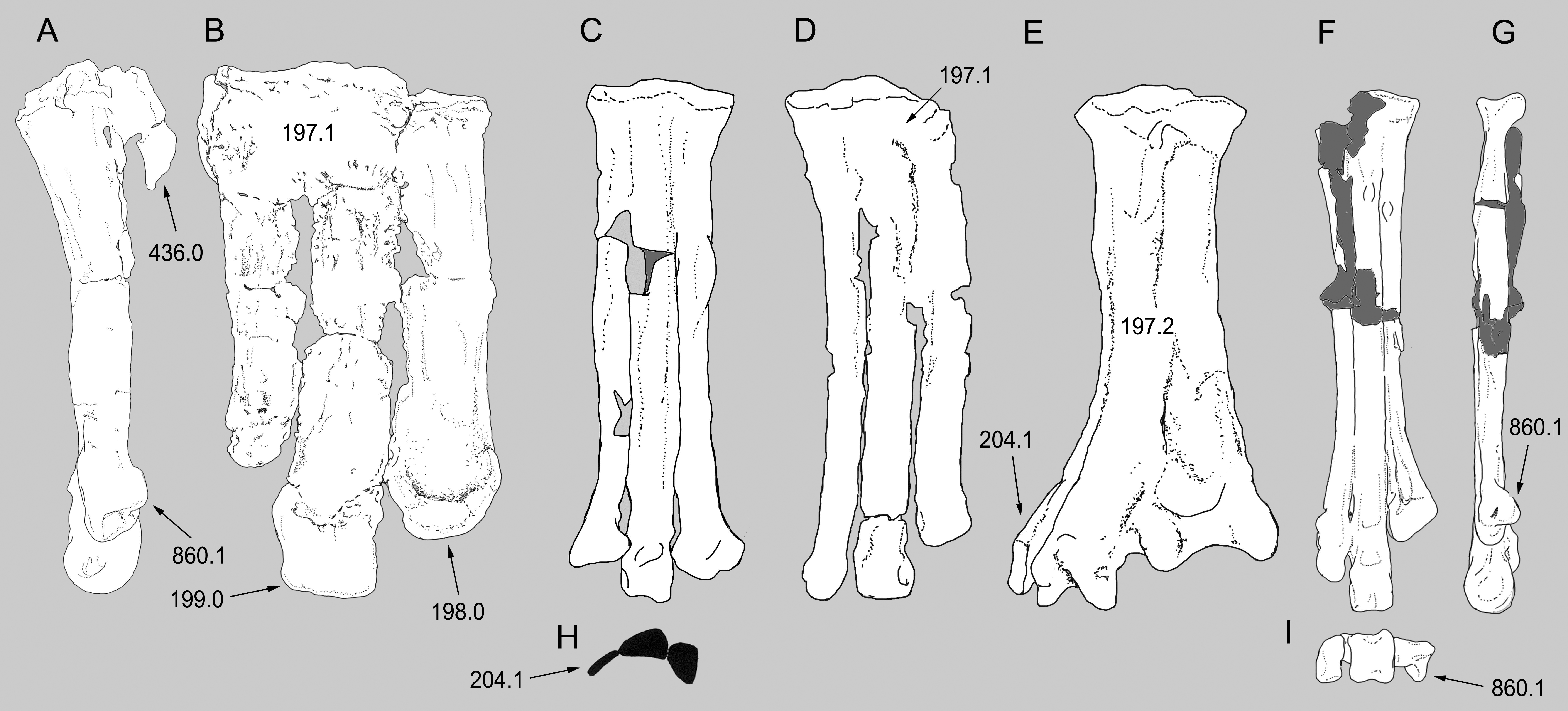


Figure S3. Tarsometatarsus of *Balaur*, in medial view (A). Tarsometatarsi of *Balaur*, *Avisaurus* *archibaldi*, *Bauxitornis*, *Yungavolucris* and *Evgenavis*, in extensor view (B-F). Tarsometatarsus of *Evgenavis*, in medial view (G). All drawn at the same metatarsal III length. Mid-shaft cross section of tarsometatarsus of *Avisaurus* *archibaldi* (H). Tarsometatarsus of *Evgenavis*, in distal view (I). Modified from (A, B), Brusatte et al. (2013); (C, H), Brett-Surman and Paul 1985; (D), based on photograph provided by A. Osi; (E), Chiappe (1993); (F, G, I), O'Connor et al. (2014). Numbers refer to character states in the character list modified from Turner et al. (2012).


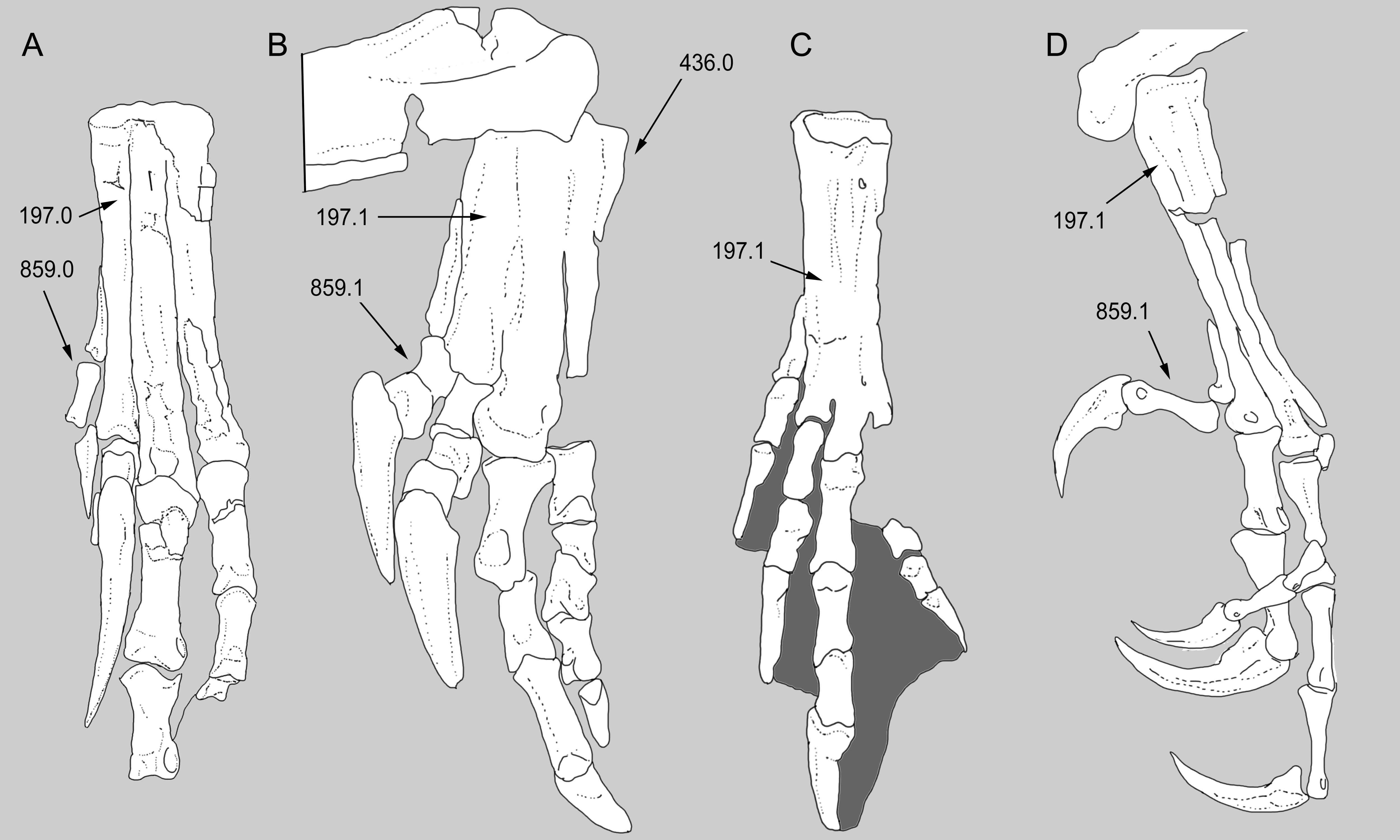


Figure S4. Feet of *Velociraptor* (A), *Balaur* (B), *Patagopteryx* (C) and *Zhouornis* (D), in extensor (A, D), lateroextensor (B), and flexor (C) views. All drawn at the same metatarsal III length. Modified from (A), Norell and Makovicky (1997); (B), Brusatte et al. (2013); (C), Chiappe (2002); (D), Zhang et al. (2013). Numbers refer to character states in the character list modified from Turner et al. (2012).

### Lee et al. (2014) matrix

Modifications involved re-definition of character 318 to avoid ambiguity in its interpretation:

Metacarpal III, distal end, medial contact with metacarpal II that is proximodistally extended (metacarpals II-III eventually enclosing an intermetacarpal space): absent (0); present (1).

## Results of implied weighting analyses

*Brusatte et al. (2014) modified dataset*

Test 1. *K*=1. Best score= 508.30979. Number of shortest trees= 7965.

Test 2. *K*=3. Best score= 334.02109. Number of shortest trees= 19800.

Test 3. *K*=9. Best score= 173.17934. Number of shortest trees= 10800.


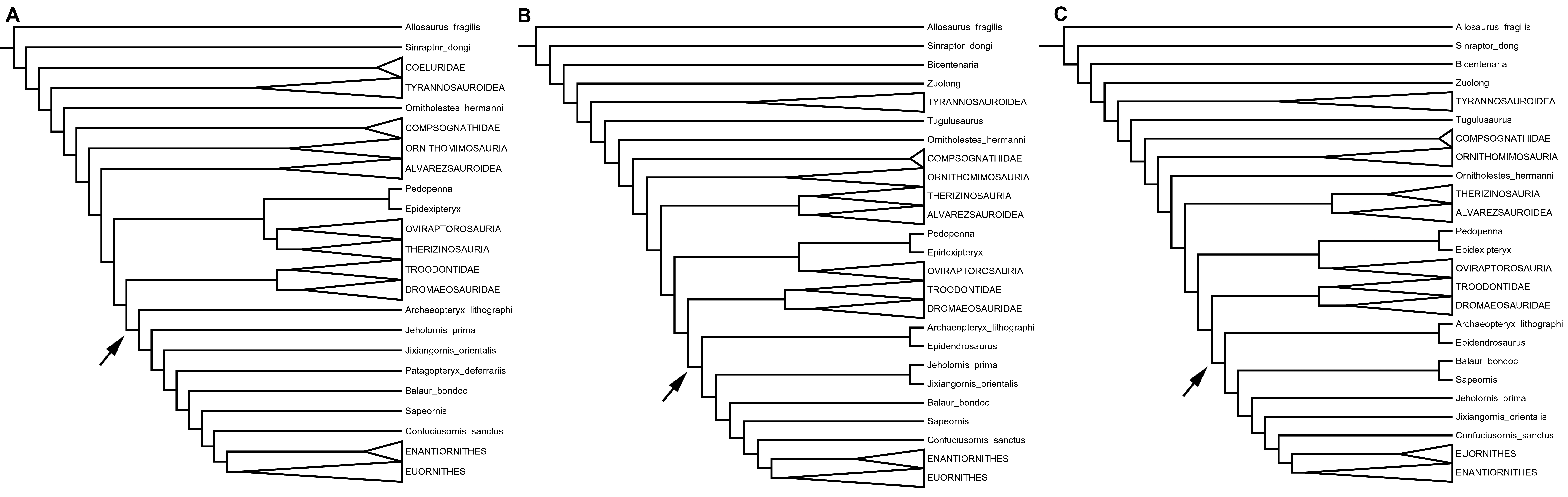


Figure S5. Strict consensus trees of the implied weighted analyses of the data set modified from Brusatte et al. (2014), with *k* parameter set as (a) 1, (b) 3 and (c) 9 respectively. Arrow indicates Avialae. Main clades collapsed for brevity.

*Lee et al. (2014) modified dataset*

Test 1. *K*=1. Best score= 858.75766. Number of shortest trees= 2.

Test 2. *K*=3. Best score= 592.85932. Number of shortest trees= 2.

Test 3. *K*=9. Best score= 323.86912. Number of shortest trees= 1.


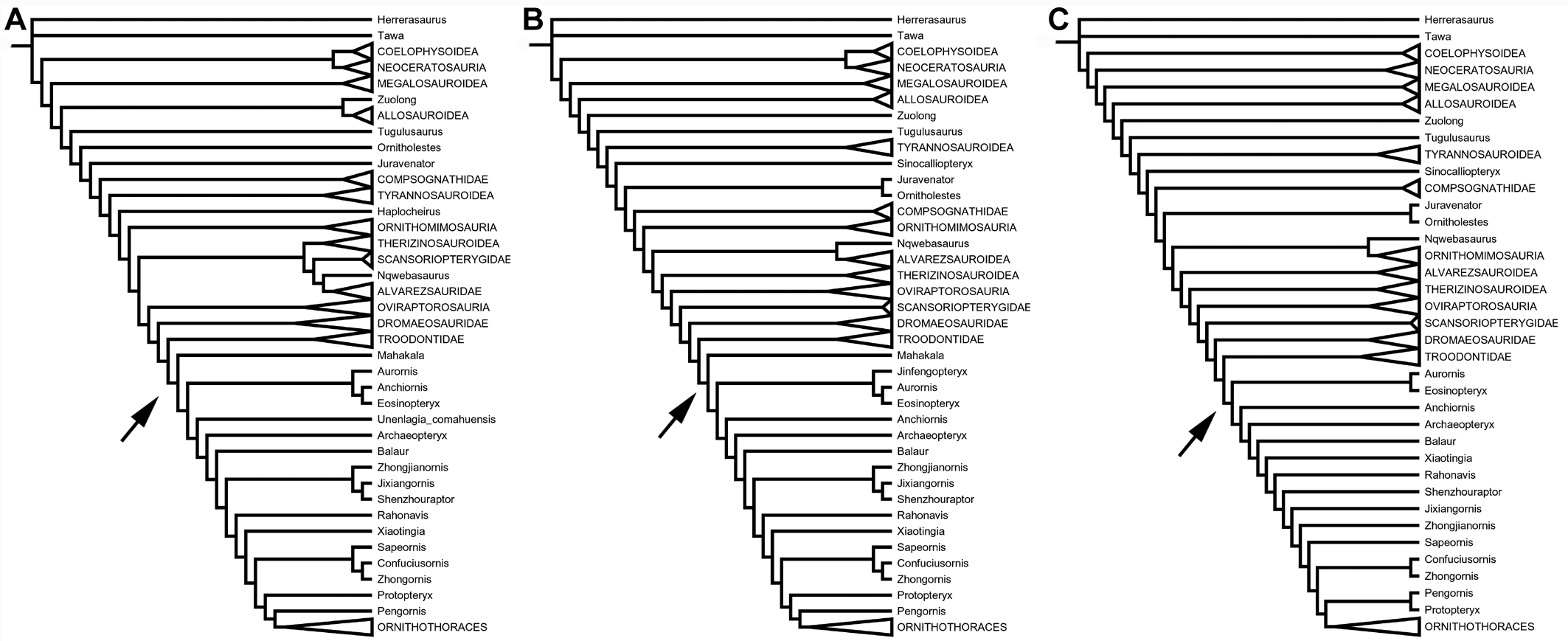


Figure S6. Strict consensus trees of the implied weighted analyses of the data set modified from Lee et al. (2014), with *k* parameter set as (a) 1, (b) 3 and (c) 9 respectively. Arrow indicates Avialae. Main clades collapsed for brevity.

**3 Modified dataset of Brusatte et al. (2014) – Data matrix**

xread

860 152

Allosaurus_fragilis

?10000?00000001000110010001012001110110010??000000000000100000000001000100000000000101010010000000100101000000000001000000000????000000000000000?1000000000001000010010100000010001000001100000010000000000000000000100000000000?0?000?00110000100000010000111000210110000000002000000000?0000??0000000?00000??0000000010000[01]0?0????????0000020?00?0000000101000?0?0010000?00000000000001000010?01000??0000000?000?000002000000000000?00000?00?0010000000020000?000100??001100000[01]0?00000000100000000110000000000100011000000021121001001000100?1????000000000100??10?0000?00000000000011000?100?000?0?1000000000????000?00010010000101000000000000000102000000000000000000010000000000020101[01]00001000000000000000000100000010?00010000010201100000000011000000001000000000000001001000100000000000000000000000000000000000000000000000??000000000000000000000?000001000000000000000?0000000

Sinraptor_dongi

?10000?000?0001000100000001012000010110010?00?000000000010000?00000100?10?000000000101010010000000100101000000000001??????0??1?10?0?0??00??0??????000000000?010??01001010000001?0?1000001100000010000000000000?000?000?00?000000?0?000?001100001001000100000100002101???00000000000000000?0000??0000000?00000??0000000010000???00?00??00?????2????????????1010???????????????????????????????????1??????0??000??000000002000000000000000000?00?00??0???00020000?0001?0??0011000??????????00010000000000000000001000001100000002102100000?010?00?10000000000000100??01?0000?00000000000?11000?100?010?0?100?00?001000?0010000100100001010?0?00000000000112000000?00????00000010000?00000020101??0??1000000????????????000010010?0000?000010201101010?00011000000??1?00000000000001001000??0???000000000100?00000000000000???????00000000??000000000000000000000000?0010?00000?00000001????0?0

Dromaeosaurus_albertensis

?0??001000000000010??0???0?01??01110????1111????101?1001100??00000010011111000?0000101001?????????????????????????????????????????????????????????????????1??????????????????????????????????????????11?1?0????00??????00?0?00??1?????????1?00??011000100?0???????????????0???0000?00000??0000??0000000?00000??00?????????????????????????????????????????????????????????????????????????????????00??????????????????????????????????????0????0?????????010121?000??0???00?0?????????0????0??0??0???0??????0?0100?00?00????????????????????????0????00000100000???????0???????0001001?0?0????00??00?????0?0?10???????????000?1000000010????00000000001120100000000?0?00010??????????????????????????????????????????????????????????????????????????0??000?0?????????????0??0?010????????????????????????0200000????????????????????????????????????0?1?0??110????????????????000?0????????

Deinonychus_antirrhopus

?0010????1???????1??0000?0111000111000111111100?1??00001110100000?0100111?1000?0100101000?110001100?1121011????00110111121??????1?1101110010011100000100221110101020220?01111211201?1111000000000100011010010000000001100000?00010?0010002110000011110110000??00000122000000000?0????000????0???????????00000??000000000000?[01]0?0?????????????20000?000?000111000?0?0010000?0000000000000101?0???00000??0100000?000?000000000000000000000000000?00101000000????????0?1????00111?????????0000?????00001?10?0010001?000001010??00?0???????00??0?0??0?????00001000000??01?00?0?00000001??????????????????????0?0?1001000?000?00?????????????????00000000001120100?0000?0?????00000000?00001000100?????00000?1101010000100??1?0000?????????????????00000?000000000?00?100?00??0001?1??0?000010?100000000010?010??00?000000000000000000000000??0????000000000100??1101?000110???200?10001?00001?00

Velociraptor_mongoliensis

?001001001000012011200001011100011100012111?10?0101000011101000000010011111000101001010000110001100111210111110001101111211110101111111100100111000001002211101110202202011112212011111?000000000111011010010000000001100000000011?00101021100000111001100000000000112000000000000?000000?0000??0000000??0000??0000?00000000[01]0?00??000?00000020000?000?000111000?0?001000???00000000000010100???00000??0100000?0000000000000000000000000000000?0110100000010[12]21?0001101100011100000?100000000?0100001?10?0010001?0?00010101000000???0??0?01??00?0????000001000000??01?0000?00000001001?0?0???000??00110100?0???01000?000?000001001000?00000000000??00?112010000???????00000??????????????01001000?000001110101?0?0?00?0100000110?01?0100000000000000000000000?000100000?000?101010?0000100100000000010?01002000000000000000000000000000??0000100000000010000?1?10?0011010020001000100000100?

Balaur_bondoc

????????????????????????????????????????????????????????????????????????????????????????????????????1?2??1?2??0???10?????????0????111[01]11101001?1103001???2???0111?202?02?0?1?22122????????????????2110001001000??00??1???????????0?00??0??????????????????????1?????2?00???????????????????????????????????????????00000000?[01]???0????????????20??????0???0111000?0?001000???010?00?????010?00???21??0?00000020?100??0000??????00???1?000000000?00??0?00000????????010????????1????????00000??????????????????????????????????????????????????????????????????????????????????????????????????????????????????????????????????????????????????????????????????????????????????????????????0100?0?0??0?00?1101010000?00?01000?0110?01?????????????000?00??00?????10100000?00??1??????0000100100?0000?????0?0???????????000000000000000000??000????000000210????????000?10???200?1??????1110111

Tsaagan_mangas

?00100100100001201120000101010001?1000121111100010110000100??000000100?11110001010010100001100??1??????0?????????????????1????????11?111???????????????????????????????????????????????????????????????????????00??00??00?0000???????????2110000011?00?0000000?00001????0000000????000000?0000??0000000?00000???000??????????????????????????20000???0?0?0??10????????????????????????????????????????????????????????????????????????????????????0????????0121?00???0??00011?0????????????00?0100001?10?0010001?0?00010101000?00???0??0?01??00?0????000001000000??0??0000?00000001001?000???000??001101?0?0???????????????0001001000000000000000??00?112010000000????000000???00?00001??????????????00?110???????????????????????????????????????????????000?000?????????0?10?010?????10?????????????????02?0000000?????????????????????????????????????000?1?1????110????0???000?0????????

Bambiraptor_feinbergorum

?0010??001000012010[02]00?010111000?1100012111?1000101?00011?0?0?000?0100?1111000?010010100??1110?1100?1??100?0?1100[01]1011??2????0101011011110?001?100000111?211?0101?202202?111022100???11100000000010001101?000000000001100000000011?0010?021100??11?110??0000?000000122000?00000?0??00000??0000??0000000?1?000??000000000100??0??0?0?0?0?0000020000?00000?011?000?0?0?1000??000000000?00010??????00000??0000000?000?000000000000000000000000?00?011?100000000121?000110??00011101100?10000000010100001?10?0110001?0000010001000000???0??0??1??00?0????000001000000??01?0000?00000001??1?0?????000??001101???????????????????0????????????????0?000??00?1120100?????????????0000000?000010001011000?000001110101000010?00100000110?01?010000000?00000?000000000?0001?000??00001?10?0?100010?100????????????????0?000?????00??00????000000??00001000000000100?0???10000110100200?10001????01?0?

Tianyuraptor_ostromi

??01?????????????????0????111?????????????0??1???????????????????????????????0?0?0?1010??0???0?????????0?0?0?1??01?0112???????1??00?011110?001?1000001112?1??0?1102223?21111022?0???1?110??????????00?11????000??0???1??00?0??01???1011?0???0??????1?0??????????0????2?000?0??????????????????????????????????????0??????????0?00?00????00000?0?00?000?0?0??????????????????????????????????????????????????00?1?0????10??????????????0?0????????20?101????????????1???????????0000?10??000??1???????????????????????????0??????????????????????????????????????????????????????????????????????????????????????????????????????????????????0?????????????????????????????0???????????????????????????01?1?1???00??0?0????000???????????????0?????0?0???00????0????0?0???0???????0???0?????0??0??????????????????0?????0???0?????????????????????????0010????????0?0???????????0?0??????????

Sinornithosaurus_millenii

0001??????0??????????00???1110????1000111100?1????1??0????????00?00100??1????010100??100????????1??1???????0???00?1??1???????01?1?11011110?0?????0000?00?201?01?112023?2?111022?2?1???1?????????110001?1100?0?00010001??0000???01101111002000000111100?00?00???000???20000?000?????0??000????0????00000??1000??00?00?0?????0?0??0???????00?0?[12]0000???0?000100?00?0??010?0??00????????????????????0000??0000?0000??0?0?00?????000??0?0000?00?0???0111?11000????????01??10000111?1100?1000000?010100000000?0000000?0?00000001000200???0??0??1??00?0????000000000000??00?0000?00000001??1????0??000??001101?????????0??????????????????????????001000?010112010000???????100?0??????????????????????????0011101????00?000?????????0?01???????????????0?0???00000??0??00000?000?1??0?0?????10?????0?00?????0?0???0100??????0????00000????001?000?????0??000100?011?????0110????0???000????????0?

Microraptor_zhaoianus

0??????????????????100?????????????????????????????????????????0??010?0?1????0?01000000?????001?1??01?2100?01???0110?1212111?01?101111111010??110000011012111??0112023?2?1110221201?11111??00?0001110111110?100??000?11?000????1010111100?0?0???1?110???0?00??0?00???20??0?000???????????????????????????0?0???????00001000?[01]0?00?0000?001000200???0?000?01???10?0???10100?000??1?0?000?????????000???????0?00?0000000000????000?00??000000000??1211111??00?021???011011???1?1?1100?1000000??????????????????????????????0?????????????????????????????????????????????????????????????????????????????????????????????????????????????????????00???1?????????????????????00?????????????00001????????01???1?10000?000???0000??0?01??10?????0?00000?0???00????0????0?00??0????1????0000???10??0?00???0?010???????0???0?00??000??0?000000?000?10?000000010??????????0?1??0?????1???1??00?100?

Graciliraptor_lujiatunensis

???????????????????????????????????????????????????????????????????????????????020???1??????????????????????????0???11?12????????????????01011?1000001?????????????????????????????????????0??0?010?[01]11?1??0??0??00?????????????0???????????????????00???????????????200??????????????????????????????????????????????????????????????????????????????????????0??????10?0???000??0???????????10?0000??00?000??????????????????????????????????????????10????????????????????????????????000???????????????????????????????????????????????????????????????????????????????????????????????????????????????????????????????????????????????????????????????????????????????0????????????????????????????????1????00?00????????????????????????????0000????00??????100??0??0???????0?????????00?0?00?????01??????????????????0???00????????????????0?000?10????????0?0????????????????????????

Hesperonychus_elizabethae

?????????????????????????????????????????????????????????????????????????????????????????????????????????????????????????????????????????????????????????21??010?????????????221211?????????????????????1????????????1??????????0??1??????????????????????????????????????????????????????????????????????????????????????????????????????????????????????????????????????????????????????????????????????????????????????????????????????????????????????????????0???????????????????????????????????????????????????????????????????????????????????????????????????????????????????????????????????????????????????????????????????????????????????????????????????????????????????????????????????????????????????01000?0110?01?????????????????????????????????0???0?????1????????????????????????????????????????????????????000???????????????????????????????????????????????????0??

Pyroraptor_olympius

?????????????????????????????????????????????????????????????????????????????????????????????????????????0????????1????????????????????????001??????????????????????????????????????????????????????01??1?????????0?????????????0??????0???????????????????????????????????????????????????????????????????????????????????????????????????????????????????????????????????????????????010??0????????????????????????????????????????????????????????0????????????????????????????????????????????????????????????????????????????????????????????????????????????????????????????????????????????????????????????????????????????????????????????????????????????????????????????????????????????0?0??????????0?????????????????????????????????????????0???????????????0?????????????????0????????????????????????????????????????????????????????????????????????????????????????????????

Rahonavis_ostromi

??????????????????????????????????????????????????????????????????????????????????0???????????????011121???1?01?011112?12???????????0??11??011??????0?01111?10111120?3?2012101?121??21110001000?011101101?0100????0??1?????0??10001011111????????????????????????????????????????????????????????????????????????????0011000[01]0???????????????2????????????1100?????????????????????????010100???????????????000000?000000000100000000?00000000?001??01???0????????0?1?11?????1????????00?????????????????????????????????????????????????????????????????????????????????????????????????????????????????????????????????????????????????????????????????????????????????????????????????100?1??0?0??0011??????0?????0?100000????01??10???0?0????00?0???00???????????00??0?00?1????0000??0?0???????0???010???????????000?????????0000000?000?10??00?0001????????00?0?11?0??0??????????????00

Buitreraptor_gonzalozorum

?0010???????????????00001011?00??????????100?10??01?0?????????00??001??????????0210??100?0010111100111?110???100011012[012]121???????01101111010?1?1??0??????11?101??12[02]23??01?1?1?120???11?0??0??1?011?0?1110000?????0??1????????1101000110011000???0??????0000??0?00?10200?00?0???????????0???????0?000101???0?????00000000000[01]0??????????01000100?0?0?00??0?10000?0?001100??00000100000001?100???????????????000????00???0?00??????????0?00?000?0010?00??????????????1????0010??1100?0000???00?0????????1?11?00?0???010?0101?0??0????00000??1100????????0000???0?0??0??0??????????????100?????000??00?0?????????????????????0????????????????0?000???0?1?2????????????????000???????0001001000??0??00000111010100???0?001??000???????01000???00??0?????????0?0?000??????????00????0?1000100?0???????01000?????0?00010000000000????000000??0??01000?0??001???????10????11100?00010001???0?0???

Neuquenraptor_plus_Unenlagia

????????????????????????????????????????????????????????????????????????????????????????????????????11211111111???1????1????????????0??1001???????????01111?1011112022020111?121202011?10000??0?010??111100000????0??11????0??110110?110??????????????????????0?????220????????????????????????????????????????????0?00000?0?????????????????1????????????1100?0?0?0011000?00000????????????0???????????????00000?0000000000000000000?00000000?00????????????????????????????????????????????????????????????????????????????????????????????????????????????????????????????????????????????????????????????????????????????????????????????????????????????????????????????????????????11011?0?????0011???01???????00100000110?01?010000000?00?0100???00??????????000?00?00?1????10????01???????00???010?????????????00???0????000000??000010??0?000010?????????0??111001000????????????0?

Austroraptor

?0000???????????????????2?1?0????????12?0001????????????????????0?011???????????210??10?????0011?001????11???????????????????????????????00????????0???????????????????????????????????1??????0?0?0?0??[12]1??????????0????0????????????????11?00??01??00?????????????1[12]20??0?0??????????????????????????????????????0??00?00?????????????????????????????????????0?0?001100???00??0000000??????????????????????????????????????????????????????????????????????????????????????????????????00?0??????????0?10100?0000010??1???????1???????????????????????????????0??01?01????????????????????0?10?00????????????00000?000?00?????????????????0??0010000???????????????????000???????0001001101??????????????101???????????????????????????????000????????00?00????????????0?????0?0???????????????????????????0?0001000?????0???????0000???????00??????0?????01?1????1?????1?0??0001?????????

Shanag_ashile

???????????????????1??1010110?????????????????????????????????00??011??????????01001011????????????????????????????????????????????????????????????????????????????????????????????????????????????????????????????0????0????????????????21100001???00???????????????????00??0?????????????????????????????????????????????????????????????????????????????????????????????????????????????????????????????????????????????????????????????????????????????????????????????????????????????????????????1?10???00?0?000000???????????????????????????????????????????????????????????????????????????????????????????????????????????????????0??00??00????????????????????10???????????????????????????????????????????????????????????????????????????????0?0????????????????????0?????????????????????????????00????????????????????????????????????????????1?????????????????00???????????

Mahakala_omnogovae

???????????????101??????????????????????1?00???????0?0011??????????????????????02????1???00??0?11?011?211??1?100011012210????????????????00??1110000?100?211101?2??????????????????0111100????0?011001101?00000??00??11?0?????0000?????00????????0????????????0????1[01]2?0??????0?????????????????0??1?00???????????0000010000[01]0????????????????????????????10??[01]0???0?0000???0?????????????100????0??0???000000??0??001??0???000000000?00000000?001??00?000????1???0??????011?0????????000000???????????????????0???0???????????????????????????????????????????????????????????????????0?????000?00????????0???????????????00010?????????????????????0????????????????????0000?????0?01?000???????000??????????00????00100000???????0100000?0?000?100?0?000?0????0?0?0???0????1?10???00???????????0????010?20????0?0??0???????000000000??0??0100000000010?0????1?000???10????01????0??00???0

Atrociraptor_marshalli

???????????????????[02]00?0??1010????????????????????????????????00??010????????0?0000101001??????????????????????????????????????????????????????????????????????????????????????????????????????????????????????????0????0????????????????21?00??0??111?????????1???????????0??????????????????????????????0????0????????????????????????????????????????????????????????????????????????????????????????????????????????????????????????????????????????????????????????????????????????????????00001?1????1???0?0?000?01???????????????????????????????????????????????????????????????????????????????????????????????????????????????????0?????0?00????????????????????0???????????????????????????????????????????????????????????????????????????????0?0?????????????0??????0????????????????????????????000????????????????????????????????????????0??1??????????????????00???????????

Utahraptor

???????????????????[02]00?0??????????0?001??????????????????????????????????????0????0101??1????0?1100?1??1????????0??011?????????????101?1???????????0?????????0?01????????????11?????0111?00??0?001000100???10????????11??0???????0?????0???????????1???0???????0???10???0???0????????????????????????????????????????00000???????????????????20000?000000???????????????????????????????????????????????????00???????0??0000000000000?0000?????0?10??????????????????????????????????????00?????000001??????????????????????????1???0??00010?0????????????????????????????????????????????????????????????????????????????????????????????????????????????????????????????????????????????????????????????????????????????????????????????????01?????????0?0?????????????0?????0????????????????00????????????0???????????????????????????????0?0???????????????????1???????????????????????

Adasaurus_mongoliensis

?0010?????0????2??????????????0?????0?1??11?1?0?101??001??0?????0????0?1???0?????????????01100?11?0111?10?111[01]100??011?1?1??????1?111111??????????????1022111010102?2202?1?11221001?111000000?000?11010010010??00??00110?0?0?0001??00100?????????11????????00?0????1??????????0?????????00??00???000?0???????????000?0010000[01]0???????????????200?0???00??01110??????????????????????????????????????????????00?000000000000000000000000000?002??11??0???00???21?000??0??0001?1?????????0???0??0?????????????????????????????????0?????????1??00??????0000010??000??0??00?0?0?00????????0??????????0????????????????????????0??????????????????0???????11201????????????????????????????????????????????????????????????100000??0?01???????????????0?0???00?0????????000?00????10?????????????????????????00??????0??00???????????000000??000010000??00010??????????01?????????????????????0?

Achillobator_giganticus

?????????????????????????01?1??????????????????????????????????????????????????0000101??????0?01100?11210???????0??011?11??????????101????????????00??10220??11010102102?011011?001?21110?0????00?000?101????0???????1???0????001??00100021100??0??100????????0??????????????????????????????????????????????????????0000????????????????????20000?0?0?0?0??10??????????????????????????????????????????????00???0?00000??000???????0?0000????????0???????????????0??????????0???????????00???????????00?001???0?0?000??1????????????????????????????????????????????????????????????????????????????????????????????????????????????????????????????????????????????????10??????????????0100??????????????????????????100000??0101?0100000?1000??????????0?????????0??01?????1??0??0??????????????01??0?????????0??00?????????0000000???0000100???????1???????????????1?1?????0?00?????????

Saurornitholestes_langsto

?????????????????????????????????????????111????????????11????0?????????????????100101?00?11000110011121011011100?1011?1?1????????????111?????????000111221?1?1?1?2?0?????????????????11??0????00??001101??00???00?0?1??00?0000010?001000????????1110011??????0????1220???????0????????????????????0000???????????0????????????????????????????????0???00??1????????????????????????????????????????????????0?????????????????????????????????????0???00??????????0??????00111????????00?00???????????00?01??0?0?0?000?00????????????????????????????????????????????????????????????????????000??00?1???0?000??????????????????????????????0??0000000????????????????????0???????????????1?0???????????????????????????????????????????????????????????????0??????0????????????????0???????????????1??01????????????0?????????00???????????????????????????11??????????????????0???????????

Saurornithoides_mongolien

?00??1?1??1101???0?110001?1000????????2?????????????????1?010?100?0010??1????0001110101?????????1???0??1?????100??1??????????????????????????????????????????????02012020?010??10[01]1?11110???????????0???10?01??????0??1?0???????????010??11000??0?1?01?0?????0?000??????0??0000????0?1000?0??0???????????0000???0????00100?0????????????????????????????????????????????????????????????????????????????????00????????0?0????00000000000000?20?02100000000000201001?10???1110?1????????????00?0100000??2??11?0?0?1?0100010100??00???0??0?????0??0?????????????00????????????????????????????????????????0???????1????????000????????????????0?????????????????0???????00000??????????????0????????0?0?????????????????????????????1?010???????????00????00000????????00??00????0?0???00???????????1??????0??000?0?????0???????????????????0?00???????0?101?0????????1???0??????0?0?00???????

Zanabazar_junior

?00101?12?110100?001?000??100000????2022000?21??0??11100??????100?001???1????000111010100??????????????????1?1000?1020?1??????????????????????????????????????????2?????????????????????????????011????2???????????00??00???11????????0??110000000100000????1??00???????00?000?????001??0?00?????????????0000??0?00????????0[01]0???????????????????????????????????????????????????????????????????????????????????????????????00010000??000??2????1??0???00000201001?11???1110?1????????????00??100000??2??11?0?0?1?0100010100?000???0??0?????0??0?????????10??000??00?0??????????????????????000?00011010??????????????????000?00000????0000???000?001???????????0????00000???????????????????0??????????????????????????????????0?????????????0?0????????000?????????????0?????00???0?????????????????01?00?0000????????????????????????????????0?00????100100???????????1????000?0????????

Xixiasaurus

???????????????????01000101110??????2?22????????????????????0?00??001????????000201??01?0?????????????????????????????????????????????????????????00?1????????????????????????????????????????????????????????0???00?????????????????????1?000000???01?0000????00???????000000???00????????????????????????0?????????????????????????????????????????????????????????????????????????????????????0??????0????????????????????????????????????????????0?0?????????????????????????????????00????110100??2?01100?0?1?01000101000000???0??0?????????????????????????????????????????????????????000??0????????????????????????????????????????????010?000????????????????00000???????????????????????????????????????????????????????????????????????????????000??????0??????0????0?0??????????????0????????????0100???????????????0????????????????????????1?010??????1??????????000??????????

Byronosaurus_jaffei

?????101???101?1100110001011?0??????20220??????????1?100??????0000001??11????000211??01?0?0???????010121????????0??02????????????????????????????????????????????????????????????????1????0?0??0???????21??????????0????0????1???????????11000000?1?00????0??0?000??0???000000????????????0??????????????0?0????0????0010?????????????????????????????????????????????????????????????????????????????????????????????????000?????????????????????????????000200011??0????????1????????????00??100000??2?011?0?0?1?0100010100??00???0??0?????0??0?????????????00????????????????????????????0???????????0??????????????????00?000000????????0??00??00?????????0???????0?0000??????????1???????????????????????????????????????????????????????????????????000?????????????0????000????????????????????????00?0100?????????????????????????????00?????????1?0?0?1????1??????????000?0????????

Sinornithoides_youngi

?0??01?????????????1?000??1??0????00???2???????1??????????????00??0010???????0001110?01??????11??001???????????0011?102121011???1?00?1?101?0???100000????21??01??02??30???11?0??001??1110?????0???1?000110001?0??0?0?1??00?0???0???0010?0??00000????0??0??0???00???0?2000000?0????????????????????00?????1?00??000??????????[01]0?0????????0??0?20??0?00000?0??1000???0?1000???00?0?????????0??010?0000???000000??00??0??000????00000000000000020?02100000000????????101??????1?010100??1??000?????????????????????????????????????0????????????????????????????????????????????????????????????000??0???????????????????????????????????????????????????????????????????0?0?00?????????0???????????????00?110101?000?000???????????????1??????0?????000???00?0???0???0?00??0??1????0?????100????0?0????????0???000?000???00??10?000?????0??00???00000000010????0?1?0?0101????0????0?????0?0???

Mei_long

?0????????0????????11010?????0?11?002012100001?1010??100??????00010010??1?0000?021???01?0011011100011?2110?01???0110222111?1?????111?11100?0?1?1000001?012111?1??1200302?1110221?[01]?0111100?0101001000??110011?0?0000???00000??000100?1??0?1?000000?001?0?0000?000001020000?000?????????00?0??0??00??0101????????0?0000010000[01]0?0????????0100020?00?00000?0111000?0?0000000?0000000?000?01?1?0???00000????00000?00000010000??00000000000?000021?01100000??0???20???1010??101100?1100?10?0000?110??0??0??????????0?1?01????0?????00???????????????0?????????????00??????????????????1???0??????0????0????0????????????????????????????????????0?000?????????????0???????0?0000?????????0?????????????????????1?1?0????????????????????????????0??????00???00?00?0??????0????0????1?0?000?1001??00?0001?0?010???0???0?0000000000?000????00???0???00?000000100?????????01??????????0?0????0?????

Jinfengopteryx_elegans

?0?????????????????1?01???2000????????1??00?0????1???100??????00?00??0??1??0?000?1???0???1??????????????????????02????2?200???????1101110??????100000?????1??????????????????2??0??????????0?????????????????0?0??00????00??????????????01?000001???0??0?0??????0????200?0?000???????????????????????????0??????0?000???0?0??0?0??????????????00?0???0?0?01???0??????????????????????????????????????????????????????????????????????????????????10?0000???????????0??11?01?????????????000?110?????0?01??1????0?????????0?????00?????????????????????????????0??????????????????????????????0????0????0????????????????????????????????????0??00??????????????????????????0?????????????0????????0????1??0????00????0??????????????????????0???????????????0?0??00??????00????1?0???0????????0????????????????000??????????????0?0???????00?????????001?0?????????????????????000????0?????

Anchiornis_huxleyi

000100?????????????11010??11100?????0?2?000??1???11???????????000??01??1?????00020???01??0?101???00?0??010?01???01101220??01?????011111110000??100000010?21?10011020?202?1?1122121???1?1?000??1?01?000121100010?1000?1??00?0???????0?11?011000001???0??00?????000???030000?000??????????0??????0????0??????0??????00000?0000[01]0?0????????0000020000?0?00??0?10000?????10?0???0????????????????10?01000??0?00?00??00?00000????????????00000?0?00??0100000000????????00??1110?1?1?1100010??000?110??0??0?01?010?0?0?1??101?00??0??00???????????????0?????????1???000??0???2??????????1?????????00????0????0????????????????????????????????????0?000??01?????????0???????0?0000?????????0???000??????000??????1???00??0000100000??0?01?????????0?????0?0???00?00?0??100?00??00???11?0?0000???????0??????????0????1000????0????0??0?0000000???00?0???????00100???0???0?01??????????000???00?0?0?

Xiaotingia

?00?????????????????1?1???111000????0?2??????????11????????????001001??1???00??020???01??0??0???1?0?????10?01?????1??????10??????01?01111?10???1000001011?1110?1102122?????102?12[01]??????????10????????1?010001010000?1??0?10??00???0010?011000??1???00??0?????000???22000??000?????????????????????0000?????????0??000?10000???0????????010002????????????11?000?0?00???0???0?0?0????0???????????0100??1?00000???0?0000???????????????????0??0?02????10000????????00??1??????1?1100?10??000?110??0??0?01??1????0?????0???0?????0????????????????0?????????1???00?????????????????????????????0????0????0????????????????????????????????????0??00???1?????????0???????????00?????????0???????????????0011??????0???000010?000???????????????0???????0???00000?0??100?00??00?1??1?0???????0????0??????????0?????000?????0???0??000?000?????0??????????0?100???0?????0101????0???000???01?0?0?

Aurornis

?0010??????????????0101???11100???000?1?0??????????????????????00?001????????0?020????1??0????????0????????0????0??0?221?00??????01?01111??0???100?0??001?011????02122020??10???????????????????????0??100000?000000?1??0??0??00????111?011?0000????0???000???0000???20000?000???????????????????????????0??????000??0???000?0??????????00???2??0????????0?0?010????????0????????????????????10?0??00??0?00?00?0000000????????????????0???0??????1??010000????????0???1?1?1??1??1000100?000?110??0??0?01??10???0?1??101?001???000???????????????0?????????10??000??0??02???????00???????????00????0?????????????????????????????????????????0???0???1?????????0??????????00???????????????????????0???01???1???00??0000100000???????????????0???????0???00??0?0??100?00??00????1?0???0????????0??????????0?????00??????0??????0?000000???????????????00100???????????0?????????00????00???0?

Eosinopteryx

00010??????????????1?01??????000????0?1?0?????0??1?????????????00?001????????00020????1??0????????0?????????????0??0?231?10???????1101111??0???100000?001?1?1??1?02002?20??10???????????????????????00?1??0001000000?1??0??0??00????011?0???00??????00?0?00???0000???20000?0000??????????????????????????0??????0000?0?10000?0?0????????00???2??0????0???0?1?010????????0???????????????????????0??00??0?00?00?000?000????????????????0?????00??01???000??????????0???11??1??1??1??0??0?000?11???0??0????????????????????0?????00???????????????0?????????10??0??????????????????????????????0????0?????????????????????????????????????????0???0???1?????????0???????????0??????????????000??????0??0011??1???0???000?100000???????????????0?????0?0???00??0?0??100??0??0??1????0?0?0????????0??????????0????100??????0??????00000000???????????????00100?????????0?01????0????0????00?0?0?

Troodon_formosus

???1?1112?1101000001???0?011?0??????20220000210?0??01100????0?10??001??????????0111010100???1111100101211111?1000?1020??11?????????????1?010?????000010??????????0?0?2?20?11001?0??01111000???00010000021??01????000??1?0??010???1??0??0?1?00000?0?0???????00??????????????????????001??0?000?????0??1?1?????????00???????????????????????????????????????????????????????????????????????????????????????????????????????????????????????????????0?????00000201001??0???1110?1?????????0000??????????????????????????????????????????????????????????????????????????????????????????0?????0000??00110????????????????????0000001000??000000??0000000????????????????????00???????0?0??00?????????????????????0?????????????????????????????????00000?000??0????????00??0???0??0????00??01??0000?0?1??01?01?00?0????????????0?0?????????????????0?000?10?0?10?0?0?0????????????0??0????????

Sinovenator_changii

?0???0002?000011110010101?1110?011?02??2??0011???1?10100????0?000?001??????0000011110?1?????11?10100012110?001000110222?1?????????110111???????100?0???0?211?010112[02]0302?1110221201?11110000??10011000111??110?000000???0000110001000110011000??1??011??0000?00000??0??00000000000?000000?00?0??0000010100[12]00??00000?0010000?????????????????20000??00?0101?10??????????????????????????????????????????????00?1000000100000000000000?0?00?020?0110?00000000020?010?10??1011000???????000000?????0000?02?011??00?1?0100010?????0??????????????????????????????????????????????????????0????????0?00????1???????????????????0????????0???00000??00???0????????????????????00?????????????00????0??????00111?????????????0000?0??0?01???????1??0????0?0?0???0?0???????????0?0?1?1??0?????100????????0????0??01?01?0?0000?00??????0?00??0???0000?0000000001?1???0?????0??1??1?????000?0????????

EK_troodontid_IGM_100_slash_44

?????0012???????????????????????????????????????????0???????????????1??11??????????1??????0????????????????????????????????????????????????????1?00000???????????????????????????????????????????????01?1?001???00??????0?0??1?????????0?????????????????????????????????????????????????????????????1?1??????????????????????????????????????????????????????????????????????????????????????????????????????????????????????????????????????????????????????????????????????0?????????000???????????????????????????????????????????????????????????????????????????????????????????0?????????????????????????????????????????????????????????????????????????????????????????????????????????????????????????????????????????????????????????????????????????????????????????????????????????0??????????1????0????????????0000????????????????????0?1??????????????????????????????000???

IGM_100_slash_1126

?0????002?0000?11[12]01?010??2000?011?020121000110?01010100??0??000000000?11?000000211??00?0??????????????????0????01102???????????????????????0??1000001???211?010?020020200010131012??111000????0??2?001210001?00000011?00?0?020000?00100?1?00000111?01?0?00?000003??????000000?????00??00?00?0??0??0000?00?00???0?0?????????0????????????????????????????????????????????????????????????0?00???0000???0100000?00??0???0?00?00?????00?1?000?20?02????0000001020?111??0??101?001???????0?000?????????????????????????????????????????????????????????????????????????????????????????????????????????????????????????????????????????????????????????????????????????????????????????????????????????????????????????????????????????????????????????????????????????????????????????????????????????????????????????????????????????????????????????????????????????????????????????????????

IGM_100_slash_1323

?00???????0???11???1?000??20000?1??020121?00?1??0?0??????00?0?000?0????1??00000021???0000???????????????????????011???????????????????????????????????00??1??????020?20200[12]102?1011???????00???0????0??2???01??00??0????0?0???00?0?00100?1?000000?1?01?0?00?00?003??????0000000????00?000?00?0?????0000?0?000??0?00?????????????????????????????????????????????????????????????????????????????????????????00?????00?00?0?00?????????1000??2????????????????????????????01?????????????????????????????????????????????????????????????????????????????????????????????????????????????????????????????????????????????????????????????????????????????????????????????????????????????????????????????????????????????????????????????????????????????????????????????????????????????????????????????????????????????????????????????????????????????????????????????????????????????????

Archaeopteryx_lithographi

100?0000??000??112010010??1110?011000012100?10?0000??100111?0?000001000002?00000200??00100?1?1???00?0??1?0?0???0021012311000????1011111111000?110000000112111010?12003020121022?2?2?111100000?0001000000030?0000000001100000??00?000110011100001001?01000000???0000?030000?0000000?00110??00?0??000?00??000???00?000000000[01]000?0????????00000?0000?000000010000000?0011000?000??000?0000100000??00000?000000000000?0?00000000?00?0?[01]1000000?00???00000100001020?00001011101?0101100?10000000110?00000?01?01?0000?0?0100?10100?000???0000?01??00?0????00???10??000??????????000000?0?0????????000??0011000?????0??????????000000011??????00000?000000000??00???0???0???0?0100???????000?0000????????0?0011101?10000?000?100000??0?01?01??????0????00?0???00?00?00?100000?000?1?1000?0?0010010??000000?0?010020?1000?000000??0?000000?0000?000?????0?0000100001001???0111????00?100010?000000?

Confuciusornis_sanctus

10010??????????????1?000?00??0001???0??2??0??0??00??01???????000010000?10?0001?1?????????0??????????1021?0?2???0?2????4??111?11010??13111000?11110000001121?1?1??12000?2?111023?2??12??10??11??120211010030?0000000001102000??00?0101??11?100011001??????0?????000?123001[01]00120?????????10??00??011200??01[12]0111110000001100001001000120000?00?0000?0??0??010000000?000211??0010000000010102101011[01]?10?0000001010000000001010100100011000011000?0000000[01]000???2000001101110?00??1100?10?0000?110?00000??1?00?0000?0?01?0?10100?00??????????1??00?0????0000000??000??01?02???00100000?0????????000??00???0????????????????????????????????????00010???0?0??00??10????????????0???????0001?000???0????0?00111?0?1?000?100??????0??0??1?????????0????00?0???00?00?01?100000??0??1??0?????0?????0??00???????010???????0?????0?0000?0?0??????0??0??????0?0000100?0?0?????01???????????1????1110111

Jeholornis_prima

10????0????????1???0???0?????001??????????????????0???????????0000?1???00?00?1?1?????????0????????0????1?0?11???02111031??01?0???011031110001??1100001011?1??0??01?0???2???1?221?10???????01????0101101003000000000??0??1000??00?010????1???000?1?????0???0??0??0??12300?10010??????????????????0????00?00000??00?000000100000?0??001???0000?00000?0?1000120001000?0010000?000?0000?00001000010110000?000000000000?00000?010000000001000001000?00000001100????????011?1110???1?1100?10?0000?110?00000??????????0?0???????0??????????????????????0??????????0??00?????????????????????????????000??00????????????????????????????????????????0??00???0??????????????????????????????0?0??0000???????0?0011101?1?0???000???0000??0?01?????????0????00?0???00?0??01?100000?00??1?10???0?00???????00???????010???????0?????0???0??0?000?00???000?????0?0000100???0?????0??????????1?1?1??11?0?0?

Jixiangornis_orientalis

100?????????????????0010?????001????0?0??00000??????0100??????000001????0?0??1??0????????00?0???1?00????1??20???02??1031??11?1?000110311100011011000011112101010212000020101?221?10?2?11?001??1?01[12]1101003000?00?000?0??1000??00?0100??111?001?1?????????00?000000?12300?00010??????????????????????0?????000??00?0000?11?0?10?11?0?????0000000000?0010011210010?0?0000000?000???0??0????0??010?00100??0000?001000000?00??0000??00000100000?00?0000?001100????????00??11????01?1100?10?0000?11??00000??????????0?0???????0??????????????????????0??????????0??0???????????????????????????????????0?????????????????????????????????????????00010???0??????????????????????0???????????????????????????????0?1?00??00???????????????????????0?????0?0???00?0??01?100?00??0?????0??????????????00?????????0?????????????????0??0?0????????????????????00100???0?????0????????????1????11?010?

Yanornis_martini

?00????????????????1?010?????0?11???0??2??0??????00???????????0000011???0????000?1???00??0????1???0????????4?1???????04??????110?011031110?011?110310????????????????????????23?010?2??10??1??0?0121[23]0000300000??0001???00?0???????0???21?1?0??????????????????0?????300100012??????????10???????1?????????????????0?0011?00???0210?[12]??10100001010?1?11?112100111??0101?0101?10000?0?????021[01]101311[01]1100[01]100?0?1?0??0?00??????0??00?1110[01]?1?00??1?0??01100????????01??1????????1100?11??001?11??????0??????????0?0???????0?????0????????????????0??????????0??0?????????????????????????????????????????????????????????????????????????????0???0???0??????????????????????0???????0?0??0?0??????????00111?0?1?00??100???0000??0?01?????????0?????0?0???00?0??01?100000??00?1??0??????????????00?????????0?????000?????0????????000?00???????????????00100?????????0??1?????????0?1??11?0100

Apsaravis_ukhaana

???????????????????????0??????????????????????????????????????0002?1?0??0????????????????1??11??10200??1???5?100021??24??????1?0101103110000111110?3??000?1???1?201000?2?021?23?03?12?010?????1??12130100?00000??20???1?20?0?????0?00??21????????????????????????????300?10?????????????????????0?1[01]?????1?0????????20010?[01]001??21??1????????00000?0?1000121011111?01011010[12]0111110000101021110?[23]120100?11??20?1[01]0010?11?110?1012112111111?0000?1?0??01101????????011??????0?1????????0001?????????????????????????????????????????????????????????????????????????????????????????????????????????????????????????????????????????????????????????????????????????????????0?????????01???0?????0??0?0011100010001?1?0?1?0000????01?010??????????0000??000?????1??????0??0??1????????00?00100?????0??????0???????0????0000000????00000????000????0?000?10????????0?0?11????0??????????1????1

Yixianornis

100?????2?021??1?????0?01????0?1?1?0????????????????0100??????000?010???0?00?00120???10?01???1???0000??1?0?40??0???0024???11?1100011031110?0010110300100021?101?212003020001023?23?0211?0??1??????2130100100000??000?0??1000?20??0?000001???????????0??0??0???0??0?1230010001???????01??1????????11[01]?????1?0?????10?20011000?11021??[12]??10100001010?11110112100111100?0110???01?000???0?01021[01]10?31201100[01]100?00100?00?002?101??????11110[01]?1?00??1?0?001100??????00011?11?01????0100?10??000??1?????????????????????????????????????????????????????????????0?????????????????????????????????0??????????????????????????????????????????????????????0??????????????????????0?????????????00?0????????00111?0???000?100?1??000????01?????????0?????0?0???00????01?100?00??0??1????????0???0????00?????0???0???????0?????0???0????000?00????00??????0??00100?????????0?11????0??1??????1110100

Sapeornis

?001???????????????0?010??11000????00??21?0?00????0??????0????0000010??10?00000020??????00????1???000????0?211??0210004??100?????211?1111010011110200101121??00?212000?201[01]00221210????1??01??1001210?10030000000000????2000??0??0100??0111?000???1???00??0???0000??23000000000??????????????????????????0?00???0000000010000100????????0000020000?00000?010001000?0?01010?0010000?0???010?1[01]10?11100??0[01]00?0000?0000?00?000??00?00??000001?00??0?00001000????????001?1?1011?1?110001000000??1?000000??????????0?0???????010??00?????????01??00?0?????????00??0??????????0?????000??0????????000??00????????????????????????????????????????00000???0?????0???0???????0?0100???????000??000?0??????0?00111?1?1?000?000???0000??0?01?0???????0?????0?0???00?0??01?100000?000?1?10?0?0?0??0?1???000000?0???0????1??0???0?0??????0?000?000??000?????000000100?0?0?1???00?1?????????1?1??1?1011?

Neuquenornis_volans

???????12????????20?????????????????????????00?????00??????????????????????????????????????????????00??????????????????????1?11011?1031110?0111110????????????????????????????????????010???????????[01]010030??0????0??????0?00???????????1???????????????????0???????0200?????????????????????????????????????????????0110???????21?????10110??0100?001?001?0??[12]??1???01?0???0?????[01]?????????2???20?1??01????????????????10?1????????1?0???1011???????0?000???200000110??????????????????0??0??????????????????????????????????????????????????????????????????????????????????????????????????00???0???????????????????????000??0100??????????????????????????????????????????????????????????????????0111?????0??????????????????????????????????0?0???00?????1?????00??0??1???0??????????????????????????2???????????????0?????????????????????????0010?0????????0??????????????????1???1?

Patagopteryx_deferrariisi

?0????????????????????????????????????????0010????????????????????????????????????????????????????2????????????????????????????????????????0?1???????????211101?????????????1???????2?010001??100121301001?000???????1????????00?0?????1??????????????????????0?????0??????????????00?1??????00??111110000?????????010000000????????0????????000????0101?1210[01]00???[01]?10?0???01000001[01]0001?[12]00???30???????00?2000000?010120????0?000[12]11001100000?[01]?0??0?100?1?200??01?0???0?001????????00???0?1????????????????????????????????????????????????????????????????????????????????????????0??????0?0??0?110????????????????????0??????????????????????????0020000??00??????????0?0?00??0001?00000????????00111?00100???????1?0000???????0100??0?0???0?000???00????0??????00??0??1??????0?10???????????00???010???????00000000??0?????000000???00?1???00000010??????1???0????0???0?1???10??1???0?

Cathayornis

?0?????????????????1?010?????0?1??????????0???????????????????00?????????????00001???????0????????[02]????????3?????2????4???0??11?02?1031110?011?1102?0?01111?0?00?12000?201?1022?23?????????1???0??[12]1[01]1?0?3000?0???00????0??1??0??0100???111?????????0??0???????0????2300[01]0?012???????????????????????????0??????0????0111??0010?20??1??10110?101????010?01200010010110[01]?01020111110?[01]?1?????210120?11?01?0101011?0?000001???1?0?0?1[01]1?0???????????0??01110????????01????????01??????????0???110?00000??????????0?0?0100?10100?000???????????????0??????????0??00?????????????????????????????000??00?0?0????????????????????????????????????0??00??00?????????????????0?0100???????0?????????????????00111?0???0?????????0000???????????????0?????0?0???00?0??01?????00??00?1?1??0???????????????????????0????100??????0???0?????00?00???????????????00100?????????0???????????000???11?0?1?

Concornis

????????????????????????????????????????????????????????????????????????????????????????????????????00????????????????????0??11?02?1031110?0?1???0230????????????120?102??11?23??3?0???1???1???0?1[02]11??0?3000?0???0????????????????001??1???????????????????????????2300?????????????????????????????????????????????0111????10?20??1?0101100101?0?0010001?0??[01]001?110??0102?1???10???1[01]1?[12]??????????????[01]????????????00?????0?100??1?00??1?[01]1??0??????110?????????????????????1100110??01????????????????????????????????????????????????????????????????????????????????????????????????????????????????????????????????????????????????????????????????????????????????????????????????0??????????00111?0?1?0????????????????????????????0?????0?0???00?????1?100?00??0??1????????????????????????????0?????????????0????????0?????????????????0??0010??????????0?????????????????11?011?

Gobipteryx

?0?????????????????1?01010?0?0?????????????????????????????11?000?0??????????1?1?????????????????????????????????????????????????2????????????????????????????????????????????????????????????????11???????????????????????????????????1????0??????????????????0????????1100100?00110?10?????00?????0???0??0???1?????????????10??????????11??10000?0?100012000[12]001?1?0110???0????????????1?121??[012]0?????1?0??1????????000?0???10?001111000?1?11?0????????????????????????????????????????????????00000??????????0?1?01????0???0?0????????????????????????????????????????????????????????????????????????0????????????????0??????????????????0?01000?000??00???000???????????????????????????????????????????????????????????????????????????????????????????0??1?????????????????????????????????????????????0???????????????????????????????????????????0???0??????????????????0???????????

Vorona

???????????????????????????????????????????????????????????????????????????????????????????????????????????????????????????????????????????????????????????????????????????????????12?010011101001112110???00????????????????????0?????2?????????????????????????????????????????????????????????????????????????????????????????????????????????????????????????????????????????????????????????????????????????????????????1010111100001?000000??0??????????????????????????????????00?????????????????????????????????????????????????????????????????????????????????????????????????????????????????????????????????????????????????????????????????????????????????????????????????????????????????????????????????????????????1???????00000000?00??????????????????????????????????????????0?10001????????????????????????????????????110?00000010??????????0????0?????1???1????????0

Songlingornis

?????????????????????0?0??????????????????????????????????????00?????????????000?0????0??????????????????????????????????????11??[01]???3?????????????????????????????????????????????????????????????????0????0?????0?????00?????????????????????????????0???????0?????????0?01???????????????????????????????????0???????????????21??[12]?000100?010???1?11011????????????????????????????????????????????????????????????????????????????????????????0??0?????????????1??1?????????????????????????????0???????????????????????????????????????????????????????????????????????????????????????????????????????????????????????????????????????0??00??00?????????????????0?0?0????????????????????????????1?1?????0????????????????????????????????????????????????????????????1??????????????????????????????????00????????????????????????????????????0?1????????????????????????0???????????

Pengornis_houi

?0010??????????????10010??0?00??????0?12??0??????0?????0??????000?001????????0?0?00??00???01??????20???0???2??????????4????1?????2?1031111?0??????23??001?1??????????????????2??2??????????1??????211???0?000??0??000???0??0??00???0???11?1?000??????????00????00001230000?000?????????????????????????????0?????0???0011??00100????????01[01]00?00??????0??1??012111[01]0?011010001[01][01]1?????1??1???10[12][12]0??1??1?11??????0????00?0????0??0[01]?1?00??1?11??0?0??0?100????????01?????00?01?1001011???1??110?00000????0??0??0?0?0100??0?????00???????????????0??????????0??000??????2?????????????????????0?0??0?????????????????????????????????????????0?0?0???0?0??00???0???????0?0100??0?0??0??1?0????????????00111?0?1000????0???0000???????????????0?????0?0???00?0??0??????00??00?1??0?0???0????1??????????????0????1?00?????????0??????0?0?????00?1???????00100??????????1?1????????000??????0?1?

Hesperornis

?0?????????????????00010100?00??????00021?001????00???00????1?000?001???1?0001?021???00??10101110?2100??11?50100?210?24??11??10??00103111??????????3??0202100?1??01000???021023?03?12???0001??11?1213110010??0?00??01?1?0000?000???00??2??1?1111????0???????10000??10?1?10?1121200110?1010??1110011100??00?0???1010121011000012?0?000100100?0???10?0???00011??0?????????0???0????0??????????????????????????20?1100001112110211110021111122122002?0?????00??????0001?0???0?000?0100?11???1??01??00000??????????0?1?0100??0?????00????????0???0??0??????????0??00????????????????00??0????????0?0??0????0????????????????????????????????????0?000???0????????????????????000???????0?0??000???????0???01?????????????201??000????01?????????0????00?0???0??00?0??????00?????1??0?0?0?0???0???????????????0?????000?????????0?????0000?????00?????????001?0??????0???1??????????000?????????0

Baptornis

??????????????????????????????????????????????????????????????????????????????????????????????????2????????5?????????24???????????????????????????????????????????????????????????????????????????212??????????????????????????????????2????????????????????????????????[12]????????????????????11001??00??1??????????121011000012????012?0?????01?10?0???000????0?????????0???0??????????01?[01]1[01]???????????????20?11000011121102111100211111?21020?2??????????????????????????????????????????????????????????????????????????????????????????????????????????????????????????????????????????????????????????????????????????????????????????????????????????????????????????0???????0?0??000???????0???01???????0?????201??000????01?????????0????0000???0????????????00?????1??????0?0???0???????????????0???????0?????????0?????0000?????00?????????0210???????0???????????????????????????

Ichthyornis

?????00????????1220?????????????????????000111????0???????????000??1?????1000??0210??10???1?01111?2100?1???5?000?21??34???01?100?001031110?011111023??0102?1??10212000????21023??3?12??10011??11?1213110???00?0??20?????00????0??00????21???????????0?????????????012300[12]0?11???????????10[01]1?111011101001020???101?1200111?101[12]?211022010100001010?1111011211011110010110111010000101[01]10112111123120110011?12001[01]0010?112110211110021111[12]2?100002?0??011?100?20???0110??1??00?00100?11??01??01???????????????????????????0?00?00??????????????????????????????????????????????????????1?0?????00???????????????????????????0????0100?????0?00?0000?00?????????000?0???????00??000??0?01?0000??????0??00111?00100?0?10201??000????01?0???????0????0000?0?0???0?01?1???00??0??1??????000??00100?0???001??01??2?0?00000?00000000????0000?????00?111?0?00001????10?1?0?0?11?0??0????0??0?11?????

Iaceornis_marshii

?????????????????????????????????????????????????????????????????????????????????????????????????????????????????????????????1101?010311??????111??3????????????????????????????????2??1??1???????21?????????????20???1??0??????????????????????????????????????????????????????????????????????????????????????????????????????21?02[23]11???1101010?11110112101???????????????????????????????11131401110111020010??1?1?12110212110021??????????????????????????????????????????0100?11???1???????????????????????????????????????????????????????????????????????????????????????????????????????????????????????????????????????????????????????????????????????????????????????????????????????????00111???????0?10???????????????0??0??0??????0???????????????1??????????1???????????00????0???00???01??????????????0??????1??????????????1???0?00????????????????11?0??0????????????????

Limenavis_patagonica

???????????????????????????????????????????????????????????????????????????????????????????????????????????????????????????????????????????011?11????????????????????????????????????????????????????????????????20??????????????????????????????????????????????????????????????????????????????????????????????????????????????????????????????????????????????????????????10000[01]010101011?11[12]313011?011????????????????????????????????????????????????????????????????????????????????????????????????????????????????????????????????????????????????????????????????????????????????????????????????????????????????????????????????????????????????????????????????????????????????????????????????????0?0????????????????????????????????????????????????????????????????????????????????????????????????????????0000???????????????????????????????????????????????????????????????

Lithornis

100????????????????01010100?00?111??2?02?00?0???????????????10000?00????02?101?1?????????1????????2????????[67]?1????????????[12]??11000010311110011?11023??000210101?20200102??2102?????12?01000???11?12130100?????0??200?01?2010???0?0?0???21???12???????????00???00000??300?11212100?00011111010110?11[01]1101?1111?01?10121012?0111[12]1211122110100?01011011110112101111100110001[12]1110000101010101111113130110[01]11102[01]0120110?11211020110002111122210010[01]?0?0011?1????????011?111??00???????????01???1??????????????????????????????????????????????????????????????????????????????????????????????????????????0????????????????0?????????????????????????????????????????????????0???????0?0?????????????????????????????????1??000?????????????????????????????????01?????????????????????????????????????????????????0???????????????0000????????????????????????????????????????????????11???0?

Hongshanornis_longicrest

101????????????????11010?????0?11???0????00???????????????????000?001??00????1?1?10???0??0??01??1?0????????[234]??????????4???1??11?011103111000111110220?0112???01?20????????2??23?23??2??100?1??????21[23]01000000000?000?01?1????????0?0????1?1?0????????????0????0000?1?300???012???????????????????????????00??????1?????0?????110????????01?0??11???0?10???21000111???110???2????0????????????1??2000??000100?0??0?0??000????????1???1100??[01]?00??2?0???1????????????????????????0100?11??001?11??????0??????????0?????????0??????0???????????????0??????????0??0???????????????????????????????????0?????????????????????????????????????????0?0?0???0?????????0????????????0???????0??????????????????01?1?0?000?0?10???????????????????????0?????0?0???00????01?100?00??0?????0??????????1???00?????????0?????????????????0??0?0?????????0??????????00100?????????0????????????1????11?010?

Liaoningornis_longidigitu

?????????????????????????????????????????????????????????????????????????????????????????????????????????????????????????????????????3??1??0???????????????????????????????????????????100?1????0?21211003000?????0??????????????????????????????????????0?????????????????????????????????????????????????????????????????????011?01??????????0???0?????????????????????????????0?????01???2??????????????????????????01?1?0??001111110201000?00??????????????????????????????????????????????????????????????????????????????????????????????????????????????????????????????????????????????????????????????????????????????????????????????????????????????????????????????????????????????????????????????0????????????????????????????0?????0?0???00???????????00??0???????????????????????????????0???????????????????????????????????????????0010??????????0??????????????1?????????

Crypturellus_undulatus

10????012?020002?2001010100?00?111?12002100001011100010???1?10000?000??102?001?1?????????1110111122100?01107?110??12?24??121?11010010311011011111033??001210001?202201120021023?23?12?01000100110121301?0???01000200001?20000200?0?001021???120??01??????0????00000103002112121[01]010001111101011001101111111110010101210101121121211112?10100021011010111112100111110110001201100001110101011111031401011111020?120110?11211020211002111122210010110??011?10102000001111110000??1100?10??01??11????????????????????????????????????????????????????????????????????????????????????????0?????????????????????????????????????????????????????0????????????????????????????????????????????????????????????????????????2??????????????????????0??????????????????1????????????????????????????????????????????????????????????????????????0?????????????0??????????????????????????????1110?0?

Gallus_gallus

100???102?021002?2000010100?00?111?120021?0001?1000001????1?100100000000021101?1?????????101[01]1111221?0?01007?120??12?24??1[12]1?11012010311020011111033??001211111?2?2001120021023?23?12?0100011011??213010010001000200100?20100200?0?0???21???121??01???0??00???000001030011021212111112211011111111111101100011010111210101120111211112110100021011111111112100111110110001201100001011101011111131401001211021?121111?112110202110021111322200111?0??0110001020?000111111?000??0010110??01??11????????????????????????????????????????????????????????????????????????????????????????0?????????????????????????????????????????????????????0????????????????????????????????????????????????????????????????????????2??????????????????????0??????????????????1????????????????????????????????????????????????????????????????????????0?????????????0??????????????????????????????1110?00

Crax_pauxi

1001011?2?021000??001010100?00?111?120021000011100000100??1?100000000000021101?1?????????1000111122100?111?7?120??12?24??121?11002010311121011111033??0002100?1?2?2001120021023?03?12?0100011011?12130100100010002001?1?20100200?0?0???21???121??01??????00???000001030021021212111112211011111111111101100011010111210101120111211112110100021011111111112100111110110001201100001011101011111131401011211021?111111?11211020211002111132220011010?0011000?020?0001111110000?11100011??01??11????????????????????????????????????????????????????????????????????????????????????????0?????????????????????????????????????????????????????0????????????????????????????????????????????????????????????????????????2??????????????????????0??????????????????1????????????????????????????????????????????????????????????????????????0?????????????0??????????????????????????????1110?0?

Anas_platyrhynchus

100???102?021002?2101001100?00?111?10002??00?1?100010?0???1?10200000100?021101?1?????????101?1111221?0?011?7?11???10?2410121?11011110311111011111033??0012000?1?2?2001120021023?13?12?0100011011?121311?0???010002101?1?20100?00?0?0?1021???121??????????00???000001130021021211111112211021112111111101100011110111210101020111211113120100101010?1[01]111112100011100110001201100001011101111111231401010111021?111110?111110102110021111[23]2210010210?0011?10?020?000111111?000??0100?11??01??11????????????????????????????????????????????????????????????????????????????????????????0?????????????????????????????????????????????????????0????????????????????????????????????????????????????????????????????????2??????????????????????0??????????????????1????????????????????????????????????????????????????????????????????????0?????????????0??????????????????????????????1110?0?

Chauna_torquata

000??1012?0210002?101010100?00?111?100021?00010100010100??1?100000000??0021101?1?????????1011101112100?111?7?11???1012412111?10000010311111011111133??0012101?1?2?200102?021023?23?12?0100011011??213010010000000200101?20100000?0?0?1021???121??01??????00???00000123001102121[12]111102211011112111111101100011110111210121020111211114010100101011111101112110211100110001201100001011101011111231401011111021?111111?11211020212002111122220010010?0011000102000101111110000?11101?11??01??11????????????????????????????????????????????????????????????????????????????????????????0?????????????????????????????????????????????????????0????????????????????????????????????????????????????????????????????????2??????????????????????0??????????????????1??????????????????????????????????????????????????????????????????????????????????????0??????????????????????????????1110?0?

Pedopenna

????????????????????????????????????????????????????????????????????????????????????????????????????????????????????????????????????????????????????????????????????????????????????????????????0?0?0??0010??????????????????????????????????????????????????????????????????????????????????????????????????????????????????????????????????????????????????????????????????????????????????????????????????????????????????????????00?000?????0???????00????????0???11???????????????0??????????????????????????????????????????????????????????????????????????????????????????????????????????????????????????????????????????????????????????????????????????????????????????????????????????????????????????????????????????????????????00????0???00???????????00??0???????????????????????????????0???????????????????????????????????????????0?10?????????????????????????????????0?

Epidendrosaurus

??????????????????????????????????????????0??1????????????????10??????????01?????0???????0??????1??????????????????????????????????001??1??0??????001??????????????????????????????????????0????????0000030?0?0???0?????1???????????????1????????????????????????????3?0??????????????????????????????????????????0???????0???????????????????0??0?0??????1???0?????????0???0???0????????????????0??????000??????????????????????????100?00000??0?0??0?010????????00??????1???????????0??00??????????????????????????????????????????????????????????????????????????????????????????????????0????0??????????????????????????????????????????????????????????????????????????????????????????????????0011??????0????????????????????????????0?????0?0???00?????????0?00??0???????????????????????????????0?????????????0????????0????????????????????0010??????????0?????????????????00?0?1?

Epidexipteryx

?01???????????????????1??????00?????0??2??0011?????????????????100?0?0????00001020???10000???????????????0?[01]????1?1?123??10??0????0101?010?0??????00?1???????????022?????011?03?0??????????0????011?0??0?10?000???00??1?1?0???00?0?00???0?0?????????1??2?????0???????2?0?????0??????????0???????????????????????000?0????00??0?0??????????????0??0?0??????1???0?????????????00?[01]0????????????????????????????????????????????????????10?0?0?0????10?00???0?????????1??11??1????????????0?00?100?????0??????????0?????????0?????0?????????????????????????????????????????????????????????????0????0?????????????????????????????????????????00200??02?????????0???????0?1????????????????????????????0011??1???0?????00??????????01?????????0?????0?0?????????0????0?0????0????????0???????????????????0???????00??????0???0????0?????????0??????????001?00?????????????????????0???????????

Incisivosaurus_gauthieri

?00?00?01?001??1??100011101001001?010?120000110001010110101111210100001001?1?000210??00?0??????????????????????????????????????????????????????????????????????????????????????????????????????????????????????00??00??01?0??0?0?????????100000001000102?00010?100??????0000000000100001??0000???0000101?1100??0000???????????????????????????????????????????????????????????????????????????????????????????????????????????????????????????????????????01020?00???0??000?0?0????????????11?1100000????1000000?0?0000?000?0?000???1000??1??00?0????000?110??010??01?0000?00000???00?00????0100??0111000????????????????001000000010000????00200??02?1?000000000?????00110???????????????????????????????????????????????????????????????????????????????100?????????????0??0?001????????????????????????0201100????????????????????????????????????????0?0?0??????0??????????000?0????????

Citipati_osmolskae

?001001001001??221000101111?01011?000102100011000010001000110121120?0100010111?1?????????1011101100101211001??201??002200111?0111200110100100?11000001000211001??02012020??10111111?2101000000000?000000000000000000011020000000?0?001000?000002011???0???[01]000020001220001000000001102?1??0000??0000010001010??0000020011000[01]0?00?0010000101020??????0????111000?0?0010000?000000000000?00100?0??0?0???0000000????00000?2000000000000000000002?000?000000000020?000110??00[01]10?011011000000011?1100001????1000001?0?0001?11000?001???1002??0???0?0????000?000??000??01?0000?0000000000100?????100??0111000????????????????101000000010000????10201???2?1?0000000???0????????????????????????????????????????10???00?00???????????????????????????0?0?0???00100????000?00??0???0?00?????????0?0?0?00?0???0?00211??????????0??00?000?0???????0??????0?0000100?0100????10?????????1?00?0????0?0?

Oviraptor_philoceratops

?00?0??????01?1????0??1??111?1011???0???1?0?11???01?0???0?11?121120?01?00?01?1?1???????????????????????????????0????????????????12??1?????00???10??00?00??1??0????????????????????????????0????0????0??0?????00?00000???2?00?0????????????10000?0?0???????0??0???0????00???0?00????????10?0?00??0??00?00?1?1????00???00?100????0????????01010?0?00?0?0????1?100??????1100???0????????00?????????000?0??00000??????????????????????????????????????0??000?????20????1?0???02?0??1101100???0011?1?????1????10????1???0?01????????01???????????????0?????0???00??0?0??0??00?????00???????????????????0?????????????????????????????????????????1020????2?1?0000000????????????????????????????????????????1???1???0??????????????????????????????????????????100????000???????????0????????????????0??0???????211?????????00??????0??0??????????????????????00??0??????0???????????00??????0???

Microvenator_celer

??????????????????????????????????????????????????????????????21?20?0?????????????????????011?0?11000121100???0?1?1002????????????00?000?11000?????0010002???0000?????????????1110??0101100?0000010???????0??0???00??11?20?0??00???0?1??0?????????????????????0???0?2200000????????????????????????????????????????0?0001????????????????????20000?000?01?????00?0??010?00?000000000000010000?????00????????0???0??000002000000000000????????????10??0??????????????1????????0???????????00????????????????????????????????????????????????????????????????????????????????????????????????????????????????????????????????????????????????????????????????????????????????0???00??0001?01001?????????????010000?0?????100???????01?01??0000????00?????????????0?????0????????1????000?10?000???0010?0001????1??????00??00?10??00000000??000?0??00000??????????1?01??11100??001?????????0???

Caudipteryx_zoui

00010??????????????0?111??10?0001?10???21000???0?0????????????21120?0????????0?1??0?????00????0??00????1???0???01?????30?????0???0??0???01?0???100200?000?1??????0201202???1????101?11?????0????01000??1?00?0?000000?1102000??00?0?001000100000?00????0???00?00000?1[01]2000000000???????????0??????0000????1000???0000?00?0000?0?0?????????????20??0?0?000?11??00???????0?0???00?00????????????1??0000???0000000?000000000??0?0?00????0000000??0??2000000??0????????01101100??01?110???0??000?10???0000?????0????0?0?0001?00?????0????????????????0????00???00??0?0??0??0?????????000?0????????1????0?????????????????????????????????????????00201???2?????????0?????????0??0???????????????????????????1???????00??000???0000???????????????0?00??0?0???001?0?0???00?0???0????10?????0???000??0?00?0???0?0???11??0????00????0?000?0000???0?0?????0?0?00100???0????????????????1?10???0??0?0?

Ingenia_yanshani

?00?0????????????????1?1?????1????????????????????????????????21120?01000?0111?1???????????????????????????1???01????2?00???1011120011??00000??100000100021??????02012020011011?11101101?0000?00011?000000000??0000??11?2000?000?0?001000???????????????????0002?0??2200?1000000??1?????0?0??0??0000?00??1?10???000??0??1??0[01]0??02??????0101??0??0???0?0?0101000?????1100???00?0?????????????????00????0?00?00?000?0?000??0?0?000?0?0?0??00?0????00?000000????????0110??00?1?0?1101000??000?1????????????????????????????????????????????????????????????????????????????????????????????????????????????????????????????????????????????????????????????????????????????????????????????????????????0011000?001?0?01??101000??0101??11??0??????00000???00?????1?000000010??1?1?0??????10???0???0??0?000????11???0???010000000100000000??000100?000000010??????????0??1?0??00?1???1??0??0???

Rinchenia_mongoliensis

?00?0????0??????????0111?1???1?11?00010??00???0000?????00??1?12112??01000??111?1????????????????1??????????????0?????220?????????2??1???00?0???1??0001000?1?????1???????????????1?????????0????0??0?0???????0??00??001??2?00??????????????0??????00????????00??210??????2??0120????????10?0?10??0???0????????????0??????????????????????????????????????????????????????????????????????????????????????????00???0?00???????????????????????????????????????????????????002?0????????????00?1?1?00001????1??0001?0?0001?11??0??01???1???????????0????000?000??000??01?00???0?000000?0????????100??0????0????????????????????????????????0??????????????????????????????????????????????????????????????????????????????1?1001??????????????????????????????0???????????????????0????????????????0???????????11????????????????????0??????????????????????0??????????0????????????0??????????

Conchoraptor_gracilis

?0010??????????1???00111?1???1?11?000??21000110?00????1?0????121120?010?0?0111?1??????????01010110010??1?012?110??10????01?1?????20011000010???????0010002100010102002020??101101[01]1?11010000??00010000010?000??000?001102?0?0000?0?0010001000002001???????0?0?0200012??????000000000????????10???0100100????????000??0?010??????????????????????????????????????????????????????????????????????????????????00?00??000002000000000000?0000?002?01?????????0?020?000??0??002?00???????????0011?1?00001????1??00?1?0?0?01?11??0??01????0?2????????0????0?0??00??0?0??01?00???0?00?000?0?0??????100??0???00???????????????????10???0???????0????????????????????????????????????????????????????????????????????????0?0??????????????????????????????0?0????010?????010?????0?????0???????10???????00?0???????21?????????????????0?000000???????????????0?1?00????????00????????????0?0????0???

Chirostenotes_pergracilis

?????1??01?01101??0???1?110?0??????????????????????01010??????21120?00000201???1????????????????1101?12????1?12???0????????????????101?1??????????00?100021?00101022120201110?111[01]??01??100???00?100000200000??000?0?1??20?0?000?0?00100????????????????????000?????[12]????10????????001????00????????????01?10???0????0011??00????????????????20000?00000?????????????????????????????????????????????????00000?00??00000?00?0????????000000?0???0000?????001020?00???0????????1????????0?001???????????????????1?0?00???0??????????????????????????????????????????????????????????????????????????????????????????????????1000001000???????00200???2?1?000??0000??????????0???????0?0??00???1000?0??????????????0??0??101000?????1?0100?0??0?00??00000?001?0??1?1?000?01?????1?0??????10?????000??0??????02?1???0110?1???????000000000???00????00??00010?0??0?????1?0??0???????0?1???000??0

Avimimus_portentosus

?00?0???10011?00??0??1?1???????1???1?????00?11??00100110??????2?1???00?0??0111????????????011010110101?1?00??100????????????????????????0100??????????00?211?01??0201202010?011?0?1000100?0???00011110020???00??0?0?01?02?000000?0?001000????????0?????????00?01??0?02001????????????????????0??0???00??0??????????02001000??????????????????????????????0?????0?0?0010000?000000000????????????2???????????[02]0?000?0000020?010000000000000??00?01??0??????0????????????????????????????????1?0???????????????????????????????????????????????????????????????????????????????????????????????100??0?1100????????????????????0000001?0?00???????????????????????????????????0???????0001000001??????????????00001???????101001??0101?0100?0?000??001000??00???????0??0?1001??101????0000100000?????1001101102?????101010000000????00?000???001000000000010?0????1??01????0??00?1???1??????0??

Falcarius

??01?11100001111010?????1????00??????????00??????0000000??????100?000??0???????001010001?1??0001110101210100012000100??0000??????000001000100010?0000100?011001010220301010100100010000000000000110000000000000??000?10?00?0?000?0?001000????????0?001???00?0?0?0??11?0??000???????000??00???0??00?00100?0?00??0000??0010000[01]0??????????0100020000?00000001110?1?0?0010000?001000000000010000???0100???0000000?0000000002000000000000?00000000?0000??00000?0?200000??0???00000?1100?00000000???????????????????000?00?00100????0????????????????????????????????0?????0??????????????01?00????00?00????????????????????????0000001010010????0??00000001???0??????????????0?0???????0001000001000??1000??1010000000100000000000?0101?010000000001?0000001000?0??00101000010?1?01?11?1000100100000000011101002?0?101111000000101000100000??000001000?000000?0?10?10000?011011002?00?10?0000???

Beipiaosaurus

0??1???????????????????????????????????????????????????????????10?10?????????????1001001?100????1??????????????000??0220?????????0000??0????0?1000000?0000??001???20220??0??0????0??00?0??00??????0000????0???0??00111???0?0??0????0010?0??????????0??????????0???0??????0?????????????????????????????????????00????001000?????????????00?00????????????????????????????????10000???????????1??0100???000000???0??0??0?????0???????0000000000???0?0?00000????????????10?????1?1100??0??000?1?0?????0????????????????????0?????????????????????????????????????????????????????????????????????????????????????????????????????????????????????0??0?00?????????????????????0???????0??????????????????????1???0?00??00???00?1????????10?????0?0?????????00???????1?1?0????????1??1?????10???000?0?00???????????????????0??0?01000?01010???0?11??10??0?1?0???1???????????????????1????00?0?0?

Segnosaurus_galbinensis

??????????????????????????????????????????????????????????????21??10?0000?000???0100?001????????1??????????1?0?0??????????????????0?10?1?00000????0???20011100102020221??0110201101?11001?00?00?11000000021?0??00?01?1??0?0???00?0?010?00??????????0?????????00???????0?????????????????????????????????0?0????0??????????????????????????????0??????0????1????1???0010000?00100000?????????????????????????00?00000??10??????00?????000000000??21?0????00???????????????????2??????????0????????????????????????????????????????????????????????????????????????????????????????????????????????????????????????????????????????????????????????????????????????????????????????????????????10????????????0?00???????21020010?0101??02???????????0?00??????????????0??01??0??1??1????????2???????0??110????????1???????10100??1?11122210111111112?111201??????????0???1??????????????????1?

Erlikosaurus_andrewsi

?0012???2?0?1?1??1010011100??0001?1000001000010000000000??11112100100000020001?001001001?????????????????????????????????????????????????0?????????????????????????????????????????????????????????0000?021?0??00??11??00?0?00????????????0?00000010010??00000?000????0?000002000001???10?0100???0000?0?10000??0000????????????????????????????????????????????1?0?0010000?0010000000?????????????????????????????????????????????????00000000????????0000?0????000?????000?0?????????0????01?0001100????1?10000?0?00?0?100000000???0??0?00??00?0????000001000000??00?0200?00000101000?00001?000??00?0?00????????????????00000001101????0???01001000200000000000000??????0?????????????????????????????????0?00???????????????????????????????????????????0000???????00??0???0?011????????2??????????????11210?11???????1?0?1????????????????????????1?01001000????01??????????010?0??????1?

Alxasaurus_elesitaiensis

??????????????????????????????????????????????????????????????210?100????????????1001001??????????0?01010000?1?0101002?0?1???????????0?000??0?10?0000120?11??0??1?20?21???11?????????1????0?????????00000?10000??001?1??00?0??00?0?0???00??????????0??????????0?????0????0??????????????????????????????????0??00????0??00??[01]0????????????????0????????0??11??01??????0?0???010000???????????10?0100???000000???0??0?????????????????????????????1000000?0????????0?????????????????????000????????????????????????????????????????????????????????????????????????????????????????????????????????????????????????????????????????????????????0??0020?????????????????????0????????????11101?????1??0?11??0?00??010?0?????01?????????2????????????????200??0????1?1??0??0????1??1?100???????0000000??1??0???0?00???11?0????01100??11?1?????1111?????1?00???10?1???0????????????1???????0???

Neimongosaurus

??????????????????????????????????????????????????????????????21????0?????????????0??001?1??000?110101?1?0?10?2?1?1?02200????????0001010000??????????????111001?2?????????????????????0??00????0??000000021?00????0??1??00?0??00?0?????0???????????0???????????????1[12]200?0?????????????????????????????????????0?????00?0000?0??????????00000200?0?00000?0?11001?0?0?10?0???010000?0?00?????????????????????0???????????20?00???????0?00000000?0210??????0????????0??????????2?1100?000????????????????????????????????????????????????????????????????????????????????????????????????????????????????????????????????????????????????????????0????2??????????????????????0???????0??1??11???????0??01?10?0?00???????21?2????????????2?1???1?00??00000?1???0??00????00????00??????100010?2???????00?????1???0?0111?111001011????1?1121?????111111????10????1??11??0?0??0?100???1?1?????????

Erliansaurus

???????????????????????????????????????????????????????????????????????????????????????????????????10??1??????????1???????????????0?0???10?0?0???00000????1?0?1??????????????2??1????10?00?0?0001?0??00??????????00?????????????????????0?????????????????????0??????200?????????????????????????????????????????????0????0???????????????????????????????????01?0?0?10?0???010000???00010???????100???000000???????????20?00???????0????????????????0??????????????????????????????????000????????????????????????????????????????????????????????????????????????????????????????????????????????????????????????????????????????????????????????????????????????????????0???????????????????????????????0?0?1?0?01????????????????02?0???1?000??????????????00100???????0?????????00???100?????00?11????????????????011?111111??11?1???0?1?11111011?0?????????1??????????0?1??????00?0???

Suzhousaurus

??????????????????????????????????????????????????????????????????????????????????????????????????0101??01000??0??1????0??????????001010000???????????200111001?2020?2?10010020110101100?00??????????????????0???????1????????00???010????????????????????????0?????0?0?????????????????????????????????????????????20010000[01]????????????????20000?00000?01010?1?0?0010?0???0100000??00?????????????????????00?000?00?1020000?????????????????????0??????????????????????????2??????????????????????????????????????????????????????????????????????????????????????????????????????????????????????????????????????????????????????????????????????????????????????????????????????????10111100100000?11010?00??????021020010?0101?00201010????????????????????0???0??01??00?1????1000?0?2???????00????????????????11?100011????1112212111110??????????????????1?0??01102?0?2???????????1??

Nothronychus

?????1112??11?1?0?0????????????????????????????????00000????????????????????????????1????????????10101?10?01?1?00?100220?001?????00000?100?00????00000200??10?1?2020221100100201101?1100100?100011000000021?00???00??????0?0?000?0?010?00?????????????????????0????1[12]200???????????1?????????????????????????????????0010000[01]0??????????00000200?0?00000?0211001?0?0?10?0???010000???00010???????000???00???00?000000?1020??000000000?00000000?0210??0?000?0????000????????0???1100?000?000????????????????????????????????????????????????????????????????????????????????????????????????????????????????????????????????000?01100???????????????????????????????????????0???????0?0??1010110???0000?110?00001?0?0?021?20010?1101??02???1?10000?0?000?10?????0?1?0000010?0??1?11?1?0010020000?00001110?1?2??????1?1110?0001???111122210111111?12101120??0????1?1?0???10?????1???10????0???

Enigmosaurus

?????????????????????????????????????????????????????????????????????????????????????????????????????????????????????????????????????????????????????????111?01?2020221100??0201101??????????????????????????????????1????????00???010??????????????????????????????????????????????????????????????????????????????????????????????????????????????????????????????????????????????????????????????????????00?000000?10?????????????????????????????????????????????????????2???????????????????????????????????????????????????????????????????????????????????????????????????????????????????????????????????????????????????????????????????????????????????????????????????????????????1??1?????????????????????2?020?10?1101?????????????????????????????????0??01?????1??????????????????????????????????????????????????1?122121100????????????????????????????????????????????????

Nanshiungosaurus_brevispinus

?????????????????????????????????????????????????????????????????????????????????????????1???????101?121???0??????????????????????????????????????????????????????????????????????????????????????????????????????????????????????????????????????????????????0?????1???????????????????????????????????????????????200??0??????????????????????????????????????????????????????????????????????????????????0????????????????????????????????????????????????????????????????2?????????????????????????????????????????????????????????????????????????????????????????????????????????????????????????????????????????????????????????????????????????????????????????????0??????????1???1??0????????????????????????21?2??10???01????????????????????????????????????01??0??1????????????????????????????????????1??????????????11222??11?????????????????????????????????????????????????

Therizinosaurus

??????????????????????????????????????????????????????????????????????????????????????????????????????????????????????????????????0010?0000????0?00200?????????????????????????????????????0??0?11000000021?0????00???????????????????????????????????????????????????00??????????????????????????????????????????????????0??????????????????2000??00000?01?1001?0?0?10?0???010000?????010???????100???00000??????????????????????????????????????0??0100????????????????????????????????00??????????????????????????????????????????????????????????????????????????????????????????????????????????????????????????????????????????????????????????????????????????????????????????????????????????00?1010?00100101??????????????????????????????????????????001?0????????0??????????10??0000????????????????????????1110111111????????????????2?111?0?????????1???01????0????????????0???

Haplocheirus

?0010???00010?0?12011011101112001?000020100?0?1010??0001?0010000010110110?1000001101010100??10??0?0111201??0?00001?0???0?00???????100210001210?0?00010???001?00?101002010010?011000?00000000?0000?0000000??00?1110001100010001?0???0010?01000000000??0000001?00000?102000000000????00???00???0???000000??0??0??0000?000?000????0?????????????20000?000000010101??????100???000?00???????????0???00000??0000000?00?000100200000???????000?0??0???0100?000???0?0??????????00?0??????????0?0000001?00001??0?0000000?0?01000000?0??11???0??0?01??01?0????000001000000??00??100?00000000000?000????00??0??0?0??????001????????0?0??0?0000????0???00000??00?00000??00??????????000???????0??1???????????00?1010100??0100?00?0????????0?01??0???00?0?????0?????00000?00?101000??00?0??010?11001001000100000110??00??0?101???00000???0000?????????000????????00101???0?1?1?010?????0??0000??10000???

Alvarezsaurus_calvoi

???????????????????????????????????????????????????????????????????????????????????????????????01000???0???0?20?0?2012????????????000?00????????0??1?0000101?00?2??????????????????01?000??0??0??11000000??00????????1???0?0??00?0?????0??????????????????????0????10????????????????????????????????????????????????00100???0???????????????2?000?????0?0?0????????????????????????????????????????????????0??000?00???2????00000000?000???00?02???0?????????????0????????0??????????00???????????????????????????????????????????????????????????????????????????????????????????????????????????????????????????????????????????????????????????????????????????????????0???????0001?00????????00?10?0?0???????????000?000?????????????????0??00?????00????????????0??0??0????????1000???????10?????101???????1????00?????????00000???????1???0?000?10??????1???0?0??0??0????????????????

Patagonykus_puertai

???????????????????????????????????????????????????????????????????????????????????????????????1?01?112????012010?2?2??????????????00210???210?????1?0???1????1010?????20????21202??11000000000001210??0????001??0???????0?????????0????0?????????????????????0?????01?????????????????????????????????????????????0?00100???????????????????20000?000?00??????0?0?0000??0?0010000???0??????????1???????????0???0????100200000000000??00?0???????10??0???????????????????????????????????0??????????????????????????????????????????????????????????????????????????????????????????????????????????????????????????????????????????????????????????????????????????????????????????????00001?01100?0?????000?1100??????11???0?0?01?01?1?000?10??1????0?00?????0????00?000?0??1???0??1001?101??1?0?1111111????????????0?00000??0?????00??0000?00?0?00??1?????????10??1???0???0????1??0???0??

Achillesaurus

????????????????????????????????????????????????????????????????????????????????????????????????????????????020???2??????????????????????????????????????10??01?2???????????????????1??????0??1?01000??0???00????????1???????????0?????0??????????????????????0?????????????????????????????????????????????????????????????????????????????????????????????????????????????????????????????????????????????0???????????2????00000000?0??0??0??????????????????????????????????????????????????????????????????????????????????????????????????????????????????????????????????????????????????????????????????????????????????????????????????????????????????????????????????????????????????????????????????????????00?0??????????0????????00?0????0??????????????????????????????11????????????????11?????????????0????????????000???????1???0?00?????????????0?????0???????????????????

Mononykus_olecranus

??????00???????112???????????????????????????????????100????????????????????????2????00???????1?1?11102001???2?10?2??????1???1000?000200030210?002?1100??1????1?201000?2?????3??0??0210100111011212000030000001??00???1??0?0??00?0?0???00??????????????????????????00011???????????????????????????????????????????0?0010?0?????210???00?????2?010?0?0?0?010?011?0?0001000?001000000000??0000???30000???0???0??00???????2000000000000??0000??0?00?0??0??000???????01?0?????0?0????????11102????????????????????????????????????????????????????????????????????????????????????????????????????????????????????????????????????????????????????????????????????????????????0???????1001001000????????1010100000100?????011???0???01?010000010101?100000100????0??????00??1??0???021?1110010111111111101011???????0100000000000?000?0000??000010000?000?10????????100?10100?0?01???1??0??0?00

Shuvuuia_deserti

?00101002?010111120110001011?01111?10020100001010010010???1110?00101001002100??0211??000??0111111011102100?0?201012012110100?1000?00020003021??002?110000101001?201000?2002203??03?02101001110112120000300000011000001100000010000?00??001000000001?010??000000000?000100000000?101000?10000110?0111000??0[12]0????0100?0010000[01]0?0210?0?00?????20010?000?010101011?0?0001000?00100000?000?????????30000??000??00?000?00?1020000000000000?0000000?00100000?0000020?0001?01?1010001???????1110200?000?000??0?1??0000?0?00000?00000100???0??0?00??01?0????0?????0??000??00?0200?000000??0?0?0??00?000?001?0?0???????????????????000000000????????00000?000?0??000000??00??????0?0???????00010010001011?0001010100000100??0?01110000???01??1??????????0???0????00001????1??00??1??00110210?110010?1111111??0?01002?0??00??0?0000000?000000000??000?????00000010101?0??0?00110????00?1101?0????????

Parvicursor

????????????????????????????????????????????????????????????????????????????????????????????????????10?????????1??2????????????????????????????????????????1????2?1000???02203??03??2??100011?11211000030??00?????????1??????????0?00??0??????????????????????0?????0??????????????????????????????????????????????0?00?00???????????????????????????????????????????????????????????????????????????????????0????????1?2000000000000??000?000?00?0???????????????0???????????????????11?????????????????????????????????????????????????????????????????????????????????????????????????????????????????????????????????????????????????????????????????????????????????????????????????1000?????00?????????????????????????0???01??10??00?0?01??000??100???????????00??1????????1?111???????????11100010????????????0????????????0?????000??00000000010??????????0???1?0????1???1?????????

Albertonykus

???????????????????????????????????????????????????????????????????????????????????????????????????????????????????????????????????????????211?????1?0????????????????????????????????????????????????030?????????0???????????????????????????????????????????????????????????????????????????????????????????????????????????????????????????????????????????????????????????????????????????????????????????????????????????????????????????????????????????????????????????????????0??02????????????????????????????????????????????????????????????????????????????????????????????????????????????????????????????????????????????????????????????????????????????????????????????????????????????????????1??????????????????????????????0???00????0??????????????????????????????????11???11??1???????????????????????????????????????????0????0???????????1??????????????????????????

Albinykus

??????????????????????????????????????????????????????????????????????????????????????????????????????????????????????????????????????????????????????????0????????????????????????????????1101??121100300000?????????????????0000?????0?????????????????????????????????????????????????????????????????????????????????????????????????????????????????????????????????????????????????????????????????????0??????0????????000000000?0000000?00??0????00????????0???????????????????11??????????????????????????????????????????????????????????????????????????????????????????????????????????????????????????????????????????????????????????????????????????????????????????????????????????????????????????????????????????????????????0?010000??00???????????00??1???????????????????????????????????????????????????????????????????????00000?10??????????0??????????1?????????????

Bonapartenykus

????????????????????????????????????????????????????????????????????????????????????????????????????112??0????????????????????????000210???????????????????????01????????????212?2??1????????????????????????0???????????0?0???????0????????????????????????????????0????????????????????????????????????????????????00100???????????????????20000?000?000??10???????????????????????????????????????????????????????100??????????????????????????0?????????????????????????????????????????????????????????????????????????????????????????????????????????????????????????????????????????????????????????????????????????????????????????????????????????????????????????????????0?1000??1?????0?010?010??????????????????0?0?01?????????????????????00?????00???????????0??????1???011?????????????????????????????0??????????????????0?????0????????????????????10????0????????????????

Ceratonykus

?0010???2??10?????????????????11???10?201?0?0?0100??0?????????????01?0??????????????????????????1?1??????????????????????????10????0?2???0?????????????????????????????????????????????1????????212?0003???00????????????????????0?????0???????????????????0???????????????????????00???????1????111????????????????????????????21?????0??????0000?0???01???????????????????0????????????????????????????????????????????????00000000??000?000?00?0????????????????1????10?0???????????????0??0?????????????????????????????????0?????????????1?0????0?????0??000??0??02???0?0000??0?0?0??00?000??0????0???????????????????0??0?00??????????0?0????????????????????????????0???????0001?????????????????????????????????????????????????????0?0??1000???????0??????????????????1?????1?00?0?????????????1???????????????????????????????????????00?00001???????????01???????????????????????

Linhenykus

????????????????????????????????????????????????????????????????????????????????????????????111?101?1??1?0???2010020?2???????10?0???1??????????0?24110??????????2??????????????????0?1010001????2?2?0?030?0000???0????1??????????0?????0???????????????????????????01??????????????????????????????????????????????0?0012?0?????210????0?????20??0???0?0?0???????????????????10000???0??????????31??????00??0?????????1?2000000000000??000?000?001?????0?0?????????1???????0?????????????02????????????????????????????????????????????????????????????????????????????????????????????????????????????????????????????????????????????????????????????????????????????????0???????0001?01000?????0??????1??????????????????????????0?0??00?0?0???000???00?0??????1??0????????????1011????????1111?1???0??????????????0????????00???????????01??00?00001???????????0?1?110?0?????????0??0???

Xixianykus

????????????????????????????????????????????????????????????????????????????????????????????????????102?00?21201??0???????????????????????????????????0?01?1??1?200000?20022?3??03?02?010011101121211??3???000???????11???????00?0?00??0??????????????????????0????????????????????????????????????????????????????0?0010000????????????????????????????????????????????????????????????????????????????????00?000000?1?2000000000000??000?000?0?????????????????????????????0???????????????????????????????????????????????????????????????????????????????????????????????????????????????????????????????????????????????????????????????????????????????????????????????????????????1000?0??????????????????????000110000???01?010??00?0?00010?0??????????????????????0??1???1011????????????111??11?????????????0??????????0?0000??000010000000001??????????0????100????1???1?????????

Nqwebasaurus

?0????????????????02????2010????????0100?00??1?????0?00????????????????????????02????1??????00?11001???1??????????????????????????0102?000??0?00?10110??????????????????????????00?????0?0000100010000000?000?1??00??????0?0?0?????0????01?00???00?????????????????10200?????????????????????????????00????????????????????????0?????????????20????0?0???0101?0??????10?0???????????????????????00000??00000??????????00????????????00?0????00??0?0??00000????????0??????0?0??????????0?001?????????????0?0??????0???0??????????0???0??????0??????????????????????????1??????????????????????000??0??0?0??????????????????????00???????????????????????????????????????????0?????????????????????????000??01?0?1?0?0????????????????????????0????00?0???00?????0?111?0???1??0??012?????100??00010000100001???????0?????????????0?????????????????0?000010??????????0???????????1?1???00?0???

Shenzhousaurus_orientalis

???0???????????????21000??10?0??1???000??00?000?0???????????0?00000000??0??0?1?12?0??1????????????000??1???0???00000???0??????????????????????????010000000??11000100101?000001100??00001?0??????????????????0??1??1?0011?????00???0010??10000010????????00??0?00???0???00?000????????????????????00?????0??????0?00?0?100?0?0?0?????????????????????????????????????????????????????????????????0?????0?00000?00000?0002?000?????????????????????????00?????????????????00000???????????01?00??00000??0?0000?00?0?0000000000??00???0??0??1???1?????????????????????????????????????????????????????????????????10???????0??????????????????00?00??00?00?0????0?????????????????????????000???????00?????????????????010100000?0001?00??0?????????????????01?????0?10??00??1??00?2?0000????????100?0????????00?10????00????????00000000???00?????????????0???0??0???1???????????1???0???0???

Ornithomimus_edmonticus

?00010?1101101?101021000?0101010110000000000000001000000??????00000000010?1001?1???????0?001?1011000011100010100000000100100??????01120012000000?20200100000011000110101100000110010000011000100010000020??00?011111110121110000?0?0010001000000001?????0001?000000002100000000?????????0?0?10???000010110??????000000010000[01]0?0??????????????0??0?0?0?0?01?1001?0?00?000???0000000??00??????1??00000??0000020?000?000002000000000000?0??0?????0??0??000?1???????0???0??0000000????????00010000?00000?00?0000?01?0?0100000??0??00???0??0001??01?0????000001000000??01?00????0000101?0000??000000??0????0????????1??????????1????????????????00210???0?00000?010????????????010?00?00001000011???????????????????10?00???1?????????????????????????1???????0111100111111111?11000???????100??0???00?01????101?0???1??0?0000?10000000000????0000000000000100?0????????1???????????10?01???0???

Archaeornithomimus_asiati

??????????????????????????????????????????????????????????????????????????????????????????00?101100001110001?100000000?0??????????010200?20000?0010[12]0???000?01100011010110000010201000001100??000?0000020??0000?10??10??1?1??00??0?001000??????????????????????????????????????????????????????????????????????????0?001000?[01]0???????????????20?00?00000?0?0??01?0?0010000?000000000000010000???00000??000??00???00000002000000000000?0000??00?0?10?000?????????????0??????0?0????????00001????????????????????????????????????????????????????????????????????????????????????????????????????????????????????????????????????????????????????????????????????????????????0???????0??1?00001?00??00000?00020010?010?010100000?01011?00000000000?000001000?????001?101?001?11?0????000010000001100001??011???????110?000001100000000000??000000000?000?10??????101?0?1?000?000????1?????????

Anserimimus_planinychus

??????????????????????????????????????????????????????????????????????????????????????????????????????1?????????????????0??????????1?200?2????00?20200100000011000110101????001?001????????????????00002???00????11??10??1?1??00?0?001000?????????????????????????????????????????????????????????????????????????????????????????????????????0????????0??1?????????????????????????????????????01000??00000?????????????????????????10000??00??0?????00?1??????????????????????????????001?????????????????????????????????????????????????????????????????????????????????????????????????????????????????????????????????????????????????????????????????????????????????????????????????????????????????????10?0??1?1?????????????????????????1?0??????????11011011001??1?0????????100???01?00?????????????????????000?10?000?????????????????????010????????????????????????????0000???

Struthiomimus_altus

?00010?110??0??1010210002011101?1100000000000000010??0001?01??0001000001020001?1?????????001?1011000011100010100000000100100??????011200120000000201001000000110001101011000001?0010000011000100010000020??000011111110121110000?0?0010001000000001??????00?1?0000??2210????0???????????0?0?1????000?????0?00???00?0000?0000[01]0?0?????????????200?0?0?000?0101001?0?0010000??00000000000?100000??00000??0000000?000000000200?00000000000000??00?0210?0000?10022000000?0??0?00?00?????????0010000000000?00?0000?01?0?01000000000000???0??0?01??01?0????0000010?0000??01?0000??0000101?00?0??000000??00?0?0???????????????????1000011010?00????00210???0?000000000????????????00??00?00001000011?????000000000200?110101?10100100?01011?00000000000001000100000111001111111?1?110000??000010000001100001000110100???1??0?00001100000000000???000?000000000100?0?0?101?1110000?0000?1010???00???

Gallimimus_bullatus

?00010?110110101010210002011?0101100000000000000010000001?01000000000001020001?1?????????00111011000011100010100000000100100??????0112001200000??20100100000011000110101100000110010000011000100010000020??000001111110121110000?0?0010001000000001?????000110000000221000?00000001000000?0000??00000?0?10200??1000000010000[01]0?0?????????????20010?0000000101001?0?0010000?0000000000000100001??00000??0000020?000000000200000000000000000?000?0210?0000?1002??0000?00??0000000???????000010000000000??0?0000?01?0?01000000000000???0??0?01??01?0????000001000000??01?0000?00000101000?000000000?000?0?000???????????????0010000111100000???00210?0?000000000000000????????000000?00001000011[12]000000000000020011?0101010100100?0101100000000000000100?10001110111111011001?110001??000010000001100001000110100???1100000001100000000000??0000000000000010000?001010111000010000?1010?00000?0

Garudimimus_brevipes

?000?????01101????02?00020101000??00000000000000000?00001?0100000000000002?001?1???????0??0111????0001?10001?100??00?????10????????????????????????????0000001100????????????0??001000001?000?000?000001000000?01??1110?2?1?0?00?0?0??000100000000??????0000100000010???00?0000?001000000?0000??0000010110000??0000000010000???0????????????????????????????????????????????????????????????????????????????00?0000000002000000000000000000000?021?0????0000????00???0??001000????????00???0000000000002?0000?00?0?01000000000000???0??0?01??01?0????000001000000??01?0000??00000000000000000000??00?0?000??00?010???????001000000010???0???00210???000000000000000????????0?0?00?0000??0001110000000????????????????010100100?0101?0000000?000000100?10001111??????001000?1?0001??0000???????????000010110100??????000??????????000000??0000000000000010000?0010??01??0011??00?111?0?0???00

Pelecanimimus_polydon

?00???????1????????2100?2?1??0000??000000?0000??????????????0?000?0000???????000211??0001??000???0?0?111?00???????????????0??0?00???120????00??0?2010????????????????????????????????????????????????????????1001010????0?????0?????0????10000??0?1?0????00????0000?0?10???0?0??????????0?0?????????????????????00???0?10??????0??????????????0??0?0?0???0???????????????????????????????????1??00000??00000??????????????????????????????????????0??000???????????1?????0?00???????????001?00??00010??0?0000?00?000000000000??00???0??0?01??01?00?0?0000011??000??????????????????????????00??0?000??????????0010000????0??????????????????0??00??00?????????????????00000000?10???????0?0??????????????????????0?01?????????????????????????????????????010?1??111??????0????0?2?0?????000001100??????????0?1101??????????0?000????????????????????????1???0??????1??????????111??10000???

Harpymimus_okladnikovi

?0?????????????????2100??????0??????000000000000??????????????000000?0000????1?1200??1???0???1???0?00??10001?100000?00?001????????0?1?001200000000010?10??00???????1????????????00?00??????0??0?010000010??000001011????1?0???00?0?0???001??0000??1?0????00??0000??11?10000000????????????0??????????????0000??00?00000?0000[01]0????????????????????????????10??01?0?0010000?000000000000???0?010?00000??0000000?0?0000?00?????00000000?00000?00??11??0000?0????????0???????000?????????0?001?000000000????0????0??0?0?????0000?000???0??0?01??0??????????????????0???1?00??????0????????????00000??00?0??????????????????????????????????????00210??000????????0????????????0???????000??0000?1??0????0000??2001?00?01?1??0010?????????0??????????00?0??0001100100011??0??0?11?00????000???000001000????011???0??01???00000110?000000000???0?00???0?000?100?01??1?1?01??00??00???1????0000???

Beishanlong

??????????????????????????????????????????????????????????????????????????????????????????????????????????????????????????????????010200120000????0100?????????????10???1??00??????000001100?10001000?01??000?0???1???0??1?1????????01000????????????????????????????210??????????????????????????????????????????????????0??0???????????????20010?0000000101001?0?0010?00?000000000000010???????????????????0??????????2000000000000?00000?00????0??0?0?0????????0?0?????????????????00?01??????????????????????????????????????????????????????????????????????????????????????????????????????????????????????????????????????????????????????????????????????????????????????????????????????????00000020011?????????????????????00?????0?00000?0???00?????00??1?0???0??1??????????1000000?1??00?0?011?????????????000100??00????????????00000000001?????????1???10?0??0??0???1?????0???

Sinornithomimus

?0001?????1?0????1?21000??1010001?000000000000000000?000??0???00000000?00????1?1?????????0001?0?10?0???100?1?1??0?00?0?0?100??????010200120000?00101001000000110001101011000001?00?00000110001000?000?010??000001011100121?1??00?0?0010?01000000001?????00001000000?02100000000?????????????00??000001?1?0??????00000001?000?0?0?????????????20010?00?00?0101001?0?0010000?0000000000??01000010?00000??0000000?000000000200000000000000000??00??110??0000?????????000???000000????????0?0010000000100?00?0000?01?000100000??0?000???0??0?01??01?0????000??10??000??01?0000??000??????00000000000??00?0?0?0?????????????????10000????????????00210???0?000000000????????????0???00?00001?000?????0?0??000000200?100?01010100100?0?010000?000?00?0??1?0?1?00111?1001110?1001?11?00???00001000000110000100?11??00???110000000110?000000000???00000000000001000??0?101?011?000?0?00?101??00?0???

Qiupalong

?????????????????????????????????????????????????????????????????????????????????????????????????????????????????????????????????????????????????????????00??1100????????????01100?????????0??0001000002???00????????1????????00?0?0???0????????????????????????????????????????????????????????????????????????????????0???????????????????????????????????????????????????????????????????????????????????20?0?0?00000?????00000000?0000?000?02????????1????????0??????????0????????00??????????????????????????????????????????????????????????????????????????????????????????????????????????????????????????????????????????????????????????????????????????????????????????????????????????????????????????????10100?00?0001??????????000?010001??0??????????1??110????0?????????????????????1??01??????????????????????????0000??000??0000?00001??????????01??????1???????1?????????

Kinnareemimus

???????????????????????????????????????????????????????????????????????????????????????????????????????????????????????????????????????????????????????????????????????????????????????????????0???00001???00????????????????????0?????0?????????????????????????????????????????????????????????????????????????????????????0??????????????????????????????????????????????????????????????????????????????0?????????0??????0000000000000?000?02?????????????????????????????????????00?????????????????????????????????????????????????????????????????????????????????????????????????????????????????????????????????????????????????????????????????????????????????????????????????????????????????????????????????????0???????????????000??00000?00??????????????????????????????????????????00???1????????????????????????????????0???0000???001???????????0??????????????1?????????

Huaxiagnathus_orientalis

?00?0??????????????0001???1???0?????00??0?????????????????????00?0010????????00010?1010?00??0???1????????0??????00???0?002010????000000000?00??000000000020?0??000100201?000012?0???0??????0??0?010000000?0?011??000????00?0??00?0?0010?0100000?0?1?00????0????0000??200???0000??????????????????????????????????00?00?1??0?[01]0?0????????0000020?00?0?0?0?010100??????10?0???0???????????????????010????000??00?00??00000??????000?0??00??0??0????100000000????????000????00000?0?00??000000?01??00000??0?00?0?00?10000000000???1??????????????????????????00?00???????????????????????????????????0?????????????????????????????????????????02?00???0?????????????????1?0?00???????0?0??0000??????0??0001100???1?0?000??00000??0????????????0?????0?0???0?0?0?00?101000??01?0?00?0?00001000?000??0???????0?????10?????00????0?0?0?0000????0???0????0000100?????????????????????000???00?0?0?

Sinosauropteryx_prima

000?0??????????????0001???1???0?????0000??????00??0???????????00??01???????1?0001001010?000?01??100????1?0?0????00?110000201??????00000000?10??000000?00020??????0100201?000?11?0?1?00?0??000?0?01000000000?011??0000??000?0??00?0?000??010000000?1?00?00?????000?0??20000?000??????????????0???????0101????????0000?0??0?00[01]0?0??????????????0?00?0?00??010?000?????10?0???00?00????????????1??0100???0000000?00000??0?2?????00??0??00??00?0???0100000000????????000?10???000?????????0000?01???0000??1??1????0?1?0?00??0??????0?????????????????????????00??0???????????????????????????????????0?????????????????????????????????????????02?00???0?????????????????000100???????0?0??0010??????0??00111?0???110?000??00000??0?0???0??0???0??0?00?0???0?000?00?11100???0000?00?0?0?001000?0?0?0??????010????1100???0000???0?000?00000??000???????0000100?????100?????????????000???0000?0?

Compsognathus_longipes

?00?0???????????????00101?1??00???00000????000????000???????0?00000100?1?001000010010101000?01??1010???1?0?0???000?0120002010?????0?000000?10????0?000000??????0?01002010000012?0??????????00?0001000000000001100?00????0?0???00?0?0010001100000001?00000000?0?000??020000?0000??0?0??0??????0???0000????01?0??0000000010000[01]0?0????????00?0?20000?0?0?0?010100????0?1000???0???00??????????????010????0?0??00??0?000?00??????00??0?0000?00000?00100000000????????00????000000????????00000?001?001000?1?0100?0001000000000000??0???0??0?000?00?0????0000000?00?0??01?12????????000???????11?000?????????????????????????0??????????????????02000?00000020000?0???????1?0000???????000??0000?0????0???????01???1????00????0??0?0?0???0??????0??0000?0???0?000000?101000??00?0??0?000?0010???0?0????0????10????1100??0000????0???0?00??????00???????0000100?0010100??1?0????????001???00?0?0?

Juravenator_starki

0000???????????????00010??11000?1???00000?0000?0?????00?????0?0000010???0????000100101??00??0???1???????0???????00??100002010????000000000000????0000000000?01100???????????????????0?????00??????0?0??000000?10?000?0??0??0??00?0??????010000000?1?00?0?000??0000???20000?000??????????????0????????????0??????000?00???00??0?0????????[01]00002???0???????0101000?0?0010000??000?000?000???0?0????100???0000?0???00?000??2??00??????000??0?0000?00100000000????????00???00?0?00???00??0?0000?011000?00?01?0100?00?1?00000000000?00???0??0?00??00?0????0000000??000??01?10????000??00???????11?000??00?0????????00?????????0??????????????????02000???0?????0???0???????0?010??????????????????????????0000??00??1?0?000??????0????????0??????0?????0?0???00000?0??101?00??00?0??0?0???0????0?0?0??0?0?????0????0?0?????000???0?000?0???????0??????????00100?0?????00?1??????0???00????00?0?0?

Sinocalliopteryx

0000???????????????0001??0???000???00?000?000??0??????????0?0?000001???????1?00010010101000?01??10?????1?0??????00??1000020?0?????0000000??00??0000000003?0?0??000110201?000012?0?????????00???0??000??0000?011??000?1??0000??00????010?01?000000???00?0000???00000??20000?0000?00?????0????00?????0????????????00000001000?[01]0?0????????00?0?20000?0?000?010100??????10?0????0??00???????????1??010????0000000?0000000002??00?0????00?0??00?00??0100000000????????00??10?00000???00??00?000?01???0?010?0?0000?000??0000000000???0???0??0?00?????0????0????00?00?0??0???2?????00???????????11?0????0????????????0?????????0???????????????????2?00??00?00????????0?????1???0000??0??000?00010??????0??000010????1?0?000??00000??0?0???0??????0??0??0?0???0?000?00?100000??01?0?00?0?0?0010?0?0?0????0?0???0??0?0?00??0??0???00?000?0000????00????????000101?????10???1??????????000???00?0?0?

Mirischia

?????????????????????????????????????????????????????????????????????????????????????????????????????????0??0?0???????????????????????????????????????0????????0001?02000???012100100010110????0?????????????1?????????????????????0010??????????????????????????????????????????????????????????????????????????????0?1?0??????????????????????????????????????????????????????????????????????????????????0???00?0?000?????????????????????????????????????????????????????????????????????????????????????????????????????????????????????????????????????????????????????????????????????????????????????????????????????????????????????????????????????????????????????????????????0???000??????????????????????1??????0?0?00?000?1??0?0??????????????????????0??????0??0????0????0???????????1????????????????????????????000?0????00??00??????????????????0??????????0????1??????0??

Ornitholestes_hermanni

?0002???0?0?00?1???0?010?01110001?100101?0??1000001?01011????0000001000?0?000010?00101001?????011?0101210000??000?0010??1???????????????01?00?????????00001??1?0?0100101000001??0?1???????0????????00?00????00000?0010?00?0?0000?0?0000?01000000001?01?00001000002?112000000010?0??000000?0?00???0000?0?0000???0000000010000[01]0?????????????????????????????????0?0?0010000?000000000000??0??0????1000??0000000?000?00000?0000?????????0000?000?001??00?0????????00??0???0001?0?????????00??00?1?0000000000000000?000000000??00??0???0000?000?00?0????0000000?0000??01?0000?00?0000100??00001?000?0001100?00000?01000?????000000000000???0???02000000000020000?00000?00000000???????0001000002?10?00?0??????10010?01??000000000???0100????000????0?00?0000?0000???????????001??1010?0000???0???0????0?00??001000000?00???000000???000000??000?????????0?1000?1001?0001????0??0?1010?01???????

Coelurus_fragilis

?????????????????????????????????????????????????????????????????????????????????????????0??010010000121000?????0?0002????????????0?0??0?10001????0??????????????????????????01100?000001?0?0000110?0?0????0?00??00???0??????????0?0????0??????????????????????????11200?????????????????????????????????????????????001000??????????????????2?????????????01000?0?0010000?000000000000010000???0????????0000?????????002000000000000??000???????????0000???????????0??????0???????????0???????????????????????????????????????????????????????????????????????????????????????????????????????????????????????????????????????????????????????????????????????????????????0???????0000100002?????0?000?0??100100????????????0?0001?000000000?0000?????0????????????1??00??????????0000??000???0??00?0100????????110000000000?00?????????00000??00000001?????????0?1?0?11221111?????????????

Tanycolagreus

?000????????????????00?0??????0???00110????????0?00???????????????????????10?00??????1?????????????0011?000?????0????0?0??0???????000000000011?1000000????????????????????????110010000011000000010000000000000??00???0??000?????0?0???00??????????????????1???1????[01]2000???0???????????????????00??0?0??????????0?00001000??0?0?????????????20000?0000000101000?0?0010000?0000000000000100000??01000??00000??????????002000000000000000000000?00100?00000????????0?0?????????????????00000?????001000???????????????????0??????021000000?000?0?????????????????0??0??00???????0?0?000??00???????????????????????????????????????????????????????????????????????0??????????????????????00001????????0011001001000000??????????00???000000000000000000?000?????00100000000??0??0?0?100?1000000000000101000?????????????0000000000?????????0?0000000000010?????0??0?100101221011???1??0??1???

Tugulusaurus

????????????????????????????????????????????????????????????????????????????????????????????????????????????????0??????????????????????????????????0?0?????????????????????????????000?0?100??00110?0?1????0?????0??????????????????????????????????????????????????????????????????????????????????????????????????????????????????????????????????????????????????????????????????????????????????????????????????????2000000000000????????????????????????????????????????????????????01??????????????????????????????????????????????????????????????????????????????????????????????????????????????????????????????????????????????????????????????????????????????????????????????????????????????????????00?????????????????000000000000?0???????0?????????0???????????????????????????000001???1????????????????????????????????????0?????00??????????????????0001??1????1??0??????

Zuolong

?0000??????????????0000010111?0???000?0000001100000?????1000?????????????????00010?101??1?010001000????1???000000?0000??????????????????000?0??????0?????00?0100?????????????01100110000110?00?0???00010?0?00?????0?0100??????00???0???0011000000??0???00??1???0??0002000??0000?????????????00??0000000??????????010?0?10??0?0????????????????????????????????0??????10000?000000000000010000???????????????00?0?0?00?0020000?????????0000?000?0?1???0??????????????0???000100????????0??00?????0000000000000?00000001000???????1???0??00?0000??????????????????0?????0000?00000?0?000??10??0010?0001101?00000????????????????????????????????????????????????0??????????100???????00000??0???000???0??????00000?????0?00000?0?0?0??0000000000????000?000?00??0????0?????000??00?0???0???????????000011?????0?0??0000?0?00000??00???0?0???0000000????00??0?????1?0?10??000??01?0?01?????????

Bicentenaria

???????????00?1??????0?0??????00??00?????????????00????????????????????????0?000000001????????????100?????????0?????????????????????0?00???????????0?1????0??10????????????????0???00000?10???0?110???????0??????????1????0?????????????????00??0???0???????????????1???0??????????00????????0??????00???????????0???0010????0???????????????2???0???0???0?????0?0?001??????00000000?0??????????????????????0??0???0????2000?00000000?????????????0?????????????????????0????????????????00????????0???????????0???00??0???????????????????????????????0?0000000???????????????00000000?1?????????????????????????????????????????????????????????????1?2?00??000??0??????0???????????????????????1?00??0????00????????0??0?????????0000?02???00?0???????00????????0?????00??????0???00???0???????00???00?????0??????00??00?0??????0??0?????00?????00???????????0?????0?0??0?1?0????????????

Kileskus

???????????????????0?01120111????????????????????????????????????????????????00000?101?????????????????????????????????????????????????????????????????????????????????????????????????????????????00?00??0??????????????????????????????1?0000?0???0??00??????1????????0??001??????????????????????????????????????????????????????????????????????????????????????????????????????????????????????????????????????????????????????????????????????????????????????????????????????????????????1011201???000?0000?0000?0?????????????????????????????????????????????????????????????????????????????????????????????????????????????????????????????100010?????????????00???????????????????????????????????????????????????????????????????????00?????00???????????????1??????0????????????????????????????0??????????????????????????????????????????1?????????????????????0????????????

Guanlong

?00020?000000?010[01]100011201112000?001100000010000000000?10????00000010?00??200000001010?100?000000000111000000000000?0???00???????000000100000?0000000003000010000100200000001110010000011000?0011000010000000000000000000000000?0?0010001100000001100?00?11101102?1120000?0010????0000?000?00??0000000??0?0????000000010000?0???????????????20000?0?????0101?00?0?0010000?000000000000010000???01000??0000000?0000000002000000000000000000000?00100?0000020?20?000?00??0001000???????000000000000112010?1000?00?1000001010000?003??0000??0??00?10001000001100010??01?1000?00000000000000?1000011?000000?00000?????????????020?01000100000000000????0?000000010???????010100???????000010000210000?0?0000010001000000000000001000000?0110000000100000?0000000?0001100000100?010010?1000100000000000010000000000?00100100000000000000000??0000000000000010100???1?0011?1001??1000001??00?0?0?

Sinotyrannus

???????????????????0?01???101?????????????????????????????????00??0?1????????0?0000101??????????????0??????????????????????????????????????????????0??00??0?0?000????????????????????????????????????????????????????0??0?????00?????????1?0000?0??100??0?????11????????00?001?????????????????????????????0????????????????????????????????????????????????????????????????????????????????????????????????00?000?0?0???????????????????????????????????????????????????????0???????????00??????01?2?1??10???0??1?00????1?????????????????????????????????????????????????????????????????????????????????????????????????????????????????????0??????????????????????????0??????????????????????????????????????????????0001?????????????????????????????0?0??????0?????????????0?1????????????????????????????0??????????????????????????????????????????????????????????????00???????????

Proceratosaurus_bradleyi

?00?2??????????????00011?01112000?00??????????00?00???????000?00000010?00?120000000101011??????????????????????????????????????????????????????????????????????????????????????????????????????????????????????00??0????0?0??0???????????110000?0?1100?00?11?0?1?2??????00000100000?????????00???0??00??10?0????00??????????????????????????????????????????????????????????????????????????????????????????????????????????????????????????????????????????????0???????0??????????????????00?0010112?10?1000?00?1?0000101???0????????????????0?1000100000110001???????0???0???00000000??0?0????????????0000??000?????0??0?0??????00100?????0001???10?00000001000?????11000???????????????????????????????????????????????????????????????????????????????0?00????????????1????0?0????????????????????????0??0010????????????????????????????????????????1?0?1?????????????????000??????????

Dilong_paradoxus

?0002??000000???0?100011??1112000?00110?000010000000100???????00000000?00??200000001010?1?0000?0001?0??1000?????0??0?0?0??0???????0000001100?????0000000?00??1??00110201?0?0001?00?000?0??00??00010000100?0000?00000?0?00000?000?0?0010001000000001100?001111?110201?20?000000????00000???0?0???0000010110000???000??00??00????0?????????????20000?0?0?0?0101000?0?0010000?0000000000000?0??0????1000??0000000???0?00000200000000000000000??00?0?1000?0000?0?20?00???010000100????????000000000010002?11?1000100?0?000000000000103??0100?00??00010111100001100010??00?1100??000000000010????00000?000100???????????????????020?010?010?0????01001??10?10002?00?11?????01110000000100000100001???????00000110001????10000?0001??000???011000000000000??010000000??11000????1?01?01001?????00??00???00???00000?00100?00?0000010?0?000000????00??0?000?0001?100???1??10112????0??0000??????????

Eotyrannus_lengi

????????????????????000120??12??0???110???????????0?????????0?000?0?0??1???20000000101012?00?????01?0??1??????????????????????????000000010????????0?0?????????????????????????????????????????0???00000?0?00???00?0????00?0?????0?????0?1?000?00??10??0121???111?0?211?0000?0???????????????0??00?00100???00??0???0?0010?0??0???????????????200?0?0?000?0?0?0?0?0?0010000?0000000?0000???????????00???????????????0??????????????????????????????0????????????????????????1???????????0?00?????1?00101???????00?0?01????01000?1010??1??0?00?00????????????????0?????????????????????01?1??0??????????????????001??????0?00????????????????????000?1002110100??????????2??00???00???00????0??????????00??011011?000???????????????????????????01??00?00100?000?0???0??????1?0??0?00????1000??????0?00????????0000?00???0000100?00??0??????????0000???0?1????0??????011??????1???0?1?10??0???

Juratyrant

??????????????????????????????????????????????????????????????????????????????????????????0?00?0000001?10??00000??0???????????????????????????????????00?00?01000011010110000011?01???0??10????0?????????????????????1????????00???0010???????????????????????2????11??????????????????????????????????????????????0?00100?0?0??????????????????????????????????????????????????????????????????????????????00?000000000?0?00?000??00????????????1?????????????????????????1?0?????????????????????????????????????????????????????????????????????????????????????????????????????????????????????????????????????????????????????????????????????????????????????????????0????????00?100011?00100?0????????????????100000011001000?????1100001????????????????????0??01??1??1?????000????????????0?????????????0000?0??????????000000??000??0000?????????????1??1??????11??0????1?????????

Xiongguanlong

?0002???00000?????1?000220???0000?001101000?21000000?0001??000???????????????00000?101??2000000000000111000????????????????????????????????????????????0?12?01000???????????????????0000110??????????????????0??????0?00?????000?????????1?000000???00?0?1121?2102011???0??0000?00?000??????00???0??000???????????00?00100??????????????????????????????????????????????????????????????????????????????????00?0000000??20000???????0???????????????????????????0???????000100?????????????0010?10001????1?00?01?0?01?0000100??0010?0100??0??0??0?????0???01000?0???0?0100???001?111110010????100?1?11??00?100????????????001001000010?1???????????????????????????????2?000??000000011000011????????????????????????111??001???????0111012???????????????00??0???????????1??1?010?10?????????????00??????000?0??00001???????????000000?????000000???????10????1?????????1???0?0?01?????????

Dryptosaurus

????????????????????????20??????????????????????????????????????0?0?0??2???????0000101?1????????????????????????0???00???????????????????10???????40?0????????????[01]?0?0?2???????101?01000100010001000??2???0??????????0?0????????0?0???????????????00????????????????11????0???????????????????????????????????0?????????????0?????????????????????????????????0?0?0010000?00????????????????????????????????0????????002000000000000??000??0?????????????????????????????????????????00?01????????????????????????0??????????????????????????????????????????????????????????????????????????????????????????????????????????????????????????????????212?1?1??????????????????????????????????????????????10?????????????????????1?0???012?1?0111?????1????0??????0?????????????0????????????????000000????????????????00?1???00?????????00??0000?00001????01??????????0?1??00?0?0?????0???

Appalachiosaurus

????????????????????0?0?201110??????120?????????????????????00000?000?0????????00001010?2???????????????????????0?0?00???????????????????????????????????????????000010120?10???10??01000100010001000002?0?00??????0????0????????0?00100?11000000?1???1?021??????????????000??0??????000?????0???????????0000???0????????????0??????????????????????????????????????????????????????????????????????????????00????????002000000000000?0000?000?00?????????????????????????????????????00?????1????????000?000?01?010111?101??1?0020?01?10??0?0???????????????????????????????????????????????????????????000000010100010?00?????????????????11?110??1?????????0??1?????2?0?????????????????????????0??????????????????????????????1?01110???111111111021?0000????????????0?????0?0???00???????????0000000???00??0?????0???????????????????00?0??00000001???0???????01?????1??00000??1???????

Bistahieversor

?1102?????????10?2200002?01110000?001201000?21100000??????????00000000?20??2?000000101012??0????????????????????????????????????????????????????????????????????????????????????????????0??????????????2???????10??00??00?0??0???????????1200000011?00?00213?0?112??????0000000?????????10??0????00001???0?0????00???????????????????????????????????????00?????????????????????????????????????????????????????????????????????????????????????????????????11??????????0???????????????????0?1011001000?1010101?01011101010101002010001001???10000??00000020000211000010000??1111111?1?1001011001211111?01000?010011111??????11?111101?10111111100?11212101100112?10??2?00????111?????????????????0111?1???????????????????????????011?????01101111?120010000????????????1??1?0?0???????????????????????????0??0????????????????????????????????????????11001??????1??????????000?11???????

Albertosaurus_sacrophagus

?1102??000000?10022000022011100000001201000021100000?00010000000000000020??200000001010120????0?10?001?100?0????000??0????0???????000000010000???041001031200110000001012001000110??0?00?1000??00100000200000001000000000000?000?0?0010?01200000011000?0021310111210201100000000???00000100?00??0000010010000??0000000010000?0?0????????0000020000?00000?0001001?0?0010000?000000000000010??0?????000???00??00?0000000002000000000000000000?00??0100000??020100?000??0??001100?0110?000000101110110010000[01]1001[01]100101110102011100211[01]1[01]1101?001000000100010201002001100100011111011111111101011001111111000[01]000111[01][01]1111000010110111111110111111100111212111100?12?10?121011???????1?????????????????1111102011111?1021111110211101??1??1???111111111122010000000??00000101?011010?100010?000??00?000000100?000000??0?00??110??00000000??0000?0000000001011001111010111????0100000011???????

Gorgosaurus_libratus

?11020?00000011002200002201110000000120100002110000010001000000000000002001200000001010120?0000010000101000000?000000000000?00001000000001000000004100103120011000000101200100011010010001000?0001000002000000010000000000000000?0?00100012000000110001002131011121020110000000000000000100000??0000010?10000??0000000010000[01]0?0????????0000020000?00000?0001001?0?0010000?00000000000001000010??1000??0000000?0000000002000000000000000000000?0010000000020100?000100??00110000110?000000101110110010000[01]1001010010111010201120021101[02]1?010001000000100010201002001100100011111011111111101011001111111000100011111111??00110110111111110111111100111212111100112?10?121011???????1????1101????1?0?0111110201011111021111010211101?011?1?2?111111111?2201000000010000001011011010?100010?000010000000001000000000000?0000110?000000000??0000?000000000101100111101011110??0?0000001100?000?

Alioramus

?01020?00000011002200?0?20111000000012010000211000001000100000000000000200?20??000010101?000000010000111000000?00?00?0??000??????????????????????????????120011000[01]001012?010??????001000100??0?01000?02??000??10??000000?0?0000????010??11000000110001?0213101?12102????000?00000000000100000??00000100?0000??00000?00100?0?0??????????????????????????????????????????????????????????????????????????????00?0???000??2000000000000000000?0??0?1?0????0020110?00???0??0011000???????0????00111?2????001110111110?0111010100120121001021110101110001000010200001000000101101112011111111001011001111111?101110011011101000110110111101101110111100111212111110??10000???000001000011110?101221011111?????????????????1111010??????1?1111121??00?111?12?0100000??????0???0?1?1?010??000???????????0????01?0000?000000?0????????????0000??0??00???0?000?1??1101111?1?1??1011??0?000?11???????

Teratophoneus

?1?0???0???????0??2??????0111?00????1201???????0000???????????00??000??????????000010101??1????????????????????????????????????????????????????????????0????????????????????????????????0??????????????????????????0????0????????????????1?0000?01?????????3?????????0???000????????????10??0???00?001???0?????0???????????????????????????????????????????????1????????????????????????????????????????????????????????????????????????????????????????????100????????????????????????????????????????0????0?01??????????????2?021?0001??10??????????0???01?1??????0???01??1?1??21?111?100111101211?????????????????????????011?1?120?1????1??1100?11?????????11????????10???????????1?????????????????1?01???1????????????????????011?1?1???????????????000??????????????????0?0???????????????????????????0??0????????????????????????????????????????????1??????1??????????00??1????????

Daspletosaurus

?11020?000000?1002200002201010000000120100002110000010001000000000000002001200000001010120100000100001?100?0????000??0????0???????000000010000???141001031200110000001012001000110??0100?1000??001000002000000010000000000000000?0?0010?0020000001100010121310111210201100000000???00000100?00???000010010000??0000000010000?0?0????????0000020000?00000?0001001?0?0010000?000000000000010??0????1000??000??00?0000000002000000000000000000?00??01000000?020110?000??0??00110000110?000000101110120011020110021110211110102101201210101[12]111111111111101101020000211001011[01]11211212111111100?111012211111110111001101111[01]100111110111101111111111100111222111101112?10012101100011101111021111???????111111?211?111?112111??1??111?1??1????2?11????11?12???00000??11000????1??1?010?1000???000?1000?0??????0?000000000???0011000?0????????????????????????111011110??1??????????000?11???????

Tyrannosaurus_rex

?12020?01100011002200002201010000000120100002110000010001000000000000002001200000001010120100000100001010000001000000000000?0????0000000010000???1410010312001100000010120010001101001000100010001000002000000010000000000000000?0?00100002000000210001012131012121020110000000000000000100000??0000010010000??0000000010000[01]0?0????????0000020000?0000000001001?0?0010000?000000000000010000????1000??000??00?0000000002000000000000000000000?0010000000020100?000?00??00110000110?00000011111112001102101[01]0[13]01102111101021112010??10121011111011[01]1101111021010211001011111211[12]12111111100111121230111111111110110111111111111101102011111111111112112221211011121111121121111110111110211112111111111111021101111112111111021111111122112111111111112211000000010000001011011010?1000100000??0000000001100000000000100001100?10000000??000000000000001011101111010111101101000000110010000

Tarbosaurus_baatar

??2020?011000?10022000022010100000001201000021100000100010000000000000020012000000010101201000001000010100000?1000000000000???????000000010000?001410010312001100000010120010001101001000100010001000002000000010000000000000000?0?00100002000000210001012131012121020110000000000000000100000??0000010010000??0000000010000?0?0?????????????20000?00000?0001001?0?0010000?000000000000010000????1000??0000000?0000000002000000?00000000000000?0010000000020100?000?00??00110000??????00001111111200110210111301102111101021012010??10121111111011111011110210102110010111112112121111111000111212301111111111101101111111111111011020111111111111121122212?101112111112112111111?111?1021112??1??1?11111102110111?112111111021111111122112111111111112211000000010000001011011010?1000?0000001000000000110000000000010000110??10000000??0000000000000010111011110101111011??00000011????0??

;

ccode + 2 15.18 23.24 26 37.39 44 62 65 71 73 88 102 107 110 113 116 118 120 122 129 145.147 151 153 160 162 165 167.168 170 174 177.178 180 194 196 199 216 221 231 233.234 237 241 249 251 254.255 260.261 264 267 269 278 286 291 298 308 315 318 320 325 327 346 350 358 363 378 383.384 386 392 396 400 408 412 414 416 419 424.427 432.433 442 444.445 458 461 474 481 484 487 493 498 519 539 544 555.556 559 577.578 604 612 621 627 630.631 633.634 641 647 650 664 668.669 683 693 697 701 710.711 714 726.727 762 779 804.806 817 822 841.842;

proc/;

**4 Modified dataset of Lee et al. (2014) – Data matrix**

xread

1549 121

Herrerasaurus

10000000000110000000100010000011001000011000000000000000011001101100000020000000000101000000000101?0??0000000001000000001?0??00000???0?????0001010010000000001000?0100200010?0012010??0000000?00100001010010001100000000001000000000?10200000000??10??00121110000000?000000?0000001?000030000000?000000100100001000001000101101000100002111001????????[01]?0?02000100000??00000000000001?0000101010010000200100?00000000001?1?000101010?00000000101011020000000000010?000011000000?00000[01]1100000100000100001000010?0000100?0?00????????000000000?00100?00000000?0?101000000?00000100?100?0?0000000000?000000001?00000000?0000000?00010000000?000000???010?000?0000000?000000000??20?00000?00010????00???01?0?001000000000000001??000?0111??????00????0001?00?000?001110000010??01?0??0100100000000?00001???0?01?0001?000000?00000000?0?010?00?0?0001?010?000000000?1000??0000000??100?00000000?100000???00?0001000000000000?0???001000000???0?1?0000?001000?000000?0?000????????000000000000000000?00?000000000000000000?0???100?0?000?010?0?10000?00?000?0000??0?0?0?0?110???????0?0?0?00000101110???1000??00??0000???0?0?000????000?00??0100???1?0???????000000?1000????00?00???0??????0??00000??00??0?00??00?????0??????????????0???????0????0??0000000??0000000?001?0?000?0?1000000?000000??0?????????????0???000000?0000?00000000000?00?00?????0?????000000?00000?00??0????00000000011?0111100??00000000000????0000000?000??????00??0???000???0?000?00000??00?0???0000000?0?0??00??0000?00?00?000000010000?0?0??0??0???0?00?000?000?00?010000000000?00??000000?0000000000?01?00?0000?00??000???0?01?01?0???

Achillobator

1???????????????0???11?0?????010?000011?0??0????????????????????????????????????????????????????????????????????????????????????????????????????????????10???0000?0?0????????????????????????????0?0?????????0020?1100?20010???00121??020????10???11?????2[12]?100100110002001000??????????????????0??????????????000??1???0??????10???????????12????????????????0???4????00??0?10?0??1??10111000021100012010?210?12?1100011?21000010101?01001102200101110?0100?00?0?010221???0?????1?23??????0?0?0??0?1?1?11?00?0??????1?1????????????00?010??????1??1???0?????1?1???????0??1?0?0?01????0??1??1?0010?00???1?????0??20???????0001????0?1?????????00??2110?????0?0?????????????1??2?????????0?1?????0???0???1??01??0??0?00???0????????0??????0??????????0?????0???????1???????000???0????????11??????????00???????1??01????1????0?????0????0???????0???????????0????0?????00??????0???????0???0?????00??0?0?0??????????1?????????00?00??00??00?00??0?0???0???????0??0??0????????????????0???????0?????1???????????????????????????????????0?????????????????00?0?0???0????1??????????0????????????1????1??1?????????????????????????02?00??0?00100??????????10????1?0??????0???0?????00?001??00?0???0???????1??????0?0??1????????????0?????????????0????????????0??0?0?????0?00?????????????????????????????????????????????????0???????0??0???0????????????????????????????????????????????1???????????11??????0??????0?0??0?????0???00????????0????2??0????????????????0?10??0?????00???0?0100?0?1?1????2??00????0???????0?????????????0???????0????????????0?0?0???000000??0????????0???????0?????????????????

Afrovenator

10??????????1???????1100???10000?00100120?[12]0??????????01110201111101?00????????????0000010010???0?0??0001?0?0???????????????????????????????????????????0????0000???0????????????????????????????2???100012?11120?110???001000???111??????000??0001???0???????????????????????000?2??0003010?????????0110?011111000100000?0??0???1???1?[12]???001??????????????????0000?0???????0?02?001?0011101110?1?1002?00010[01]0110102?01??0??00100111000??10?110010021???0???10??????221?00011??1?0?[01]12?1??010000?0000??111001001??1?????11?????????00???000??0?00001??00????0?10?0?11?0??0??0?0?11?0????00???00?0?00???1?0???00?10?0???0?0??00??101??0????00?0????11????0?0?0??0???00??11?0??20????00?0?0?0??0?0??????????1?0????02?????1?????0??0???1?0??????1????0?????00??01???????1?0??0???2??1????1?????0?11???0????11?000?01????0?????1?0?????0??1??0???0???????1?0?0??1?0????00???????01?10???????1?????10????011??0??100??????????????????00???00??1000?0??00???1?????????0????????????????0??0?????00??????0????????????????011000?10??0??11????0???????0??????????0?000????1???????????????0???11??1????1?0???????101?1????????0??1??0??1???????????????????????0?0???0???1?0??0??????????????0?000??0?????1?0?0?0??1?0?0?00?0?0???????10?1??1?0?100?10000???10??0????0??????0??0?10???001??????0?0????0??????????????????1001??????1????0????????????????????????100??0????00???????????????10???????000100?????0??????0?0????????????00??????000????0?00?????????0?????????????????0?0????0??000?0??1????1??????1???????????0?00010??0?0??1?0??0??01?0???????0????0?0?00????0???1????00???????0?????0???????????

Allosaurus

100000010001110100001100110100001001001[12]10200010000010111111010001?11001200000000111010010001001010101001001000000011111200001011000020201010010100100001000000000000000100101110001001100001001121011011120100200110002001000100011010200000001001000000221100001000001001100000020000040100010001100110101111100011000010210?101?10102?22001??????013000000100001000001100001010001000111011102100012101010101001111012010000110101000012001[12]01100211000001101[01]0110221200011201100112210001000010000001[01]100100[12]011000001100???????00001000012001001000002001?10111110000000010111010000000110000?00000000100011100000000000000010100000000000000211100100100100020000001010?2000100010001000000000?01010010100000000000011??001001111000000001000001000?00100121111000110001001101001011100000010000000000?0000011100000010110000000001001000010012011000010100000?000100001011100011011[01]0110000000000000000000100000110000000000000??00?000000100000?0110000000100????????00000000010100011011000000000001000000000011000000000001110001000000000?101000000001000011000000??0000000010011001000010010000001000100001000000100000000?00000000000000000100000?1000001100001?0?000000?10?000100001000010000000000000000000100000001000001010[01]0000010010?1000000000000000000001000010?00?000000001?0100000000???00?00110010?00100000000?000?0?????0????0000?001000010000000000010000000111000?000?000101000000000000000?00000??0???0000000?00010000000000000000000100000000000000000??000000000010100011100000100000?00??0000011011010001?011100010000?01000000000000000000000??0000000?0000000010000010000010

Alvarezsaurus

?????????????????????????????????????????????????????????????????????????????????????????????????????????????????????????????????????????????????????????????????????????????????????????????????0?0???????????10000000[12]100??0????11??????????????????????2?100??????0?00????0?????????????????0???????????????????????????????????????????001????????302000??0[12]?1?????0001110??0?001??011100000010?00[01]01??1?1112?100????????????????????????[12]2001001???????0????????22??0???1[01]01?0[12]?1211?00?[12]0?010???0010?01000????100?01???????????1??????????????????????????0???????????0?0?11?0??????????00???0????1??????1??01????0?0??0???00?0????????10?0???0??????0?0???0????????????2?????????0?????0?0??????????0??0?0??????????????0???1???????????????00???????????????1??????0?????????????1??????????????????????????????????1??0?01?????0????????????????0?????????0??????????????????????????????0010???????????????????????????0?????????0?????????0????1??0?????00???????????????1???????0?1?????0?0?????????????1????????????0????1?????????1???????10???00???0????0??0??????0????????????1????????0?00??????????????????????0???0?0??010000???????????0???????????1??1???????????????0????????????????????1?0????????????????????????????????????????????????0??????0?0????????????????????????????????????????????????????????0??0???????????????0??????????????????????????0??????????????????0??????1????????0????????0???10?????????????1???????????????????000??????????0???000????????????????1???????????????0??????????00??????????????????????01?00????000???????????0???????0?0????0??????????

Alxasaurus

????????????????????????????????????????????????????????????????????????????????????????????????????????????????????????????????????????????????????0?0101000?010?110??011???????0???????????????0?0???????????1???00???1?????????[12]1?0?11000?0?1??10??0?01211?????????????1?0?00102?0010?01???0??????001??011010??0??1???????0?111?2?????2210?????????3?101??0?????1???01????0??0???????1???0???1?????????1????0?0??????????????????????1??????2?1???????10??0?00?0??????0??????????????????????0?0?0?0????????0?????????????????????0???0????????????????0???????????0????????????????????0???????????????1???0??0???0????0???0?2????????????0??????????0?????000???0?0??0??????01????002????????0???2?1??010????????????????0?????????????????????1??0??????????????????010????????0?????????????0????011???01???????0??????0??0???00????????1????????????1?00??????????????????????1????????????????????????????000?????????0????00?????????????0?0???0?000????????????????0?0???0?????????0?????????????????????????????????????????????00??????????0?0??????0???1?????????0??????0???????????????????????0??1??????????????0??????0??010?000?00???010???0????????????????????????????????????????????0?1???1?1?????????????????????????????????????????????10??????0????01?????????0????0??????????????11??????????????????????0???????????????????????????0??????????????0?????0??1?????????0??0?????0?????????0??0?????0???00???1??00????12??0?????0????????????0?????????????????????????????0??????0?????????????????????????????????????????????0??0???00???????0????0?????????????????????????????

Anchiornis

00101?0??0????2??????1????1?????0??1???0?11?????????????????1???1????????????????????????????????0???0??????????????????????????????????????????????0?00??001?2?2????????????????1?????????1?????0?????????????????????????????????????0?????0??0?????????20???????10??2???????11?0??0??1?????0000010012?111221???0101001212?0??12?1?????22?1???????01?????????????3???1?11??0????0??0?01010101110?0?020011?11?11?110?????????????012????1?????????1???????????????????????2???????????????011????1??01011???0??10?1?01?101?111111110??????11?????00????1?0????00?1????????????????1??0?????????????????1???????????0?2???0???00????1???1?000???0????????????????0????????????20?01????0?????????????????????0?0???[02]?????0???????????????????????????1??????????????????????????1??????0?????????????0???2?????1?1????????01??????0?0100??????????????????0??00??0??????????????????????????????????0????????10???????1?????????0?0?00???????0?0?????????0?00??????0???????????????0[12]?????????????0??????????????????????????????0????????????????????????00???????????????????0?0?????1??????1???????????0??????????????????????0?1111???01?0??0??01???0????0????????????0???????????1???1??0??????????????????????1???????????????????????????????0??????????0?0?????????0???????0?????????1???0??????????????????????????????0??????0???????????????0????????0??????????????0?????0????????????0??0?????????????1???1?0?0??0???00???00?11?????2??00??????????0????0???????????0?????10011??????0?0?2???11?????????????0????????????????????????????????0?00?01??00000000?0?1?0?0????0???0?????????????????

Apsaravis

1?????????????????????????????????????????????????????????????????????????????????????????????????1?????????????????????????????????????????????????0000??12??????????22???0?????0???????????????0?1??????????100???????????1?????1???????????0??????2????21111111?100121010201110?11010111111[01]0010?1012?111300???????1???1??????2???????22???101?????8??20011?41??30?????0?????0110?0?11110000?[01]?????[01]?????01011??0?0010?01021?2111000?1?010[12][12]001??112??2??0???????????????????1?????????12??01110??10011?0111?????1001001????????????11??1????0?0???????0???000??????????????????0??00??????11???1???0???1?????21?1???00?0???0????????0???100???????????????????????????????2??01?????????1???0??????????2?0000?020???????????????????0?????0?????0?00??????2??????????????????????????1???????????????1????????????0???0???2???10?????????????????????1???10?0???1?????????????????????????????????????????????????0???????010011????0??0???????????????0?1????000??????????????01?????????1?????????????????????1???????1?2??0?????????1111?0???????????211???????1?????11???1????0???????1????1?????????1???0???????????????0???0?0??1??1?1111?1???01???0??1??????1????????????????????????0?????0???1?????????1??????????????????????????????????????????0???????????0?????????????????1??????????????????????????????????????00?1?010?????0?100?0???????????????????????0??????????????????0?????????1???????????????111?00?????1?????????1??0???????????0????0???????????1??01110????????????0????1??????????????0????????????????0????????0???????????0??????0???0?1???1???????0??0??????1??????11??

Archaeopteryx

101000010000?0200?0011001111??10100000110110000101001101000001111100?1?00?00?0100101?0?0?????2001000?001?01[01]00011?00001111010?0010?01012?11?1?1010??0010010020212??00?00000???11?1001000?0001?00001??0??????0?010?1000?2??10001?0?11??0[01]??0001010????0?0012011?111?1000200101001101000001011000000010012?1112010?0010100121210??10?11101?22001??????013???00??0312230??10?10?0??0000201010101002000?012011121201111?00011?010100100[01]2[12]10110102[12]?010[01]110??10?0000???1???11012?0?11?023121101011000?1?110011?0110120111010011011101?1100??10?0?0?00?000?0?100?11?001100[02]01???00?0?0111??00100???0?1??0?0?01011?00?0201002?0?110000020?10101100000?002100?010?000?000??00?0?1000?20001?001000??0???000??01??000?000000200??00??000????1?????00?00?????101000?000?01????1???????[01]1?11?00?0??1110??0???00000002?0?10111??0?00??011000101?0110???0???01?002????000110000?0??10??00?20?1?01???0?000??1?00?11?0?001??000000?001????0?001000001??000000?01110?0?0?01000??0?000???????????00002????000000?000??000???0??????001?0????00?010000010?1?0?000010??0?1??000?00??0???110??0???01?000??010011??100111?0???00?010??0?00?0???0?????00?1?000000?11110??01??1??00?0??0?0??????0???100000???1??01??00?00?0????1?0????00???1?0?????????0???????1?????????0?0???????0??0?0????0?00?0?01????0??0?000??1110112111??1??????????????????0?00000000?00?00?000??00?00000000?0?0?0?00????0???00?00000?11??????01?0??0???0001?????11?0?1?001??1???0010?10?000?1001?000?0??0??0??0????001?0000?0??00????10011??11011?01210?11?1?0??0??0?0000?????????00???00??0?????0?0?0??00000010000000000?11100?0?????000??0????0???????0??

Archaeorhynchus

111??2010110?????????1???211??101?011?????[12]000??10?1?????0?????????????0??00?000110?????????????0?0???0??0?[01]??????????????0???????????????????11????0?00??12??????????0????????????0?1?????????????????????????????????????010??????????????????1??????0??201??111?1??02??1?1011101??0101011??0001011012?111301???010110101101??11?12????22???101210115????01??4??????????????????1??????????????????????????2?????????10?1???0?2???200???????????????????????0????????2?111????1??[12]3???1?12?00???0??0?011?0100???????0?011?11111?0????????1???00?0???????0???0?201??11???????0????0??10?0????????1??0?????2???????10?2???????10?0?????0???2?00?0????????????????0???0??????0021?01????0?2???????????????????0?0???2????0???010????1????????00???????1?0????0???????????????01?0[12]????0??????????????0?????????001??0??0????0???0?????11????????0????2????0?0100?00??????????????1??1??????1???????????11?0?????0???????????????10?110000???????????0?????0100??????00???????????0??01?0????????????0?0?1????????????1???????0????0??????1????00???????????0?????????????????11???1?0??01???????????1??????0??1??????????????????0??1????????????1????????????0???????????????????????????0??????????????????????????????????????????????1???????????0???????0??0????????1?????????????0?0????1??????????????????????????????????11???0?1??10??0?10???0?0????????0?????????????00?1???0????????????0????????????????????1?01?11?1???????10?1?????????02???????????????0??0????????1?1???10?1???????0??02???1???????0??????0????????????????0???????????????1????????11?0?0????????????????????????????????????

Aurornis

1010?0010??0??2?0?00110?1110??1???0000?000?000?110?0?101?0?02???1??0???0??01?0???0??????000?2????0??????00100???????????????????????????????????????0010??00?02?2??0??00????0???01????????0?0???????????????????????????????10????????????????????????????201??11??????????????0??0?????2???????0100?012???120????11010?1?02?0??11?10????220?????????12??2?0???21223???1??0??0??00001??010101??001???020?0??01?11?????011???????1000?0??110?0????1?????????????????????????2???????????????010????1?????11???0???011?00?001??1??????00?????11??00?000???1????0?0??11???1??????0???????0??0??????????????10?0?0??????0????????00??????????????00?0?2?0????????????0???1???0?????0?01??0???????????0???????????01???02????1?????0??????????????????????1???????????????????????0?0?????0???????????????0????????0??1????0????1??????1??10??????????????????????0???0??????????????1??0??????????1?0???1???0?????????????1?????????????0????????0?0?1?????????000?????0??????????????????0?????10???0?????????????????????????0??????????????????????????????00??????????1??????????0????????????1????1??????0??0????????0?????????0?????1?????????????????0?????????0???????0???0???????2??0???0??????????1???????????????????????????????????????????0??????????0??????????????????????0?0????1??????????????????????????????????????0???????????????????????0????????0????????00?0?0?0????????????????????????????11??????0????????????0??0????????????0???????????????????0???????????????1?????????121??1???????0??????????????????????????????????????????00??????00000??001????????????0?0???????????????

Austroraptor

20??????????????????1?0???????11110000?10?10?????????1012?1010?????0???12?01????01?????0100?0???????????????????????????????????????????????????????0?10010002202??10?[01]000???????10??????????????0?0?????????1010110???2?0???0?01?11??????????0?????????1?????????????????????000?1??00?301???0??01?????0??1?000???10???1????????????1??????12??????????????????????????????????????????????????????????????????????????????????????????????????????1??????????????????????[01]???????2??????00?1??111???1?1?????????1?0101?1?0?????????0?1????0???????0?10??0??????????00???0?10??0??0?????00????????0????????????????0?2????????00???????1????10??????????????0???0??0????????????0??01???????0??0?00??2????0?0?0???0?????0?????????????????????????????0??00???0????????????0????????0?01?????0?????????0?00?00???????00?????01?????011????0???0?????????????????0????11??????????0???????????0?0???????0???????????20???????0?????????????????????0?????0?0???????0???????????????0?????????????????0?????0???0???????00?1???????????1???????0?????????0????????0????????????11?????????????????????????00?0????0??0???010??????0?10?0?????????0????12?1??00?0??10????1?00????0100?????????00??0?????????00??????????0?????????????????1???????????0???????0????0???????????00???????0????????????????????0????????????????????????0??????????????????????????????????????????0???0????1???????????10?????0???????0?0???0????????00????????????????0?????0?????????????0?0????????????10?01?????????12??0?0???????????0????????????0?????0???????????????0?????0?000???0?0????0??000??????0?????0???????????

Avimimus

0?1?????[12]01??0???1?0????????10??????????????000????0???????????????00??0[01]?0?11111101??00??0?21??0???0?00?01010?01??0011?210??10?1???02?2????????????????1?12????????????1??00010?0??2????11?010??0?1?01?101?000101100??20???1?1?11[12]1?0?00??00001??1??10?0021?0010??1???1??1????0??1?000130?100[01]??10???1?????1?????????1??????0??????????????????????????100??1??0???????????????00?0???1111?0001010?0000100100?1011100011111010010102000100012201101210??10???0?1?01?221101200??10?231??1111110001201100111011002???01001110??????????111??1??200?????0?000??0?00?????0???0?0?0????0?0??1?????000??0??0??0?1??00020??0??00?00??0??0?0??00???001?0????0?????00????00???0???????21?0??????0??0???10???????0?10??10???????0???????0????1????0?0???????????0?1??0???????1????010??????0???????1?1????????????2???0?????00?????0???0110??0110??????????0?20??10??????0?0???????????0??1?????1???0?????????????1??0?100?????0???1??00?????0???00?00???0??0???????????????00?????????0?????2?????????01??????0??????????????????????????0??0???1??0????1????0???????00??????1?????????????1??????????1????1?????01??????????????????1???0??????????????????????1000?0??0??????1????????????????????0?0??0??????11?000010?1?????1??????????????????????0?00???????????0???0??????0?0???????0?001000??1??????????????????????????????0???1???00??0??????????????0????????????0????????100??000???1?0???0??????11?00??1??00???00?1?011?????????????????00002??????0?????????????????????????0?0??10????0?0?1?1???????1???????????0?0??????????00?1??????????????0????????00???????????????????0??????0??????0??????????

Balaur

???????????????????????????????????????????????????????????????????????????????????????????????????????????????????????????????????????????????????????????????????????????????????????????????????????????????????????????????1??111001???0?10???0?????12201100?1?100?2?010????11100000?0??000?0001??22???11010?00101101??211???2?1???????01[12]0?1?????5??200???????????0?1???0?????0??????100?????????20?1????0???110?21??0??20?2??0?2001??1???????????????????0?1?????2?0????????????????1100000000000011?1?001200100112?1??????????0????????????0???????0?????0?10?0????????02???1???0???????????1???????1????????0???0?101?00????????1???0?0????????????????200?????0??????2?00??0??0?0??1???0?00????1????0001?02????????????????????00???????????1001???????????1???????0??????????????????????0???0??????00??????01???11?10?????1?1????????????????000?10??0?????????????????????????????????1???????????????????????1???1?00??00???1???0?????????????0??????100?????????01???00?????????????????0?????????????1?0???????0??0??1?????1?0?0??00?0?????0???0??????1???0??11???0????0??????????????????????10?????????????????00???0?1?00???0001?101101????01?????????????????????????????????0?????0???00????????1??????????????????????????????002?????????0???????0???0?01????0?????????1?????111??????????????????????????????1??0?????????1?0???0????????0?????????????????0???????????????0??1???????????????0??????0?????00?????????????2??0????????????1??00?1??0??????1?????10???????????????0????????????????0?????????????1?00??0?????0??????0???????????0???0?1???0??00???0?11?????11??????????

Baryonyx

10010001001?020001001000??211201?111000?1?1020?1??1000011?1102?????????020???0????2????0????????00001?0??????0?00?0011?11????11010???????1?????????10000100000000??100?000???????000???1????1??1?2?0?10011200102001000110[01]1000?101110000000100010010??00031110000?00000?00000?00002?000030110010002????????????1??0????????????1?????0?0?[01]?002?????0013??????0??000??1?0?1000000??00???01110111??101002?0[01]?100011?10??010200?000[01]0101000?11001101100[12]1[012]?100??10[01]?001022110001???11??1??1????????????????????????????????????????????00?000??????0???1?10??0?00?1?0???100??0?01???0??0?00000?11?0?1?0?1?0?0020?001?00?020???0???0010???????00??????01?100??0000?0???0001???10??????0111101??00?0???????2?1?01???0??0?1?0?00?10?0???110?1??0??[01]0??000????00?110011??1???0111100110210001101?100000??00??0?0????1001?00???0?00?0?0??0???00????21??01???2??0??00???0001??1?0?001??010?11???0??1??1???0010?01010??0000???00?11??1100000??0??000?01??0010000?????10001??00??????????00?010?000?0??0????0?100?????0??0000??1?0??0?0001??????1??000?0000?101010?0?0??0?00????110?00?10?0?0????0?1011???????1110?00?????111???0????0??10?0??1???0????????0???????1??01?????0??1?0?0???010?0????1??1???0???0????1??1??0??1?0100?0?0?0?????0??0?011001?1100100?010?00??00?0?0???????00?0??000001?0?00?0???????????????0???0????1?001???????00000??0??0??????0???????????10???0?00?????????0???000??10????0??0?0101100000????0?0???0?0??0?????000??010??000100?000??000??0??00???0?0000???0?00????????00?0??0??000210??0??0???????????110???11110001?01100?????0???10?0??0???000000????????0??0000??00?0???????10????????

Beipiaosaurus

????????????????????????????????????????????????????????????????????????????????????????????????????????????????????????????????????????????????????0?0101000?0???1?0??0?1?????????????????????????????????????????00??2?00????????1???1????0??1??????????2?10?????0?0?20?1?000010????102??10?0000?1?001?101101???001100020210??11?211?2?22?02??????01?????????20?????????0??00001??00?01[12]10000010000011100?11?010100????????1??????????0????1?0?10?21????0??1?0?1?1???1?011???????2????1?00100?0?0?????????01??1??10?????10?1?0???000???0????????0???????????0??????2?????0????0??????????0???0???0??0?0?????00?2000?0???00?00??2??0??????00?0????01????0??????0??????0?1????????1?1???00??????00????2??????0????????????????0????1??????????????0?01??????????????1??????????????????????????????????0??1???00??????00??01???0??0????1???0???1????????????10??????????????????????????????????????0??????????????0???????????000?000??0???00?0????00?????0000?????????????????0??0????????0?????????????????????????????????????????0???1???????????????0??0???0????1??????????0????0???????1??????????????10?????????????????0???????????????0??????0?????0????0????????0?1????????0?0?0?0???????????1?0???110????????????????0??????1???????????0???????0??0?0??????0?????1????0??0?0????0??0?0????????111???????????????????0????00??0?????????????????????0??????????????0?????0??1?????????0??0?????00??????????????0??????00???0??00????????00????0?????0???????????????????????0??????0??0??0????????????????????????????????????????????????????0?0????0???10?0???1??????0?????????????????????????

Berberosaurus

?????????????????????????????????????????????????????????????????????????????????????????????????????????????????????????????????????????????????????????????????????????????????????????????????0?0?????????00?0?1000?0?????0?1??2?0??????????????????????????????????????????????????????????????????10??????1?????00????????????????????????????????1000?????????????????????????????????????????????????????????????????????????????????010101?02????0???1?1?0?112212?0?1?????0???????????????????????????????????????????????????????????????????????????????????????000???0????????????????0?0?????????????00??????????????????????????????????????0?1??????????0???????????????????11????????1??????0??????????????????????????????????????????????????????101??0?1????????????1??????1?????????0??????????????????0????????0??????????????????????????????1???????????0??????????????????????0?????????????????????????????????????01???????????????????????????????????????????????????????????????????1???????????????????????????????????????????00?????????????0??????????????????????????????????1????????????????????????????????????????????????????????????0????????????????????????????????????????????????????????????????????????????????????????????0????0?????????????????????????????????????????????????1????????????????????????????????????????????????????????1?0????????????0?????????????????????????????????????????????????????????????????????????????????????????0???????????????????????????????????????1??????111??????????????0?????????????????????????0?????0????00?????

Bonapartenykus

?????????????????????????????????????????????????????????????????????????????????????????????????????????????????????????????????????????????????????????????????????????????????????????????????0?????????????10?100002?1?????0??1??0??0001?1?????1??1?0????0010010???00?110???????????????????????????????????????????????????????????????????0???????2????????????0?010??1??????0?0?????????????????????0???????0??011?0??2??10???????????????????????1?????????????11??????????????????????????????????????????????????0??????????????????????????????????????????????????0??????????????????1????????????????0???????????????0????????????0?0???????????0????????0????????????0???????0?0???????????????????????????????????????????0???????????????????0??????????0?1???????????10?????????????????1????????????????????????????????????????????????????????0????????????1?????????????0????00??????????????????????1??????0????????????????????????????0?0?????????121????????????????????????????????????0??1????????????????????????????????????????????????????????????0????????????????????????????????????????1??00??????0?0?0?????????????????????????????1???????????????????????????????????????????0????????????0??????????????????????????????0??????????0?0????00????????????????????????????????????00???????????????????????????????????????????????????????????????1????????????00?????????????????????????????????????????0????????????????????111?????????0???????????????????????????????????????????????????????????????????????????????????????????????????????????????????????????

Buitreraptor

20??0???0????????????10?????101?1[12]??0??200?000010?00????????????1??0?1?120???0?1010??0???00?120?100??????0??0???????????????????????????????????????0?10100022202??????000???????10????1?????????0???1????1??1?1011000??????1???1?11???0???001????1??0??01201111101100?2?11?1??01?0??0?02?11?00??11??012????[23]000???1010?1?02????10??[01]??1?2200[01]??????01??1?0?????1232???10????1??0?001?10??101?02?1????????1?1??12?110?21??????00100121001001102001011??0??0??0???????????0?[12]???????2??????101?000?1?111011?0??01???1???1????????????0001???01??0??00??00??10?1?12????1?1?01??000??????0??0??1??????0?0???????0?0?[12]00??1????0?1?0???????0??????0?1?210???????0??1???00??0??????20??1?10???0??10??0?????2????0?0?01?0??0???0???????????????????????????1?0????????????1???????0????????0???????????????00?02???100?11????0??011?00????1111???0???01??????????0?000???????1??????????????????????1???1?????00???10??????0?????0?0?10??0????00?0?0?0?????0?????0?0????00???????????1????2?????????????0??00???????????????????0?0????0??????0????1??1???0?????00??????????1???????01?0?0????1?????1?????????????????????????0???????00?11??1??01001?0001??1?10????0????????1??0??1??10????????11?0??10??????1?0??????0??1???????????????????1???????????0???????0????0???????0?0??????????0?0????????????1?????0????????????????????????????????????????????????????0?????????????00?????0????????????0??000??00???????0?0???00???0????????00?00?????1?000????0????????????1???0???????????10001????0?0??12????0?????????????0????????????????1??????????0????100000??100??000??1?0???0?0??0??00?????????????????

Byronosaurus

?0011001000110200120010?11011011120000?201000001?000110100001?1????0???????1?????????????????????????????????0?11?0010?1??11?0??1?111012??????1?????0010?00010212??000000000?????11????????1?????0????????1????1???00??[12]1??0????1?10?0???????0????11??????????????????????????????????????????????????????????????????????????????????????????????????????0?????????????1???????0????????????????????????????????????????????????????????????????10??1???10??0?????????1????????????????????????0????????????????????101?????????????????0?????0???001000?????????????0?1???????????????10???????????0???0??0????21??00?????????0?0??0??????????????????????????????0??????0????0????0????????????????2???1???????????0?02????0?????????????00????????????00??????????????1??1?00????0???????????????1???????????0??0????????0?????????????0???01?0???1????0??????????00????????1??0??????0???????????0?001?????0???000??????0??????????????????0??0?????????????????21?20011????0????????????????1????0???0???0???????????????????????????????????????????0?????????????????????????????0?????????????????????????????????????????1??0?????????????????100???????0???????????110?00?????01???0?????????????????????????????????????????1???????????????????0????0??????????????????????0???????????????????????????????????????0?????????????????????????????0??????0?????????0?????0????????0?1????0????00?????1?0?????000????????1??????????????0???0??0????????????????0??????????????00????????002???????????????????????????????????????????????????????????000???0????010??0??????????????????????????

Caenagnathus_collinsi

???????????????????????????????????????????????????????????????????????????????????????????????????????????????????????????????????????????????????00000??120????1???01210101110200?1[01]0?011?11101????????????????????????????????????????????????????????????????????????????????????????????????????????????????????????????????????????????????????????????????????????????????????????????????????????????????????????????????????????????????????????????????????????????????????????????????????????????????????????????????????????????0????????????????????????1?????????????????????????????????????????0????????0????????????????????????????????????????0???????1???????????????????????????0????????????????0??1???0????????1??????0???1??????????????????????????????????0??????????????????????????????0????????????????????????????????1????????????????????????????????????????????????????????????????01???????????????????????????0????????????????????????????????????????????????????????????????????????????0?????????????????????????????????????????????????????????????????????????????????????????????????????????????????????????0???????0???????????????????????????????????????????????????????????????????????????????????????????????????????????????????00011????????????????????????????????????????????????????????????????????????????????????2?????0??????????????????1120?1111???0??????1???????????????????0?????????????????????????????????????????????????????0?????????????????????????????????????????????????????????????????????????1?????????????????????????????

Caudipteryx

0112?1111001?0200??11?1???11??1?1?00001001?0001111?0110?100?0?0?10001??0?????1110?0?000?000?12?0??0??10010010?????????????????????????????????1????100011?12?????0???11?1010???0200???0?1??0??10?0?010????1???????1?????0??0????0?[01]1???[01]0??0?0?1111??2?001211?0111?1?????01?0??0??1?????3????000000?0012?10?101?0?010100110210??11?1[01]?0??220010??000013?1?0?0??30121???00100000?00100?00111000001100002011??11?11??100011?2101001011200?1001?2?0110?1????10??000??????2??01[12]?0??1?02312?100011000?1?110011?011002?111000011011101?0100?01??010??0?0?????1?0?00?10?11?001??????0??110??0??00????0???0???010????0??2000?????00?000110?0??????2000?0?210????0?00????0??00???110??20?01?00?000??????00?????????0?00???01?0??00????0????1????????000???0?01?0?10?0??1????1???????01?01??0?0???11???0?????0?00?2?1??00?1??000????110001?0?01100?????????????????00100000??????????????1??1???0?000??1?0???????001????0??????01?????0?1??00000000?0?0?0???0?0???01000?????00???????????00?01?????0?00???????00????0???????00?0???00?0?100?0010?1?0?????????0?????00?0???0????1???0?100??0?0???100????10???1?0???00??10?????0?0?0???????0??0??100001000?0???0??01000?0??0?0???????00?10???0???1??????0???1??????1?0???????????0?????????00??????????????????0??????????02???????01?0??1????0??0?0????0??001?????????????????????????01?010??0??00??????0???????0???00?0?000?00????????00???000?11?????????0??0??[01]0[01]10000??0100?110000?0???00???00?00?????2?000??????????0????0?0???0?????0????01000???1??1??001?0?11?1?0??0??0???0???????????????????????0?????0??0?00?00??11000000?0??????????0???0??????0??????????

Ceratosaurus

1000000100021001022011000001000000010011101010110000100110110101010100002000000000110000000010000?0100?010000100000001?12?0?010000?0010???0????00??10000100001000?0000000001??0100100??100000?0110110001102000120?1100110010001110211101001001000010??0?031110000000000000000000002?0000401000[01]00020???1???01011?????00?0?0??0010011????0[01]2???????????[45]1?10100000000001001001000[01]?00110111111020?1?010210??00?01201010011?1010?00010?00101??01010110210000000111?00112[12]12000?11001000121??00?000000000??10001??????????????00???????0?0000?00010?00?1000001?00?1010?0?00?0000?00011?0???0000?100?0?100101001?00100000010000??1?00?00?10???0???0????01??0000100?????000001?01??200?00?00?1?10????0???1?1000?100?0??0200000010??010001110000??000110??00000?000011211?10011100?0?1110100111111110001000??00?00?0??0?1000????0?010000?000??10?000101?102??0?0?000?0??01??001000??01110000001000??0000?00000010?1?1100?000001??0100000?000??10?01?00001010?0?000000100000????????100010?0110?0???00000?010000000?010000001111100000?00???1110000000000???10?001?00??00001110?001???000010?0100110?1?01010??10?????1001100?0100011100010100?0000?0???0???????100?01?1?0010?00?001??000????????00000??00????10??000????1?0?00?000?????0?00?0?0001?0010001??11000??0000?00??0?0000000?00000000?00?????????????????0???01000100000?01001000000?00?00?????0????00????0000?00?00?0??1??000000000??1000??11?001101?00001????0000000?000??????????0?????0???00?00?00000??00?00001000?00?0?0??00??00?0?00?000010001110?0001?1?00??0???0100000110000?00000000101100?00???00000?000000000?????0??0?0??0???0?000?0???000????0

Citipati

0102011121120???11??1110121011111?000010010020201000110110022111000000000000110101011000000?01011000011010010000100000112100?110?1?010?21110101001101?01??12?????0???121101001102000100011201010?0?1?0??1????1110?100??10?10110?1??1?1000??00???111112?0012110011?110002001?0?001010?00020110???0000?012??11101???010100020210??10?10001?2210101100??14?1?1????201200??10?0??000010100001110000211010020101?0111011100111?2101001011100010000220?????????????????????????012??????????????10100?001???0011?010002011000?111??????????00010?110?00?00010?000?000000110101?0????01?010?00?00???0??0?10?1??10?100??011?00??0200??00????1??011?0000?0?2000?01???10???000?0??1?1???20?01?00?000??????0??????0???0?0?0??00?000001?000????11??1???1111???0?01???10?0??1????1???????10?01?0000?0?11???00?10?0000?000?1001?000000???1?0100???0000?????0??000?20???0?0100000??????????????1?001??0??0???1?0?????0?011?0000101???01???0?0??00?0000?00?????00?000001???0?0??0??0?10111110?0?0??01?????????0?0000?001??00???????????11?000??110100?0?0???????1????10????0?0????????1?????1?0?1?000?0?00??????111??????00???0?????0?0000??????0??0??110?0100000??????0??0???????0????1??0?110?000??????0??000001???????0????????????0??????????????????????????11?0?????????0??????0??0?????0??????011000??1?????????????????0????????????????1000???????????????????0??111?0?000?0111??1??001???000?????0??000?0?111?0101?10000011??0?0000?00??00???01000???????00?000????00????????????00??????????1?00?????0?0??01????0??????0??0???0???????????????0????????????00??0????0???11???100?01?1?1?????0???01??????00????????

Coelurus

??????????????????????????????????????????????????????????????????????????????????????????????????????????????????????????????????????????????????????100??0?????????????????????1???????????????0?0?????????01100000002001??0?00111100000?00?01??1???0?0021????????0?????????00001?00003011??0?0011?0?2???????0?????10??202????????0?????????????????????????0?0???0?000100000?0??0???0????????????0????????????????1213111000110??????????01[12]10101210?000?11?1?0?1?22?10?1011?1?0121?????0?2????0??????10?1??0???????????????????????01???????0?????????0???????1???????0?00?10?????00????1??011?00??0???1??0??10???0????????0??0????????????0????00?????0?0?0???????????????????0????0?11??????0????????0???0?0?1???????????????1?????0????????0????00????0????111???1110?????????0101?1??0?????0???0?0???0????????????000?00?0?0?11?0????????????????????01?00???0????????01?1?????0?1?0??????000????????????????????????00????0?????0?00????????0????10?0??0??0?????????000???0?????0????0????????????????000????????????0????????????0???000??????0????0???????1?0??0????0??????0?????0??????1???????????0?0????????0?????0???0??0?0????????????001????0?????????0???0??????????????0?0???0???????????001???1?????????????0???????????????1?1???????00??????????00?00??01???????0??????00?1??1??00000?????????????????0???????0??????????????????????????????????????????0????????1?????????0??0?????00???????????????????????????????????0???0???????????????????0????????????????????0?0?????0???????1??????????????????????0?????0?0????????????????0????????00???????????????????000???????????????

Compsognathus

1010?002?0?0?02??100?10010110?111001?0100??000?0??0??10100011?11???0????0??0??11??0????0000?0?0???????0??0?00??0???0??????0??10?????????????????????0010000000[12]0??000000000??????000???1?0001????0?0?0??1??????10?00?0??0?10001???10???00??10?0?01???0?0002010?000?00??10?1?0?00??2??0??3?????200011???1??012100??110?0?010210??11?12?0??22001??????01?????????[01]0111?0?01?00?00?0?00??0?11???????1????2????????11???0?213?[01]???0?1011100000100?????????????????0???????2??0011?????012?????0011000?0???0011?011??201110000110??0???0?01??????1???0??00?01??0?10?10?11??0?????0?00?1?0??0??0??????0????0?0??????0??1??110????0??00010?????0?0[34]000?0?210????????01??0??01???1?0??20?01?20??0???????00????1??0?[01]??0???01?0???0????0??????????0???0????1?01?0????01?1????1??????0?0?01?01?0???11???1?????000?0?00?100?1??00000??00000001?1000???0???0??0??????000?0?000????00????????1?00???0??0???1?00?00?01??0????0???0001????00?00?0?000???????010?1?0?0???0?000??0??0????????????00?01??0??00??????0??00????0???????0??0???00?0?000?0111?0???????0???0?????00?0???0???110??0?0????0?0??010011??1????1?0???00??10?00??0?0?0?0?????0??1???0?00000000???0??01?00?0????0????0?010??????0???1??00??00000??00??0?0?0?????????0??0??????0???????10??????????0???????0??0?0???0?000?0?0???0?0??0?00??00001?0?????000?????????????????010010??0??00?0????????????0????0?0?000?00?1??????10???000?11????????00??00??0?00????000?0??0000??0???00????0?000???0??00????10??0??0????00????00?0??00??0?00000??1101??00210?00?1?0?00??0???0?00?0????0000??00?00???0?00??0??00000000000000000?00?00?00???0???0?????????????????

Concornis

???????????????????????????????????????????????????????????????????????????????????????????????????????????????????????????????????????????????????????????????????????????????????????????????????????????????????????????????????????00??00?1?0????????0201?1????10012?110210110111010101111000101??12????3?????2?011[02]100001??1[01]?0?????220?111121111??????1????????????????????????????????????????????????????????????????20??01?1[12]??1?010??????1??0???????0????1???1???2????1?????????11110???0??2?111?11?1??101?01??11????????????1??????????????????0??0??0?12???????????????1??????????????11???0???1??0????0????0??0??20????????0??3100????????????0?????????????1??112???1?0??0?0??1???00??????????2??00?021???????00????????????1??????????110????????????????????0???????????1?1????????1?????2????00??????0????0???0?????11??????????????????????1??00????????????????????????????????????????????????????????????010??0?000????01?????????????00?????000???????????0??01?????????????????0?????????????1????????????0?1??????11??1?????0?????0??1???001?11?????11???1????11???????????1?????00??10?????????????????00?????0????????1???????0??0?0??????????????11??????????????????????????????????????1???????????????????????????????0??????????0???????????0?????????????????1??????????????????????????????0??0????1??11?10?0?111?1???0????????0?????????????????????????????????0?????????1????????0??????10??1000???11100????????01???????????????0???00??????1020?010???????????????0?1????????????0??????????????????0????????0???????????01?????????0?1????????????????????????????????

Confuciusornis

101002110110????01??0110111110111?1100100120000?11?1110?00022011110001?00?00?1???1010000000002000?1?0?1110????0?1?????????????????????????????1?????00001?12?????????1110010?1?1300??00?10010??0?0?0?0????????0???10????1????0??0?11?1?10??001011101?0?00120110111110002??1?1001102000101011?10001010012?1112010?0210110121101??11?10000?22001111000015??2?0??0412?30?????0??0??0110???1111?0?0?000??120??1?12011?0?0?010?01020?2010221?10010[12]??01011????11???00?1?1?????111?0??1?0?????10111[01]011?00??1111?0110021011010011011101?11?00?1??01??00?000?0?0?0??01?0?10011???????0???11??0010????00??11?0?010?1?00?021000??00?00001????0??0???0000?0?2??????0?00???000????0?1?01?20011?0?10?0??1???000????????0?0010?020???0???000?????????000?000??????1000?0?0?21????1?????1010?12?0??0??111???00???10????201??001???0000???11010?????11?????????0?0?2????110111?00??0???????????1??1???0??11??1?0?????0100101??0??????0??????00?010000000?00011???10?????0?000??0?000??????????10??01?????00??0?0????000????????????1?0???000?2100000?0?1?01000?10??0?????00111??000?10?????11?1?1?1??0100????1????1?????00??10??0????0?0?0?????02?1?0100?11110011?011?10100?0??1?0???????0??1??????????00???0???0????0?1?0?????0???????????????0???????????????????0??????????02????0??00?0?01????0??0?0????1110112111??1??????????????????0100110010000?0??000?00??0?0????0?0?00??00?1??????00???000??1?????0???0??????0011?????0??0?110100?01??00???110001????1?000??????????0????0?1?000?????10101010?1???1???0??02?0?1111????0??0?0?0????????????????0??????0?0???0??0??0??1??11?0?000?1???0??0???00??0??????0??????00??

Cryolophosaurus

1??????????????????????1????????????00????200001???010010111121?0101101020?1?00??01101?000101000010?01?0[01]00001100??0000?2000??0000??11???????????????0?????????????????0???0?100?????00??00010?1?0?0?????????0?[12]0?0??0????100???1010?0?00???01?0??10?????[01]????????????????????0?00????00?0?00?0???????????????????????????????????????????????????????????0?0?0??????????0??0?000??0?0?0??1?1?10??????0???0???010?1?0001???0??????10?01?01200100011?2?00000?00???????2?120???11?0?011??11???????????????????????????????????????????????0??001?0?00?1?1??0???0?1?1??1?????0?00??101?????000?11?0???00??0???0?0?0000??????0????????0????0???????????0???11?110???????00??0??????0???0?0??0?1?0???????0??????1?????00????1??1????????1??????????0????????0????2?01????1?0111????????01????????0?0000000?0??0?0???????000???00?010??0?00??00??0?1??11?1?????????????????????1?0??0?????0????0?0??0?????????0000?0010?????0??????0????????????10??00????0?01??0????????????????????0??????????????0?????10?0???0????0?????????1?????1?????1?0???????00???????????0??????0??????????????0??0???????????????10????????0???0?0000??????????????0?????????????????0???????????????00????????????????00???0????????????????????????????????????????????????????????????0??????1????????????0????????????????????????????0??????????????????????????????????????????????0??0????????????1????0??????????0??1???000?0??1????00????0?000????????????????????0??0??000?????0?0???????????0???????????????????01?????1???0?0??????????????0?10???1????0??00?00????0?0????0?????????????0?????????????0???00??????01????0???

Deinonychus

100000010001?0?001??11001000??101000001?0010001?0?10110?00001????1[01]0????2?0??0?????10?10100?1???????????10020??00???01?11????1?1????1?02?11?1?101???0000100000100?000??000???111?011?001?001?0???0100111112011120011000210101010?111010200000101??11??1012211001001100010010000010100000201100000011?01211011010000111000[12]1210?111?11111?22112????????4???????0212420001011002??0000201011101000?10001202112100121110??11?11010??0112000101102220101111?0?000000?0?10221101100111102212110[01]010001100111011000001101101012010????????00?0100010201101??000?0?10?02??1?0010?1000020110??001000?10010?000001001?00002000000000010000200100?1000000?10211??000?000?2001??0?0?101??20?01000?0001000010000?01010?010000?02?00?00?1??001?01??1000?1000???0?01000?0000?1??1?1?00?10010?00????0?0111??0???0?000?0020??011001???00??011?00?000?11100?00??01?002??10010110??0?0?000?????2011100???00?00?11??000000?0000?????0?100??1??0?00100?000??0??000101110?0?0?010000?0?100?????110?1100000????00???0?101??01????01??000??1?0000000?0?000?011?0?1000001000010?0?00000??00??110??0????0?010100100110?1????100100000010001????0???00010002?1?0002?0100??000100211000001??00????0?0?0??00000?0010?00000?00?????001?0000?000??1?0?????????0???????00?????01??00????00000?010??1??00000001?0000??0?00???1110?1211111110??????????001???01?000000??0??00???????????0???00?0?0?0?00?10?????00??0??0?110??????0?0??1???0?00????0?2?0???000??0???00??????000??102?00????00?????0?00?00100000?00?00??0000100?011?10??111000001?0??1????0?00?????????00?0?00?00??10001?????0?000010100001?00?1??00?0000??010000???00??????00??

Dilong

1001000100?2?0020?2011011100??10100100120100102000000001111011111?1010102??0001?000102?00000120?0?1?1?00010?01??????01??100??20?10?????2????????10??0000000000000?0?0000000???210101?1?10?0?1??1?1?0????1???00?10?000??222??00??0111?0?0??00???10???????0[01]10000000?0000???1?000?0?2??0?03?10???0???????1????10?0??0?01001?0210?112?201?2?22001??????????????????0?2????0??0???0?0?0????011?????201[12]?????????10?11?11??013?1???0??011100?0010022??1????0??0???1?????????????1?1?????????????0?1000100000011001?0?1????0?0??1??1??????001?10?00?200?0001000???00?10?0?1?00??000?10??10??0??000?1??0????0??11???00??100001?00???00?0?000?0?0?000?0???2?1???1?0?00????0?00??0??0??20?01?[01]0?00??1????0?????1???01??????00????00????00????1?1????0000????0?1?0??001??1??1?1?????1001?01?0100??111???10????10??0000?000?0??00?00??101?00?0?01?????0??001?002??????010?00?????00??????0?11?1??????0?????0?????0?010?0??00?01???????010?0???000??0?????????10?000???000?????0????????????00?01????0????0??00??000???0???0????1??00?01?????0??????0????????????0????00?0???0????1??????????0?0??0100??0?1?0111??????0???0??0??0?000???????02?1????????????0?????????10?0????0??0?0??10???0??0???1??0???11000???0????00?0????????0?????????????????????????????0????0??0?00?0???0??0??0?01????0??0?0??00??????????????????0????????????????00?????00?00???????????0??????0?001?01??????0000???000??1?????0???00?0??00?0??????00???00001??????00???0??0001?1?1?00?00??0??0??0????????000011100?????100000?11??0?0011??00?????01?00???0????????????????????0????00?0???0000???000000000??01?00?00???0?0?0????0??00????????

Dilophosaurus

10100001000000101000100010100000000000110020000??0?0?00101100[01]110101100????0???????1010010100100010?0001[12]01000?00?0000111000?10000000002???????0???10000?00001000?0100000001?011000?????00001?01100??0000020011200000000001?00?01021?00000100100??10??0?011100010002000000100000002000003010000?0010??010?001011??0000000001100000100002011001????????[23]?0000000?0?000010010?0100[01]?00?00111111023?1??200100000?011?1100010000001?001010000110010101?020000000010110010211200111100[01]?001211??01100000000000000010?1??1100?0110????????00?000?0010?000?1000000?00?1000?0100??0000000110??0000000?00?0?00111?001?00100000000000000000100??0?0?00000?00?001?11011?0?00010?0000100??20000000?0001100??00??1?1?00011000000000110110??000001??00?0000000???001000?112111??111001110000?011?101111111?10?01000?000000?0000?1?0000?0010100?0?0?00000?00110?1012??00000000?0000?0001100??0111010000000211000000000?000??00000?000011????00000?000??0??010000??000?1?000000?0000??????????000010000000???00??????00???00????000?0?1???11000?10??1110??0?000000?00101000?00001000?010?000???0?00??00112110?10???10010000000000000000?00000000000000?0000?????0???????100000?1?0010??00000??????????1??000000000????10?0000????0?000??000?????000?00?1000?000000000110?0??0000?00000?00000010000000?0?00???0?????0???????0???00000000000?10101000000?00?00?????0?????0????000?000?00??0?????10??0??0111000?????100100000000?????0?000??000??????00??0???00????0??00?????0?????00001000000?0?0??00??0000?00?00?0100001?000?00?0?????????0?0001010000000001000?01000??????0?000000000000000??1?00?0000?0???00000?0?00????0?1?

Dromaeosaurus

?0?0???100?11?01?10?1??0??00?0??1000?111???????????0?10??00?1?1111001101200010??01?1?01?????12000000000010020??000000101[01]10001001000000201???11?10010000100000000?0000000001?11100110001000110011?????????????????????????????????????????????????????????????????????????????????????????????????????????????????????????????????????????????????????????????????????????????????????????????????????????????????????????????????????????????????????????????????????????????????????????????????????????????????????????????????????0??0?0?0??????000000??????????0?0??0????????1??0??01?0?????????0???????0??0?????00?0??????0????0???00????????????01???0?????10????0?0????0?????0???????????????01???0????????????0001???0???0???10???0?00??01???????00????????????????????0?0?00???????????0??0???0??????????000??????????0???0??????0?0?010?0??1????0??????????00??00????1??01?????0???1??????????00101000?0000001??01???????????????????1??0??00??????????????????????????0???1??0???????0?0?0?00?0?????????????????????0?0?????0????????????10???????????????????????1?1?100???0?????????????????????????????000??????????0????2?????????????????0???????0????????????????0???1?????000?1????????????????????0??????????????????????????????????0??0?0??0???0????????????????0?00?????????????????0?????000????????????0000??????????????????????????0??00???11?????000???000????????000???????0000?????0000??0?000????????01???????0?1???0??00000????????????????00?0??????????100????0????1?1???0????????????????????????????????????????1000???0??????000???000????0??0??????0???????????????????

Dubreuillosaurus

?01000010001?0010000110010110001100100121?[12]0????0?????011100?11001?10000200000000000000000010?????????00?000??00000000??100?0?01????0102????????1??10000000000000?010000000?????0101???10???1????21???????????1????????????0????01110?000?0?00?0??1???????1?0?00??????????????0????????????????????????????????????????????????????????????001????????[23]????00???00000010?1?000002?0010???????????????????????????????????????????????????????1?1010???0??0??01?000???????00??????1??????????????????????????????[12]?????????1???????????1??0?00??????00?0000????????????0??0?0??0??11?0????000?????????0???0???0???10??000????????01?????0??0?0????0??0?010???0?1??????0??1?10???0???000????????????????10???1??????????0??1????0???0?1???????00?1?01???????????01????1?????????0?2101?0????????0?11??????0??1???????????0??0??1??0??0???????0???0??012?10???0???????????0?000?1??1?0???????1???0000????011?000?10???000?01??000?0???????????0?????0?000?0????000????0???????????0?10???0000????????0??0????00???0??????0???10?1??????1???0????????????1?????0????????0??0000????????0??0?00?????????????????????????0??0????????0???1????????????????????????0?????0???????0???????0??????00???????????????????????????0???????????00??101???????0????????0??000??0????????????????0???0??0??????????????????????????????????????0???0????????????????????????1?0?00?00?0?????01000?00???1??0??00?0???0??0000?????000???0?00???????00????????????0??0??0???0????????????????00????????????0?0????????001???????1?0??????????00??011?0?????0??????????0??????00???0??00?00?0????00??0????????0???????00????????

Eosinopteryx

110??001?0?1?02???00??????0??????0??????????00??1??0?????0?22???1?????????0??0??????????????????????????????????????????????????????????????????????0010?100102?2????????????????1????????0?????????????????????????????????10?????0???0???????????????0??201??1???10?????1?0??0????????[12]??????00001?0?2?10122??0?0101?0120210??11?1?1???22111??????01?????????31223??????1??0???00?2???????1??200???000?0??11??1????101??0?????1011200?11010?????????????????0????????????2???????????????01[01]????1????011?0?00?2?11??0???1??1100?1100??????0?????0?????1???10?0??11?001??????2???????0?????????????????????????????0?????????0?0??????????0000?0?2?0????????????0??01???1????2??01????0?0??????0?????1?????2?????00?0??0?????0??????????????????????1??????????????1??????????1?????0?????0??????????????????00??????0????1?0????0??10??????????????????????0???0??????????????1??0????????????????????????????????0??????????1??0000???????0?????0???????00???0??0????????????0???2????????????????????????????????????????????0??0?????????????????????0???????????1??????????0?????1??????????????????0?????????????????????0?????0?????????0???????0?????????0???????????0???????2???1?????????????1???????????1???????????????????1???????????0??????????0?????????????????????????????1???0???????????????????????????????0??0???????????????????????1???0????0?????????0?????0????????????0??0?????????????????1??0????????0???00?10????????00????0???????????????0???????0????1?????????????02???11??????????????????????????????????????????????0?00??1????00000???11???0????0???0?????????????????

Epidendrosaurus

?????????0??????????????????????????????????????????????????????11?0???0???0?00101???0????????????0??1????12????????????????????1???????????????????0?????????2??????0[01]21?00???1??0?????0?1?0????0?????????????????0???????0?0?????????10??00????????0???1[12]00?0???11?0????1?10010000?0?01?????000000?012??11100?0?010100110210??10?00000?22001????????3??0?00??[12]12230?????1??1????0?2???1110?0020000?0?010??12?11???0?010?[01]?????1011000???0?0???0????????1???????????????011???????????????0110?1?0??00011?0110?2001101?011?01????0??01?1???1???0?0???????1?10?01?01?0????????0??110??000???????1??????01?????0????00?2?000??000????0???0??0000?0?211????0????0??0??????01????2??01?0??001??????00????1??????000??02??????????0??????????????????????1?0????????????1???????0????????????1????????????????????00?????000???11?00??1??10???????????????????0?101100?????????????????????0????????????0???????????0???000??0?????0???000??0??0?1?0?????0???0?000??0??0????????????0??01???????0?????????0?????????????1?0??????????0??????????????????0?????0??????0????00??????01?0????01??00??1????0?????00??10?????????????????00???0??????????0???????0?00?0????????????????????????????0?????0?????????0?????????????????????0???????????????????0??????????0?????????0?0?0?????0??0?0????1??????????????????????????????????????0??0???0???????????0????????0?????????????????????????????????0?????0?????????0????1???1??????00??????00????????0????????????????????00??????00????1??????????0??????????????????????0?????????????0??????????????????0?00??1????000??0?0????????????????0???????????????

Epidexipteryx

11?2?11??0?2??????110????1??????100????001?00???0??????????????????0???0??01?1??010????????????????????????0????????????????????????????????????????0?01?00110?0???1??????10???0??????????1??????0?????????????????0????????10???????????????????????0?0?[01]200???0??1??????????????0??10?1?????0?000??????????????????????????????????????22??101??????5??0?00??41??30?????0??0???01????010??????0?0??0?0?0???201?0?????10?0??10?1011000?1?1?0????????????????????????????[01]?2???????????????011????0?????11?????????1??????11?10?0?00?0??????????????????0?1??0????????0????????????????????????????0????1??????????????????????01???0?????0???0?????0???????0?????????????????????1?0??0??????????????2????????0??02????0?????0???????????????0????????????1????????1?????????????????????????????????????????0???????0??????0???????1?????????0????????????????????????????????????????????????????????????????????100????????1?????????0???????????0?????0?0???????????????????1??????????0??????????????????????????????0??????????????????????????????????????????0?????100??0??????0000??1????0?0????????????????0??????????0?0??10????????0??0????0?????????0??????????????????????0???0??0???????????????????????????????????????1????????????????????????????0?????????????0??0?0???????????????????????????????????????0?????????????????????????????0??0??00????????00???0?0????????????????????????????000??1??0?0????????????????????1?????????????????????????0???????????????1?????????01???0????????????????????????????0?????????????????????0??1??????000???????????????????????????????????

Erlicosaurus

000001010101????11???0001111101112100000010000011100100100021011110011000000100101010000000?0210??00010010010000000001?101010000?1?1?2?011100111?1010001011000010?1100101101?101000011010002000010?1??????????????????????????????????????????????????????????????????????????00102?0010?0120????????????????????????????????????????????????????????????????????????????????????????????????????????????????????????????????????????????????2????????????????????????????????????????????000?0?0?????0000?0?10??00001[01]000?0?????????????0?010?0????0100000??????0?00?0??0????????10?0??0010?0???????0???0?210??0????00?00?????0??????00010??0??0??????0????0??1?1000???0000???0?1???0??????????0?????2???????00???2??00001???0???0?1??0????000???0????0??0?0??1?????????????1?12?0000?????????0??010???0??1????1?0?000??????01?0???00?????0?0?110002?????????????????10??00????1??10?????0???1?00????0?00010??01?10?000???0?????????????????0??0?00??0???????????????????????????0?0????0?????????0?000??000?????????????10????000???????1?000??????00???????????????????????0????00?0?0???????01????????1???????????000??????????1??0???????????????????0???????0???????0??????????????0???000?0??????????????????????????????????????1????????????????0??0?0??0????????????????????0?00?????????????????111?0????????????????1?00????????????????????????0?0??0??00????????00?0?000?????0??000???????0000?????00?0??0?001????????????????00?0???0??00001??0??????????????0?????????????00????0????021???0??????0??0??????????????????????????????0?00???0??????000???000?1??0??0????0???????????1?????????

Eustreptospondylus

1010000100?1100100001100??11??01???10?1?1??0????????00011?0100?????1??00200?0?00000001000001[01]0??000?0000?????0000000011110011110??01?102????????????0000000000?0??01000000???????1???????????????2?0?000102?110200100001001??0100011?000000000000?10??0?0[12]1110?00?10??????????000020000030100?1???????????????????????????????????????????????????????310000??0?0??0010000000?000?00?0?01110101001010020000000011?10200110000001[01]011100100??012001002110000?0101000102212000111?11?111?????0?[01]000000000011000100????0000?0?0????????0?0110????0?01?000100?10?0?10?????00??000?000??00???000?1?0010?000??1010?001?10??00?0?0000?00?011?000????000?0?1000?0??000?0?0?01??01?10??2?00?0?00?0??0????0?00??101??11??0?000000?00?1??0010011?1?00?000?0?0100?000?10?001??0?1??111?0????2101?1101110?0001?00?100?0???0???10?????000?0?000?0000?000001?10??012?11???0????0??0?0??0?00??01?1??0000?010??????000?01??0?0010???000?11?0??001????????10?00?00?0?000????0???0?0000?????????????0000000?0??0?0???0?00???0000??0000???0000100?0??0???11?0100?????????1?00??0???00?????10??0????0?0?0??0??0??0?1????1??10????????00????????0?01???0?10???????????????????10?00???000????000?0??????0???0??0?000??00????10?0?00????010?00???0?????0?10?010?00?1000000??1??10??0??1?00??000?0?00???0000??0?00?????????????????0??????????001???0???0???0??????????????????0?????100??0?00?0?????010?0000???1?0???0110?010000?000?????00?0?0?00?????????0?0?????0?0?010?????0?????0??000?0000??00????0?????0?00??0??01??0?1??000?110??????1??0000??0110001?1?0010??0???????????000????0000????????00??0?00?????000????000????????

Falcarius

???????????????????????1????10???20???0?0?20???0???????????????????0???0?0001???01?????0?00?1???100?010??????0?01?00000111?0?100?01012?2????????????0000110000210?010??010???????0???????????????0?1?????????011011000?2??1?111111[12]11??[01]???0??????10????0[01]21100000100002001?00001010001030120000001100?10101101011011100020210?111?20102?22112??????01??1?10?0?[12]01211??10?0??0???0001?1011101?0?1100000001?20121111001[02]11?20010010111000100?01201101110?0?0??1?00?01?22110?0100??1?121???000100?000000001010010???11010?01??????????0000101?1???0?0??010?000?1?00?1??101??0??01011?0??0000?00???01?0??001??1???0?2000?0?0?0?000002000??0???0?00?0?210??0?1??0??000???0?1?10???2??0111??000?10?0?0?????2?1??0?0?0??02??????????00?0????????0???????0??1000???????????1????10?0???1????0??11????????00??0?021??000??????00???1??00??0?0110???0???1??????????0?100001????10??????????????11????????0???00??????00001??000??111??0?0000000??0??000?00??0?0???0?000????00??????????010?000????0??0?0???????0?????????????1???????0?0??0????1?0?100001000001???00????0?0???110?????????0????01??????1????110????0???0??0????????0??0??0??????0?0??????0?????0?1????0?1??0????1?0???1????????0???10100?00????0?11001010011?????????????00??????1???0??0???00????0??0?00100???0?00?0001?00?0??0?0????0?????????????1????????????????0??0????0??00?00?????0?????0????????0???????0?????????0????11?????001?0?1000???00????????0????????????001???1000??0?12??0?????0?????0????000?00??????00??0000?0??011?101????0000?1?0?????????0??????????0001?00??0??1000??????0?000000000100??0????00?000???0???00???0000????00??

Gallimimus

0001100001000???01???101100010111?0000?10000000101000001000111111?00??100001100000001000000?02001?000?00200100010??000110111010010101?02???01?100100?1?0??12??????????0010???1010000100??0001000?0?1?010?11?0111010000021011111001101000010?00010010000?0120100000000002001000000000?100200000001010?101???1020010010100100210?110?10001?22000????????40100??01?01210??0010001010?002000111010121100?120100201111011100121110[01]0000121001001001201100212110000101000102211002?010110231211?00?2000120110011001100????1100?11?????????010010101?2001000111101?00?1000?0010?0100000??10?000000?11000??0?0?010101001010000??0?000000?1000?1000?01000??201??110?000?0000000000??0??20021010?0001000??0????0??101011000000000000?1??0010?11??000?0000???0001?0??0?0001??111?00110000?00?0000?0?1100000?0000000?200?100?0?00110?0010001100001000????0??1?002?1?100010100000?????0????0101000000?000??1?000?000?000?0?10000??????01??00100?000??10?00?100?10?000?01000??0?000????????0000??0200?????000?00??00000000????0?001?0???000?0?000??1100?10000000??00??0?0??0?0?00??110?000??00101000010?110?1????1?011100?0?00?0??0?0?000?01?000?10?1011000000000?0??0100000??0?0????1?011???0?0????1??000000010????1?1110?000010?1???????????00???????0?00??0?11002??0?00??00??0??0000000001?0000??0?00??00?????2???????????0?0??????????0100100000?00?00?????0????000?000?0100??00?10?????00?00000?11000??000000?0?000010????00?00?00000??0???000?????0010?000?00?0000???00?0?00?00000000??0?00??0011000?111010??021000101?0?00?00??00???????0?00000?00?00?010000?00??10000000?11000000??1?10??000?00??000??00000????00??

Gansus

?????????????????????????????????????????????????????????????????????????????????????????????????????????????????????????????????????????????????????????????????????????????????????????????????0????????????1?????????????????1?0???10???00?0?0????2?1?1201?111??10102?01?1011101??01010??1100010?1012??11300???210?12101001??12?1?????220001012001180?2?1??34?????????????????11????111?00000010????0????0001[01]0?0??211?0??2??2010201???0??????1????2??11???1????????2?2?2??????????????1211011????1?011?0?00??110100?001???1??001???1????????0?????????0??00?0?1??0?????????????0??1???????10??11????1?????1??1111??????0??20????0???????10?0?????????????????0??????????002??01?0??0?0??1????0?????????00?001??20???????11???????????0???????????1?0??????2???????????????????????????????????????????????????????0????0?????????11??????????????????10??????0??1?????????????????????????????????????????????????????????01011110100?1??0?????????????011????101??????????????01?????????????????0?????????????1????????????0????????1?000?????0?????0?211??????1??????1????1????0???????1????1?????0????????????????1?????????????????????1???????11?0?0???????????????0??????????0???????????????????????????????????????????????????????????0??????????0???????????0???????0?????????1??????????????????????????????????????11?1??10?10?0001?000????????0?????????????????????????????????0?????????1???????????????11??02??????0????????????2???????????????0???????????10101110???????????????0?1??????????????0?????????????0??0?????????????????????1?????1???0?0??????????????0?????????????00??

Garudimimus

0000100001000???01??110?100010111?0000010000000101?00001000010111100100000011000000?0100000?1201110?01002001000??????0??0?1???0?1?1???????????1?1?0?0101??12??????????001000?101100010010000??00?0???0??0?100??10?100???1?111???0110?0000?0?000?0?10??0?01????????????????????????????????????????????????????????????????????????????????????????????4?10?000??????00?0???0???????0?0?01110101211002020000201111011000121110000[01]???????????012011002111000?01010011?2211001?01?110231?1100011000110110011001?0020101?0??110??????????0?10?01?2001?00?1010??????0??10?1??0?0??????1??0??001?11000??0?00?101?1001010??0??0?00?0????000??00???0000?0?1?0?01??000???0?000??01????20?0???0????1?????0000????1?101?0?00??000?00????001??11??0?0?0000???0???????0?0??1??1?1???1100?0?00?00?0???1????0???0?00?0???0?1??00??000???0?00?010000????????0??1?0?2????0?0???????0?0????????0101000??0??00??1??0????0?000100000?????0????0000??????????0?000100?10??00??10000?0??0??????????0?????1?0???????1??01??0????00????00?????????0????000?????0??0????0?????0????0???????????????0??0???10????0?????1?0111??1??????1??00??????0??????????1??1????????0????0???1000????0?0????1??0??????????????0???00010?????0????0??00?1?????????????0??????????????0?????????????????????0?????0???????0??0?0??????????????????????0????????????????10?????????????????????0????0?0??0??00????????00?0?000????????0??0???00?0001?????00000?0?001????????????????1??000?0?????0?????????????????0???????????11000???1?????02????1??????0??????????????????0??????????????0??????0????0??11?0?00??0??1???????0??????????0??????????

Gigantoraptor

0??????????????????????????????????????????????????????????????????????????????????????????????????????????????????????????????????????????????????00?01??12?????????1221?1011?020??????111?11?0?????????????????????????????????????????????????????????2201??1??????????????10?01??0??2?1???0?000????21??1201???01?10?0????????????0???22101????????????????1[23]0120?1?0010000000?0100001?????????????????????????????11?????10?1???????????022?010?1?1?011????0???1??22?0?10??????23???1100?[01]?00?[01]???1????????02??????00?01????????0????0????????0???????0?0?????1??10????????[01]??????0?????0??0???1????????????021?0????0?0????????????????0?0?01??0????0????????0???????1???????1?????0????????????????????0?0???0??????????0?1??????1??????0????????1??????????????0??????????????0???????????????????10???00???????1??0????0??????0??????0?????????????????????????????????????????????????????01????????0????????01???????1???????????00??????000?????0?01????0???????????????0??????????0???????????????????????0???????????????????????????????????0??????0?????0?????????0????0???????1????????????????????????????????????????0??????????????????00??????0?????????????????????????????????????10??????????????????????0????????????????0?????????????????????????????????0??00011????????????????????????????????????????????????????????????????????????????????????1?????0??1?????????0??11?1111?11?1???0?????????????00??????00????????0????????????????????????????0????????????????0??0????????????????????????????????????????????????????0???????????0????????1???????????0?????????????????

Gobipteryx

?01002110110????01??0010111110?01?110?10011000???0?1??????????????????????????????????????????????????????????????????????0?????????????01?0?111????000?1?12??????????020????????0???0????????????????????????1?????????????????????????????????????????????11111??10012111?200?10???010?0???1??0?0??01??1?13????????????????0??10?0?????22?????????117??????????????????????????1?????1???????????????????????????1???1000??20???????????????????????0???????0????1???1?2?2????2??23?????1110011?0?001111?11111?1?0??????1??????????????0????????????????????1?1?????1????????????????1?0????01??1??0???0??0?0????0?0??0??0????????????????1?0??????????????????????????1?01?2??01?????????1???111?????????2??00???0???0?????0?????????0?1?00????????0???0?0????????????????0?12????0??1????????????????????1???????????????0?0????????????????????????????????????0???????????1??1?????011??????????0??0????????0????????????100??00??????00?????0???????0???????00???????????????1???????????????????????????????1??????00?????????????1???????????????0?1???????????????1????1??????0??????????????????????????????????????????0????????????1??????????????????????1????????????????00??????????????????????????1??????????????????????????????????????????0??????????????????????0?????????????????????????????????????????1100???1?0101?????????10???????????????????????0??????????????????????????01???????????????1???????????111???????????1???????????????0???????????1??0?010?1??????????02????????????????0??????????????????0????????????????????????11?0???????????????????????????????????1??

Graciliraptor

?????????????????????????????????????????????????????????????????????????????????????????????????????????????????????????????????????????????????????????0???0200?0?0????????????????????????????????????????????????????????????????????????????????????????????????????????????00??000??????0?0101?012?1112010??0101001112?0??00?11112?22011??????????????????1?4?????0?1??2??0?0???1????????????????????????????????????????????????????????????????????????????1??2??0???0??1?023?2?1??[01]????1?[12]???1111?0??0?????00111??0?????????0????????????0????0??0?????2?1??2?????????[01]???1???????????0?0?00??0???1??0?????0?0???????00?2??????1??0??0?????1????0??????0?????????????2???1?0??0?0??????0??0?????????0?0???????????????????1?????????????????1?0????????????1???????0???????????????????????????0?0???1????????0???1??00?????11????????0??????????1?111???????00?????????????????0??????????1??????????????1???????????????000??????0????????1???01000?????0????????????0??0?????????????????????????????????????????????????????????0??1?????????0??0???0????????????????????????????????????????????0?????????????????0???????????????001??12??????0????????????????????????????1???????????????0?????????????????????????????1???????????????????0????0??????0??0?????????????????1??????????????????????????????????????????????????????????????????0?????????????????????????????????0???????????????????????????????00??????11????????0??????????????????????????????????10?????????0?????????????????????????????????????????????????????????????1?1???0??????????????????????????????????????

Guanlong

1001?0010002000201??11011200??101001001201002121000000011110??111?101000200000??010012000000020?0?0????0110100000??001??100??10?10??0??2????????10??0?000000?0000?0?000???00?12101??????00000??0?0?0????????00?10000?0?2?0??10??0100?0?0???0???10?1??0???0211??00??00??1??1???00002??00?3?????0?0011?0111?011010??010100020210?1111101?1222001????????300?00????0?2100?010??0?0?0??0????111011101121012000?210011111110131100?0?1010100??010012?11012101?00??10????1?221100111??11?2?1?????0110???00?0??11?0110?2[01]?10?00011?????????00??10?00???0?0001000?0??0?10?1?1000??00?01?1?1???0??00??1??00?0?0?011??0?0??[12]??0021?00??00101??0?01??00000?0?[01]1???0101?00??????00??01?0???0?00?00?000????????????1????0?????100?0??10????0?????2???????000????0?1?0??000??1????1??1??00?1?02?00?01?11?????1?1??101???00??00?01?000?0?0101?0??1?011???????001?002??????0?01???????00????????1??0???0??1?????0?????11010?00??0?01??0??????0?0???000??0??000?0???0???10??000?????0????????????0??01??0????0?0???0??00????0???0???????00?01?????0???????????????????0????0??0???0????1????????????0??010?????1????1?????????1???0????0?0???????0??1????????????0???????1?00?0???????0????00??????????1??00??00000????10??000????????00?001??????0???????0??????00??01???0??0??000???00?0??0?0?????0??0?0??000?????????????????????????????????000??0??0???????????????0????0?0?001?11????????00???0?0????????0??00??0??0??0??????000?0?0001??????001?????00000??1000?00010??0??0??????????0100110?????1?00010?1?10?0021??00?????00?00???00?0??????????000????0??0?0??????0??0?00000000000???100??00???0?0?0??100??00????????

Haplocheirus

100?1??100?[01]10200100110111?11000120100?20110002000?00001???010111??010100??0???????1?000?000?10??0?????0?1010???????11??1?????102??????2?????110?10?0010?000[01]0101?00?01?0000?001?100???100000??1?0?????????????1??100??[12]1???0???1100?0?10????1??0?11?0?0?12110000??000?1??1?0??00?2??0??3??10?1?0001??02???1111???1101000[12]0210?11[01]?201?1?22001????????3?0?00????0[12]1???????00?00?0??01??01??01??1?1?0002?0??10??12010??211?010000101010000111012?01?021???00??1?????1???110?2????11??[23]?????00?1??0?0???0??1?0?1??2?????????1?????????01?????01??00?100?001?0??0?1??1?110?1?????11?1???00?10?0?1??0??0?0?????1?0?0?10?100??0???000000????0?000000?0?21?????0??0???002??0????????20??1??0?100????0???????2?1?10?0?0???0?????0????0?????1??0?????00??0?0?1?0???0????????????????0???0????0?0??00?????????0??0200??00?0???0?10??1?020????1100???0??101??02??????010?00?????1?????????1?????????????1????????0??0???0?000?000?????0?10???000???0?11???01?10????0?00??????0???????????00??11?????????1???0??0????????????????0????1?1??1???1?????1???????1??1????00??????????1????????1?0??????1??????????1??????0???0???????0?00??????0??1??1?00??????0????????????0????0????1??10??????0??????0???0???0???????10????1000?0???????????0??????1????????110?02??0??????000??????0????01???????0?0????0?????????????????????????????????0????0??0???????????????0????????00??10????????0??????0????????????0??0??????????????0??????0??????00????????????????????0?????????????????????????????????0????1??0??02?1100?????????????????????????????????????0????????0??0???0000?0000????000?00?1?0???0??????0??????0???

Harpymimus

?000100001000???0???110?1000??1?1?00000[12]0000000101?00101000010?1???01000?00??0000?0?0000000?[01]20???????00?00????????????????????????????????????????1010111020?2????00000?000??010000????000010?1?0?1?0??1?????110?0000020??011000?20??000??00?010????0???121100000?0?00200?0?000000?0100?010000?10100101?10110100?010100110210??10?00001?22000????????40??0??11[01]0121????010?00010?0010001[12]10?0?0110??0?0???20??11?1?1?01?????0???01?[01]???0????120?1???????????????????2??10???01?1102?1211?00??000110110011001100????100001?0?????????10?10??1020??0?0??1??1?????010??01????000?1?110???0?0???100???000001010???10?0?000?00???000?1000???0??0?00????11????0??00??00??00????00??20?01010?002?0???00?????00???0?010???0????00????00???1????????00????0??1?0??0?0??1????1??????001?00??0?0???1??????????0????000??00?????0?0???100001??????0???????0?????????0?01010?0????????????0?0100??????0???0?0?000???000????0????000??????001000000?????????0???0?0???0100?0?0??0??????????000??01????????????????0?????????????????????0????000???????????????000?????0??0???0?????0??????00??????0?0???0?1??????0???00???0??0??????????????0??1????0?00000000??????1?????????0??????????????????????00??0??0?????????0??00001?????????????????????????????0????0???????0????0???0?00??0?01????0??0?0????0?????????????????0????????????0??010??0??????????????????0??????0?00??00????????00???0?0??1?????????0??00?0?0??????????0???000??0???00??????00???0?0000??0?00????????????????????????????11?00?????????02?0????????????????0?????????????1?????????0?00?????1?0??000?11?00000????0???0???0???0?0????0??????????

Hebeiornis

10?0??0????0????0?????????????0?101100?????0?????????????????????????????????????????????????2??????????????????0???????????????????????????????????0010?0012?2?2??0?00?0???????00?0????0?0??????0???????0????1????????????010?????????????????????????0??20111111?1?0????1?210110111010101?110?01011012?11130????2101?2101001??10?0?????22000111[12]11116?10?01????????????????????1???????[01]1???????????20????1??11??????1??????????1?????????0????????1????1????????????1???2????1?????????11100???0??0?111?111???1011010011????????????1??????????0???????0?????0?10?0????1????????1??01?0????????01???0?0?1??0????00?2?0?????20???????????3110?0????????????????0???????1??1?1?011?0??0?0??????10????0?????2000???20???????000??????????????????????1????00????????????????0???2????0????????????????????????00??????0????0???0?????11????????0??????????0??10??0????1???????????????????1?????0??????10???????????00?????0???1??100?000????1?????????????0???????0????????????0??01????????????????00?????????????1????????????0??????????11??????0?????0?????????????????11???1????1???????????????????0??10?????????????????????????????????1????????????0????0?????????????????????0???????????????????????????????????????????????1???????????0??????????0???????????0??????????0?0???????????????????????????????????????????1?01??10100?011?0??0??????????????????????????????????????????????????????????????????1110??1??????111???????????1???????????????????????????1?2??????????????????????1????????????????????????????0??0?????????????????????????????????1????????????????????????????????

Hongshanornis

1012?2???0?0????0????????111????1??1???0???00???1??????????????????0??????0??????????????????2??????????????????????????????????????????????????????0?10??????????????0??????????????????????????0?1????????????????????????10??????????????????1??????0??211??11??1?1????1?1011101??01?1?????0?0101??12??1130????21011[02]111101??11?12????22001?1121111?????????4?????????????????11???????????????????????????0????????10????20?20??[12]0??????0????????????????????????????2?2??????????????12100?????????11?0?0????01101?011?1111??01???????1????0?0?????1?0???0?011??01???????0????0??0??0????????1????0?0?????????00?????????00???????????2100?0????????????????0???????1???021?01?0??0?0??????0????????????000???2????????001??????????????????????1????0???2?????????????0???2????0????????????????????????00??????0????1??00?????11??????????????????10??10??0?????????????????????????????????????1??????????????1????????1???100[01]0?????0?????0???????0???????01??????????????01???????????????????????????????1??????01????0????????11000?????0?????0???1?????????????1????1????010??????????1??????0?????????????????????0???????????????1????????????0???????????????0??????????????????????????????????????????????????????????????????????0??????????0???????????0??????????0?0??????????????????????????????????????????????1??1????????????0????????0????????????????????0????????????0???????????????????????1????????????10100?????????2???????????????????????????1?10??1??0??????????02???1????????????????????????????0???????????????????????????11???0???1???????????1????????????????????

Huaxiagnathus

1001000100?1?0?00?20?10?1[12]00??1?1011001101000000?0????010001?1111??0???????0?0???????????????????????????????????????????????????????????????????????000?00?00?01?0????0?0???????0?????????????????0?0??????1??10?0000??0??000?????????0???10???01??10?00021100001?000??0?1?0000002??0??3?????[01]?002?0011?101101???111000020210???1?2[01]1?1?22011??????01?????????[01]01000???0?0???????0?100?11100?02?101?020010210?11??11?012?000?0?1011100?00100????10??1?????????????????1?001???????121???0?0110?0?0??00011?011002?11100?011??????????10????0???00?00????0?0?10?10?11?000??????0????0??0??000???????0???0100???0???00000????00000010?0???0??0000?0?210???????0???00???1???1?0??2??01?00?000??????0?????1??????0?0??00????1?????0?????????????00???????1?0??0001?1????1???????00?02????0???????????????0????0???00?0????0????1?000001??000???????0??????????001010?0????00????????1?00??????1????????????10??????????0???????????0?00000??0????0?0???0?0???0?000?????0????????????00?01???????0?????0??00????????????00?0????000???0?0????0???????0?????????00?????0????1?????0????0????0100????1????1??????0??????0????0?????????0??1???0??0000000????????????0????0????0?????0????????1??0???0??0??????????????0????????????????????????1???????????0???????0??00????0?????0???????0??0?0???????????????????????????????????????00?????0???????????????0????????00???0????????00???0?0????????????0??0????0???????????????0???0???00???00?00?????1??0?????0??????????????????????0??????0?0????0?1???02???0????0???????????????????????0??????????????????0?00??0??00?0?0???0?????0??????????????????????????

Huoshanornis

101????????0??????????????1????????????????????????????????????????0?????????????????????????????????????????????????????????????????????????????????????????????????????????????????????????????????????????????????????????0?????1????????????0?????????????????????????1?11?1???1?0??1???????00001??2????3?????2?01?2000111??10?1?????220?1?11001115????????4?????????????????11?????????????????????????????????????0?0??21?????12??????0?????????????????0????????1???2???????????????111??????????11?1?0????01??1???1?????????????????????????????1?1???1?0?10??????????????????0???????????0????????????????0??????????2????????????31???0???????????????????????????112??01?0??0?2??????01?????????????????2????????00???????????????????????1?0????????????????????0?????????????????????????????????00??????0????0??????????1??????????????????????1????????????????????????????????????????????????????????????????????0?0?00?????1?????????????0???????0????????????0???1???????????????????????????????????????????????????1?????1???????????0?????????????????11????????0?0??????????1????????????????????????????????????????????1???????0???????????????????1???????????????????????????????????????????????????????????????????????0???????????????????????????????????????????????????????????????????????????????????????????????????????????0??????????????????????????????????????????????????????????????????????111??????????0????????????????????????????1?2???????????????????????????????????????????????????0?????????????????????????????????????????????????????????????????????

Iberomesornis

?????????????????????????????????????????????????????????????????????????????????????????????????????????????????????????????????????????????????????????????????????????????????????????????????0?1??????????11??100???????10??1100???00??00?1?01???1?1?020111????100?21?1?10011??0???01?1?0?0?010???????????????????????????????????????????1?????116?1200???312?3?0????0000??0110???11???????00??????????02?11??????1????????1010??0??00?0???????11????????0????????0?1?[12]???????????????1110???0??2?11100110??101001?211????????????1??????????????????0???2?0??0????????0?0????1??10??????????10???0???1??0????0????0??????0??1?1???0???100?0????????????1???0???????1??1?2??0??????????????01?????????02?0????2?????????????????????????????????????????????????????????????????????1???????????????????1????????0????????0?????11???????????????????0??????0???????????????????????????0????????????????????????????1??00100??????0????1?????????????00?????000??????????????01????????????????????????????????????????????0???????????11?????????????????????????????11????????0???????0???????????0???????????????1?????????????????????1??????????????????????0?1??11??????????1???????????????????????????1??????????????????????????????????????????1???????????0???????0???????????????????????????????????0????????????0??1??1?0??????????0?????????????????????????????????????????????0??????????????????????10?????????111???????????1??????????0????????????????1?2??????????????????????1?0??????????????????????????0????????????????????????0?????0?????1????????????????????????????????

Ichthyornis

201??2100110??????????????1????????????????0???????1???????????????????00?0????11????????????2??1?1?01????????????????11?1??????????1[01]?[02]????????????00101?0010212??0000000?????100?0110??00???0??0?1?0??101??100?0?000?2????????1101?0100????10???0???????211?110??1010200101011101010101011110?01012012?1113000002001120210?1??12?1?????22???1110??118012011??41??30???????0????110???1111000000?0????02??????????0?????????21???10?01?100102200100112??11???0?0101???2?2??????11????????12?0011?0??10011?0??0?????1??????????????????1?0????????0???????0??00?0?1???0???10?00???????10??????10???1???0?0?1?10??2111?2????0??20??0????????31?0???2????0???0???????0????????0?2???1????0?01?1???0?????[12]?1??010001??20???????111?????????11???????????100??????2???????????1??????????0??0??????????1?????1???1?????????????0??1?10???11????????0?????????1????0??0??1?10?????????????????????????????????0??1??1????001??????001101110????100??????0???????011??0?101??????????????01?????????0?????????????????????1???????0?2???????????1?000?1???0???0???211??00??11?????11???1????0???????1????????????????????????????????????????0??1?????1???????0????0????0????1?????0??????????0???????????????????????????????????????????????1??????????????????????0?0?????????????????????00????1????????????????????????????????????1?11?1?010?10??0111??0???????????????????????0?????0??1???????1?0?????????1??????????????111?101?????10????????????2???????????????????00??????1??0101???????????????????????????????????????????????0??0???????????????????????????0????????01???????1??0??????1??????10??

Incisivosaurus

?1120111100110210112110?11101011100000100100001100?0110010021[01]011?0000002????111?1010000000?12111???0?00?00??0?11???01??2????10??1???0??0110?111110?00001110002120?0001211100??0100000001?0?1110??????????????????????????????????????????????????????????????????????????????????????????????????????????????????????????????????????????????????????????????????????????????????????????????????????????????????????????????????????????????????????????????????????????????????????????????????????????????????????????????????????0??0?0???????0000?00??????????0?1???????????1?????100??????????0???0???0???????000?0??????1??????0?10????????????10???0??????00???0?00???0?????0????????????????0???0?????????????00????0?????1???????000???0??????1000??1?????????????0?11?00?0????????00??0?00??0??0?????0???0???????0??1???0??????0?0?11??????????0??????????10????????1???0?????0?????0?????0?001???10??1??000????????????????????????0??1??00?????????????????????????0????????????????0??0????00???????????????0????0?1?????0????????????0?????0??????????????????0????0????0?????????????????????????????0?00?????????0??0?0?????????????????0???????0??????????????????????0???000?1????????????????????0?????????????????1???????????????????0?0??0???0????????????????0101?????????????????????0????????????????0000????????????????????01000?0??00?00????0???00???000?????0??00?0??????0000?000??010????001????????10??????00?1???0???0??1??0?????????????00?0??????????00?????0???0011???1??????0??0????0????????0??????????????????00???0??????000???00???0?0?00????0?????????????????????

Jianchangornis

101????????????????????????????????????????00????????????0????????????????0?????0???????????????????????????????????????????????????????????????????0?10?1000?212??0??0??????????????????????????0?0????????????????????????10??????????????????0??????0??201?111??1?1???01?10?11?2??0101?1???0?010?2012?1?120????010100101101??11?1?0???22?00121100117????1?1???????????????????1??????????????????????????????????????0?01??0???????????????????????????0??????????????2?[12]??????????????1210??????????11????0???????0?0?1?11?????????????1??????0???????0???0?0?10?10???????0????0??1????????1??11???????1????????0?2???????00????????????100?0????0???????????0???0???????02??01?0??0?2??????0?????1??????000???0????????101??????????????????????1?0????????????1???????0????????0????????????????????????0???????0????1??2??????11????????0?????????????0???0??????????????????????????????????????????????????0?1????????1????100????????????0???????0???????0???????????????01?????????????????????????????????????????0??0??????1?????0???????????0??????0??????????11?????0??0????????????1??????0?????????????????????????????????????????????0????0????0?????????????????????????????????????????????????????????????????????????????????0??????????????????????????????????00????1????????????????????????????????????????????????????????????????????????????????????????????????????0???????????????????????1?11?????????10?????????????????????????????????????????????????????????????????1?????????????????????????????????????????????????????????????????????????????0?????????????????0??

Jinfengopteryx

1100???1?000?02?0?00010????1??1?10000??00100??0?0???1?0?????0??1???0?1?0?????0010?0????0000??20???0?????????????????????[01]???????????????????????????00101?0010212?000????0????????1?1??????????0?0??????????????????????????0?????1????????1????01??11?0?[01]21110111?1?0????1?0?0000?0?0??20????0?01010012?101[12]01????1010012?210??11?1?[01]?1?22?1??????????????????31233????1?1?10??000?2?1010??????????????????1??1??????01?????10?20????????????????????????????0??????????012??????0??????????1????[01]?????11???0??????????????11??1????00???????????000???1?0?1?????1??0????????0???????0?100??????????????0???????????????????0000?????????00???????10??????????????????????0??2???1??0?000????????????1????0?0?????0?????0???????????????????0???????1??????0??1?????????????1???????0???????????????????????????1????0????1?0?0????011????????0?????????????101????????????????1?????????0???1?????????0??????????000?????????0??0?00??0??????001???0?????00??????0???????????????01?????????????0????????????????????????0???????0????1?????????????????0????????????0????0?1??0????010?????1????1??????0??????0??????????????0??????????1????0???????????????????????????????????????????????????????????????????????????????????????????????????0??????????????????????????????0??0?????????????????????????????????????????0??????0??????????????????????0?0????????????????????0????????????00???????????????1???1??????0???00???0??00??1?????????????????0??????????????????????1??1???????1??02???11????????????????????????????????????????????????00?????00?0?0????????????????????????????????????

Jixiangornis

1?02?1?1?1?0?0??0?????????11????1?0????0?1000????1??????????????1??0????????????????????????????????????????????????????????????????????????????????0001???2?????????1[12]???10????????????????????????????????????????????????10??????????????????1??????????????111?1?0???01?10011?1??10?00????0?0?01?012???130????21010011?111??10?1?1???220011?1?00015????0???[23]1??30????????????00???????????????????????????0??????????????1?????????????????????????????????????????????1??????????????111[01]0???0??0?011?0?00????1?01?1?1?1????????0????????????0???????0?????0??0?00????????????1??1???????????10?????0?????????10?????????10???????????0?00?0????????????????0???????1????2??01?0??000??????0????????????0?0???2????0???0????????????????????????1????0?0???????????????0????????0????????????????????????00??????0????1?0?0?????11????????0?????????????10???????????????????????????????????????????????????????0?????????????0000?????1?????0?0?????0???????0???????????????01?????????????????????????????????????????????????????????????????????0????????????0????1????1????010?????????????????0?????????????????????0???????????????1????????????0???????????????1??????????????????????????????????????????????????????????????????????0??????????????????????????????????????????????????????????????????????????????????0????????????????????????0?????????????????????????????????0?????????????????????????10?????????110?????????????????????????????????????????????1?????????????0????1????????????????????????????????????????????????????????1????????1???0?????????????????0??????????

Juravenator

1000000100?1?0100?00010?1000??001001000[12]001000010000??01100[01]11?11?0010?0?000?00???0???00000?0??0???????0?00?????????????????????????????????????????0?00?00000101?0?0000????????000????????1??????????????????????00???????000????1????????1????0???10?00?211??00??0????????0?00?01??1??3?????000011???1???1[12]01???110?00?20210?????[12]01?2?22001??????013?[01]?0????00001??????0??0??0?001???11??1??00?00??20????0[01]?11?111???????????????????????0????1011????????10??????????001????????2?????0011????0?00??11?0??1?2011??0?111?0???????00?????01??0??000???0?0?1???0?11?0???????01????0??0??000???????0???010???00??10?10????????000???0?????000?0?0?2?0???????0????0??01??01?0??20?01?20?000??????0?????1??????00????0????00????0?????????????00?0???001?0??000?01????1???????01?10??1?0???1????1??????0?????1??00?01??000???1?0?0??1???0????0???0??????????00?0???0????00????????0??0??????1?????0?????0100?????????0??1????????0???00???0????0???????0?????000?????0????????????00?011????0?0????????00????0???????0??0???10001??000????0???????????0?????00?????0?????0??????????????0100????1????1?0????0??1???0????0?0???????0??0???0?0??????0?????????00??????0???????0???0???????0??00??0????????????????????????0?????????????????????????????0???????0??0?????0???0?0???????0??0?0????0??????????????????????????????????000?0??00?00???????????0????0?0?00??00????????10???0??????????????0????????0??????00????0000??????00???00?000???0??00??0??0?????0????000???0?????0??????0000??1???0?0021??00??????????????0?????????0??0??0?????????????????10??0??00?00000?00?0??00???0??????????0??????????

Khaan

0112011011020???11??111?121011111?000000010000101000110110020[01]11100000000000?10101?100?0000?12011????0?000000???1???????2?0??????????0????????????101?01??12?????????12110100??0200?1???11101010?0?110?1111?0111??1000?20?10101?1?11???1??00000?11??02?0012110010?1100020?1?0000?01?000030???00000000012?1011010??0101000[12]0210??11?10002?2210100?1??014???10?012????0????????????????0?011100000010??0001???01011???0011???10??010111000100?0220?10011?1?10??00??0?1?1?1?011???01?022?21??0010000?0?1?0011?0120?201100000010????????00101??11????1000?0?0?0?00?000110001?????00???10??0??00??0?????0?1?010?1?0??020000??01000000?10?00000??0000?0?21????10??00??00??001001?0?020?01?0010?0??????00?????????0?000??00?0??00???10????11???????001?????01?0?10?0??1????1???????01?01?00?0?1?11???0??0??0000???0?100?10?0000???11000001?0010?????0????0?20???000100000??????????????1?00???0??0???0?0?????0?011??10???????0???????01000001??00??00100?10???0???00??????0???????????????00???????0?????0??0010?00???????01?0?1?000???1010?10?1???????????0?????00?0???0????1?????1?01?0?0??010?????1????1??????0??10??0????0?0?0?????0??0??1100??????0?????011?0??0????0????1??00010??0????1??0???0???1??????110???????????0?????????????????????????1???0?????11???0?????0??0??0???????0?011000??1110?121111111?????????????????????100?0??0??0????????????0????0?0?000?00????1???01???000??1??0??0???0?1111010101010?0110?00000??????00???0??00??1?01?000?0?????0??0????000???00????00???01000???1??10??0110?00?1????0??0???0??????????00?0?????????????????????00000?1100010??0??1???????0???011???0???????????

Limusaurus

0010?00101?00???0???010111110?001?0010000110000000??0000?0?2020101?0100????????10???0??0?0000???0?0????0???0??????????????????????????????????1?????0001??12?????????02??010??10?0??????1?0?0????0?1?0?00?1?0?001?001?0?0??00111?02??0?0??10?1??00??????010110?001?20??20?1????0??1??1004000??01?010???0???120?0?????0???0?01011000100??0[01]20?01??00001???0??????0?1001?0?????1010??0?0??111?1023010??001010?001111112?012?001?0?0010?00??01??[01]01010???1??0???10?0????2????01????0?0?1?????001[12]0?0?0??20?11?1?11?2?11??0?0?11????????0???????0?????00????0?1?00?10?01??100????01??01???0??0??????0????0??10?0?01???0?0???000??1???0???0?????10?0?0?00?????0?100??10???0?1?1????20??0??0??0?????1????????01????0?0???1????00????01???1??????????0??????0?0??0????1????1???????0????????0??01?1?1???????0????0??0?0??????01?????000??1???0???????1???????????0?10?000?????????????112?0?0?????????100???????00????0??????0????????1?0??0???0??1?0?????10????0?00???0??0????????????0????1??????1?0??????0?0??0????????0001?????????1???1?????????????????????1??????0???11???0?1????0?0????0?????1???????????????0?????????0???0??01??0????0???????0???????10???1????????????01??????????1??0????????????1???1??????0????0???????????????????0?????0???1??????????0?????0??1????0?????0??0?0???????????????????????????????0?0????11??????0??????????????????????0?00??00????????00???000????????????0?????????????????0?0?00010??????????000??0????0?010??????????0????0???00??????0?????1??0???11??0??021??0????0???????????????????????00????1?00010????????00??01?1100?00?????????????0???0?????????????????

Longicrusavis

1012?[12]0?0010????0?????0??11??????001???0?0?00????1?????????????????0??????0?????????????????????????????????????????????????????????????????????????0010????0?????????00????????0????????????????0???????????11??????????????0??1?1???????????0???????????211??11????10???1?10?1??00?0101??10?0?0101?012?1??30????[02]10110111101??11?12????2200011?20011??????????????????????????????????????0?????????????????????????21??????0??????00???0?0?????????????????0??1?1???2?1?2??????0???????1211?1?????11011???0???011100?011??11?1?0??0?1??????????0?????1?0????????1?11???????0????0??00???????0??11???????????????1??????????0????????????2100?0????????????????0??????????002?001?0??0?0??????00??????????10001??00???????001???????????0??????????101????????????1???????????2???????0???1??????????0?2????00??????0????0?????????1???????????????????????1??????0??????????????????????????????????1??????????????1????????1?1111?00???????????????????0???????01??????????00??02???????????????????????????????????????0?1??0??????1?1?000?????????????1?????????0?????11???1????0????????????1??????0?????????????????????????1???????????1???????0????0????0??????????????????????????????????????????????????????????????????????????????1??0??????????0?0???????????????????????????????????????????????????????????????1??1??1?01?01??00111???????????0??????????????????????????????????????????????????????????111???1?????110????????????????????????????????????????????11?????????????0????1?????????????????????????????????????????????????????????????0???????????????1????????????????11??

Longipteryx

201???1??0?0?0200??0?????2??????1??????0????0????????????????????????????????????????????????1??????????????????????????????????????????????????????0011?002?0?????0???0?????????????????????????0????????????1???10???????010??????????????????11?????0??20111????1?0???11??0011011?1?0101???0?01011012?111[23]0????210102?01111??11?02????2200111121?116?????1??4?2??0????????????11?????1????????????????????2????????211?0??20?[12]?10120?100???????????????????0????1?????211??????????????111[12]01??0??0?011?01?0??101??????1?11?????1???0??????????0?????1?0???2?1?10?0????????0???????10?0????0????????0?0?1??0????00?2?0?????10???????????2100??????????????????????????1??110???1?0??0?0??????0?0???0??????0?0???20?????????0???????????1??0???????1??????????????????????????2????0????????????????????????00??????0????0?0?0?????11??????????????????????1???0??0??????????????1??????1?????????????????????????00?????????1??100?0?????01?????????????0???????0???????????????0????????????????????????????????1??????0??0?????????????????????0?????0?????????????????1????1????1?1??????????????????????????????????????????1????????????1???????0????0????????????????1?????????1???????????????????????????????????????????????????????????0???????0?????????????????????????0?????????????????????????????????????????11??1?111?10?0??11??0100????????0?????????????00?????0????????????0????????????????????????110?????????11100????????01????????????????????????????????010????????????02???1????????????????????????????????????????????????????????11???0???1??????????????0?????????????????

Longirostravis

201???0??0?0?12?0??1????????????1??1????????????????????????????????????????????????????????????????????????????????????????????????????????????????0011?1020?2????0?????????????????????????????0?????????????????????????????0????????????????1??????0???01??1???1?0????1?1001101??11010????0?010??012???1[23]1???????11??????1??1??0??????????11121111[56]??0?0???4???30????????????11?????1110????10?????0????12?11????????????????????20??????2?0???????????????????1???1?2?2??????????????1111????0????011?1110???01????????111????1??????????????0?????1?0???1?0?????1???????0???????1????????1??0????0?0?1??0????00?2????????00??????????3??0?????????????????????????????122???1?????????????00????0????????0???2????????000??????????????0????????1?????????????????????????2????0?????????????????????????????????????????0?????11????????????????????????????????????????????1???????????????????1????????????00?????????1????0?010???01?????0???????0???????0???????????????0????????????????????????????????1??????0?????0??????????????????????????????????????????11???1????1?1???????????????????????????????????????????????????????1?????????????????????????????1?????????1?????????????????????????????????1?????????????????????????0??????????0????????????????????????????????????????????????????????????????11????11??10?0??0??????0????????1??????????????0?????0?????????????????????????????????????1???????????110???????????1?????????????????????????????2???1?????????????02???1????????????????????????????????????????????????????????11???0????????????????????????????????????

Mahakala

???????????????????????????????????????????????????????????????????????00?00?????0????????????????1??????????0?01????01121?????????01???????????????????1???1?2?????????????????????????????????????????1?1??????????????????????????????????0????????????2?1???????????????????????????[34]????10?0?0?101???0????????????????????????1???????1?[12]????????4??00??1??1132???10??0?0??0?0?1??01110000?1??0?020101?10?111100???????????????????????11?001011????????0?????1???1?112?0????023???1??012001?0??0101??????0?????1?1[12]??0??????????0???????????????0?????????2?????????????????????0?0??????????0???01????????201????0?0??0??????0??0??????0?0??10??01??00????????????1??????????????????????0??????????????????11?????????????????????????????0?????????????????1????????????????????1?????????????????????????????????????01?0?0?????????????????????????????????????????????????????????????1?1??????????????????????????1????????0??00??01????0?0?????0??????????????????????1???????????????????????????????????????????????????1?????????????0???????????????????????0????0??????????1???????????????????????????0??????????0??????????000100??01????1???????????????????????1??????????0?????????????00???????????????0???????1???????????????????????????????????????????????????????????????????????????????????????????????????????????????????????????????????????????????????????0??????0????????????????????????????01???????????????????????????????????????????????1?1????????0????????????????????????0???????????????????????????????????0???????0?0???????????????????0?????????????????

Majungasaurus

1000000100020001020010000001002100[01]01100101211210000111111121010110110002010?01112210001012010000?0110?11000011000000011100?010000??0100??00011000010000100002000?0000101000?10010100001100000010010000111200102201000110111001100211101001101010010?00011010000010000010?100?1020200100410000110040???00??00100??00?0??0?00?00000011?0?012?????????01310111010[01]00000100010000000?00100?111110[12]3110010211000000110111???????????????????????01?1010011?0000?011110011?2110001100111111211?00?[01]000001000010001100????010001?1????????0?00?1?00010?00?000001100???01010?0??0000?1211100000011011?0?0?100??1001?0010?0?0010000??1000?1011000002?00?00001?00?0?110101000?00?0?00??200000?0??02?1?0?00?001011000110?00??00?000011??010?011?10?0?00001110000011?0000111?111?????00?1011110?01111??0000000000??0?00?0??0?00000?00000000001?000??0?000?01?102?00???010?0?01??000100???0111000000100011020100000??1111?1?000000000000101100?00???1??01?011000100??011000?1111??????????000001011000??0?0?00??11021011111101?1011???10000?000??1110?0?000000?00?01001??000?0001?110111??000001000100110?1001010?1111101?000111011100011110110000?0000?????0?00110?100001?1000???101001??0000????1??00000?0000???10?0100????110?01??01?????0000?00000100010101010?001??1100?00??0?11000001000000?0?00????00??0????????0??111111101101101000000000?00????00??0????00????0001100?00?0??100010?10100?110?0??11?0011011000010???00?00000000??0?????0?000???0?0?0?011000000?0?0?0?000000000?0?00?01??0000?00?01?111?0011010001?1?000?0???00000001?01001000010?110011?111?1??00?001000000000?01?00?0?0011???00010??010?001001?

Masiakasaurus

??0?????00?????????[01]01?1???00020?000?00?0??0??????????0????????????????020000???00?????00000????0???110????????00?????11?000?1?????0?1?2????????????0000100010[01]00?00011000?0????10?0???01????????010?001112001?12?1010?10101?1?1?0[12]111?00??0?0?1??10????0???100101120??00?100?10001??100[12]100???1???????????????????????????????????????????001????????411001010[01]0?000110000??0000?0001??1?1?1023?1??100???0001?11?11100110000000?0?0?01000[12]01101010011101001010110011221100[01]?110011011211??0?110000?0?0010011?10??????0????1????????0???00??0???0?????10??1??0?10?????0????00?2?????0????0000?0001?10?0?1010??01?10???0?1??0?1?01110??00??????00010010?1???100110??0?00???01?????0??????1?11???00?0?0?1010?11??000??00???0?1??0?0?01[01]????0????00?0??0??01??1??1???111?101100?????000?011011?01?00?1????000?????????????1??0???000?1001??00?0??10????2?000??????0??10???1???0??01?1??0000?100??10000?010?????0?1????010000?0??????0??????10011??0?0?010????1??00?00?00?????????0???1?11???0???00?????1100?00010101??1001???0??1???????1010?0??0000????1?100??100???0001111001????00?01?01????0?1?????1??111011????0?1????0?10110001000???????????????????10?0????000?0??01?01??????????1???0000??01?00010?0?0?0???1??0?0?????????0????0??000????0??11??1??01?01?0?00?1?01?0011???0000??0?10?????????????????0???01?11010001??100?????00???????????0????00??????01???????0?????000?0?00??11???????????1011??0010?????000??????????????????????????0???1?????0?0?????000??01??????0?01??0000?0??011?1???011?1?????01????????01?0??011??0010000101?10??00??0???000?00?000?00???????0??0?00?0???0?0????????011??10

Mei

0?11100100?1??200?0???0?1111??100111?0??01?0000?11?0110000?01111110011?000?0?0010101??00?????2001????10120100?0?????????[01]???????????????????????????001[02]?00010212??00?1000????11?11??????00?1????0???0????2?01010?1000?21??010??1?100??????1010??11110?0012111?11??100020?1?10?0??0??0??3?1???00000??012????[12]01???01??00?2??????10?0?????220????????013??20001?[12]12220??11?1??0??00001?1?10100003000???2?111?1?0111????01??[01]???0?10?0211?1001??2001011????1?0?0?0???1??22?0?2?0?????231???1?01100?11?????11?01?012??11??11?1?????????1?01???1????????0?0?1?0?1???2??10??1??????0???11??0?10??????????????10?0?????200?02????0?0?00?0?0??01?0?000?1??1????????00???0????1??1????2??01??0??????????0?????????????1????1????0?????0?????????????00??????1?????0?0??1????1?????1?01?02????0???1??????????0????2?0?1??1?????0??????0001???0110???????0????????????1101??0???1????????01??0???0??1???????????01?011???0????00???1???00000??????0?0010??0????0?????000??0??0???????????1???02?????????1??????000????????????1??????0?????1?01???1??0???01???????0?0?1???????????????0?01?0?0???10?????1???????????0???????????0???1??0???2?1?110??0100000001002010???01?0??????0?????1??1????????????0??????????????00?00???1?0?????????????????10????????????1????????0????????0???0??????0??0?0????1????????????????????????00???0????0????0????????????????????????0?0?????????????00?????0??1?????????0??0????????????1??0?1?00???0????????010??0????21?00???????????????0?1???0?????0?????1?011??1????00021??11??????0?????0?????????????00?????????????0?????000?11?000000001?0?1?0?0????0???0?0????0??????????

Meleagris

1112?21?01100???01?????0?21010211?001010011000001001?10100022??1??00???010001101110???00?000120?1?100110??1?10011???01??2101?00?????????01100?11????0001??12???????????200???1?0?0??1100?00??1?0?0111010001??100010000021010111011111110??0?????1101?2?1?020111111110102?010201110101110101111000101?012?11130100?01?1120011?1???1?1?????22???121101118012?011?41????001??010?0?011????1111000?10101??00????01112100?1010?01021?21101010100102200100112??210000001011??2?112?0??11????2??0121000011??11011?0?101?11110?0011011111001???1?0?11??0??0?0??1100??0000?100?10??10?00???11??101?????20??01?0?01011010??21120??0??0?020????0?1001?3100?0?2?????????0????0???0?0?1???021001??????21?1????0??????1??21??01?0?????0??1110??????1??110??0???????0?10?0?0?2?11??1?0?1110?0??0?0?001?01?010001??10??0?1??11??1??00?0??000?0101?10?110?????0??000?????110?11??00??10????????????011000?????????????????011????1?????1??11????111111000111?01??0??010?????011??0010110021021??10??01???????00000??0?0?1??00???????01?0???001?21?001?11?1?11000?10?0?0???00?211???0??11???0?11???111??010011??1??1110???000??10000????0?0?1?????10??001110?1???111111???11?0?0?11?0?0????1???100?0???????0???????0??00??1100?00?0???00???01???????????????01????????1??????1???12?0??0??0????01????0???100???1??11121111111?????????10?????????01111???11010000?01111??00?????1?1???00?1?????10001?000??1??????11?0??????0211??????????1?011111102?????110101????2?001???????0??11???1?1??0??????1010?01?????????????0211?110??1??0??????0????????01?0?1??0??0?????0???????????011?11?1?0???00?010?????01??0?????1100????11??

Microraptor

100000010000?02?0?00?101?001??1???1000?001?000??0??0??01?0?1??0??????????????????????????????????????1??????????????????????????????????????????????0002?000[01]0[12]11?000??001???????01??????????????0?????????????10?100??[12]???01??0011???00???0010?11?1???001[12]0110111110??2??1?10011000000020???00001000012?101201???010100111210??11?12110?221?110100001[34]????001?312430?????1??20?000020101010000010000120111?001?21?10?21[01]?0101001010211?1001?0[12]011011?0??1???000???1???1?01[12]?0??1?02312?1110100001101000110211010011[01][01]1110101111111110?11??0????0?0?????1?0?11?02?11?001??????01???1??0??0?1??00???0?0?010?1??0??20000[02]?0?0101001?001???1?00000?1?2110???0?0???000???0???1????10?11?0??000??????00????1????0?110??02?0??00??00?0????1???????00????0?01?0????0???????1???????10?1?????0???11????????0???0?2?0?000?1????00??0110?0??10?111???0???0?????????011111?00?????0????????11?0???????0????0????????0?????????000???1???0010?000000000001?0???0?0???01000??0??00???????????0??02?0?????000??????00?????????0???1?0????00????0?00???????00??1?????????0??0???0???110????11?0??????0100??0?1????1?0????0??10??0??0???????????00?1?0?0??0100110?01002010?0?00?00?????0???0??0???????1???1??0??0???????1?0????00?????0??????????0??????1???????????0???????0??0?0???????0?0?01????0??0??????1???1?????????????????????????????00???0??0??00???????????0????1???0????0?????????0??????????????????0?100?????1?????00?0??000?0?00??00?1?10?110??0?2?000??????????0????0?1?00??????00??0010?0???1??10???2?0?1????????????0?0?????????????0??0??0???0?0??????0?001010000001000?1??0??00???????0??????0??????????

Microvenator

????????????????????????????????????????????????????????????????????????????????????????????????????????????????????????????????????????????????????10011?12?????????02?10?0?????????????????????0???0111?1?01?10?100?02?01????00?21110000?0000???11????0??????????1000200100000101000002011000?000?????1??11???????0??????????????????????10[12]????????????0???1?0?????000?00?0?0???000?01110000?1??000201??2??1???11?0011?[12]1010?10??????????12?01100110001000000?001?2211??[12]?01?1?0231??1???1????????????????????????????????????????0?0?0??????01????????0???????????0???000?????????00???????0?0?00??01?11??00?200???????????0?10?0??????????????10??????0?0?0??????0???1?????????0???0??????????????????0???0??????????????0????1??????????????0?0??00?????????1?1?0??00??????????0??111??0?????0???0?2???????????????00?1?0???00?11??????????????0??????????0?0???????????0????????0?0?0??????00??????????????????01?????00?00?????????00?10???000????10?01?0?00??????????00??????????????0?????0???????????0???0?????????0??????10???0?00001???????0????0???????1?0???????1?0????0????????????????0?????????0????????0??1??????????????????0000???010?????????????1???0??????????1???0?0???????????????00000?0??????????????0?????????????0?00??????????0???????????1??0?????????010?????1?0???111??1??????????????????0???????0?????0????????????????????????????????????0????????1??????????0111???[01]10?????????????????????00?????????????2?????????????????????0?????????0????0????????0?1???0?????????0????????????????????0??0??0????????0??????0?00???????000???0???1?????????????????????????0???

Monolophosaurus

100000010001100?0000?10011110000100100020120212000001011011101101111100020?0?10002010100101?000100010?00200001?00??1111?2????10?10???0?????1???????1000[02]000000000?0000000001???100110?01000010?011?01001111100011?1?0?02001??01001110001000001010010??0?0[12]????????????????????????????????????????????????????????????????????????????????????????????300000????????0110?1??0??????0?1?0111111101100202100?10001101010011?00000100101000?0?0??????????????????????????????????????????????????????????????????????????????????????????0??0?00??00??0100000???0?1?0??1?00????0?????1?????0000?1???????0??100??0?00?????0??000?0??0?0?0000??0???????0????11?0?10??????00??0?00???0?????0?????1??????????1????1?????00??00000?1??0?????1?1??0??100?????0?????0011?1??1???????00?1?12?01?0?0?11???00010?0???0??1?0??0?1000??????01??000?0??????1?0?01?012??????0????0?????00?0????011?000?????1???0?00????010100?1000??0000?1???000?????????0??????0?100??00?????????????????????????00???1000??10??0101?0?0??00????00????????00????1?0?????0??????0?????1?????????0??????1???0????0???0????0011??1?????1????????????1??0?00000??1?????1????0?????????????????0???????01??00??0????0??????1??0???000?0??????????0??0?0?0?00??0??????00??0?00?0??0????????1??00??0?0??0???0????0?0?????00??0?0??0???????????????????0?000??001???????00000??????????????????????00?00?0??00???????010?1?000??1????????0?0100?0000??????010??0?000???????????????????100?0??00000??00?0???????????0???0?????????0000??0???0011???0??????0??0?????00010?1???00?0???0?0?????0?0????0?0?????000??00???1????0????0???????????10????????

Mononykus

??????????????????????????????????????????????????????????????????????????????????????????????????????????????????????11[01]??0?????????????????????????????1????20????0????????????????????????????2????????[01]?01?[01]01100002?11010001100?000100001000001?0??1[01]2110000?010000001000001120000140101020002??011??0?010011111?110????1????????????????100000????2?0???????????0000?1100?0??01??01?1010000???000020?2?????????1?????1????21??????0???0220010011000110?0001101022222?2001011?13121?01012000120110011001100?01111000110???????????11?1??????11???????0?????0?01?1????10?00?1??0??001?????0??100???0???1??0??2111???0??0??01?00?????0????1210??1???????0?0?0?0?????0?1????2?021?0??1??1?00100000????1??110?000??0???????10?010?1????00????????????000?????0?????1?0010100???????????0???0??????????0?2????0???????00?00?1?21?0?0?00??????????????????00??00??0?????1?????????1???00102?2?????????????????????????????????000?0??1?00???000?00????????001??????000?????????10???1??????????1?????0?0??????????0??1?0?????0?00?????11???10?00?10101???11??000??0?0?10?????10?0?0???10?????0???????0?10001?01?0?0??????????????00??0??0?0010?000000000000?0?0??0???????????????????????????0?0?1?????????00100?001?????????????????????1???????????02?????????0??0???0??0?0?01??????????????00?0???1?10?0??????????????????0???????0??00000?????0??????????????????????0?????????1?????????????1???0000????1????????0??????110????11???????????????0????????????????000?00??????00[34]?1010????0??????????11??0??0?????????0??????????00?1??0?00?????0??????1????00?????????0?0??????111??0??00????00??????11??

Neuquenraptor

????????????????????????????????????????????????????????????????????????????????????????????????????????????????????????????????????????????????????????????????????????????????????????????????????????????????????????????????????????????????????????????????????????????????????????????????????????????????????????????????????????????????????????????????????????????????????????????????????????????????????????????????????????????1?????0?1????????????????2??10?????????2312?1??01?000?1?11101110??01?01101011??0????????????????????????????????????0??1???????????1???0?????????????0?00??0????????????????0???????????????1????00?1????????????????0????????????2??0?????????????100?0??????????1????????????????????1???????????????????????????????????????????????????????????????????????????????????????????0?????????????????????????????????????????????????1???????????????????????????????????????????????????????????0?????????????0????????????????????????1????????????????????????????????????????????0???????????????????????????0????????????????????????????????????????????0??????????????????????0????????????????????????????0????????????????????????0???1????1??????????????????????????????????????????????????????????????????????????0???????????????????????????????????????????????????????????????????????????????????????????????????????????????????????????????????????????????????????????????????????????????????????????????????????????1?????????????????????????????????0?????????????????????????????????????0??????????0????????????????0?????????????????

Nothronychus_graffami

?????????????????????????????????????????????????????????????????????????????????????????????????????????????????????????????????????????????????????????????????????????????????????????????????0?1?????????????100????????00?11?1??0??01?000??0????????221100????10??1??1?0?0010200010[23]012?00?001????????10010??001100020210??10?1?0???22?02??????014??01001?[23]0[01]????????0000?00?110??01???????2????01?2?20?2?0?????0111?21??0?2110001?110?022201001????????00000?????1???0?????1?1[23]?????00000?0?00000011000?002000010??11?????????00????????2?0?0???????1?00?00?0??0?1???0????0??0??0?????????0??0???????2?????20?0???0?????00?2?0????????000?0?200?????????111????0?0?0????1?0?1?1??10???????00??????0???1000??0??1?????????0???1????????????????11?0????????????1????001?????????????11????????1???1?1??0?00??????0????1??10?1?0?001??????????????????0??0?000????????????????0????1?????????????????????????????????????0?1????0?????1??0??????10???0?00??????0???????????????00????????0????????????????????????0??????????0????????????????????????0??????0???11?????????????????????0?0????1??????1??1???0??????????????????0???????????0???????1????0????????????????????????0?????0???????????1?0??0?0201?????????????00???????0??????????0??????????0???????????0???????0???????????????????????????????????????????0????0???????????????????????????0???????????????????????1?????????0??1???????????????0????????????00????1000????1???0??????????????????????????????????0???????0?????????????????????????0????????????????????????????????0??0??00???100???????????????????0?????????????????

Nqwebasaurus

????????????????????01?1???010101?000?1?0??0??????????0?????1??????0???01?01?001?00???????0?????????1????????????????1??0?0?????????1????????????????????1??1020????0????????????????????????????0????????1??1?10?10000[12]1?10?01?11?0?0?????1???????????0??211000000[01]?0000?11000000[12]??0?030????0?00100001?1?100110110100?1[12]0[12]?0??00?20[01]???22000????????????????????????????????????????????????????????????????????????01[01]?[01]??1??????????????02200100110???0???01?????2?1?011?1??1102312?1?001000010010001101110??0?1100??11??????????11???1?1???0110??0???0?????0?11?1??0?????10???0??0?10?????0???0???0??????0???0?1?2?0?????001200?0????10?00?0????0???0?000??00???????1????2??21?1??[01]????????00?????????0?000???0???????????????1?????????????????0?0????????????1?????1?0?????????????1???1??????0????0???0??1????00???1??00?????00????????????????????0001???????1??????????????????0?2???0??????????0??????????????????0?0000000?????0?01??1???????010???????0????????????0??01?0???????????????0?????????0???1?0??????????0???1??0???????????0?????00?0???1???????????????0????01??11???????1???11?1???0???????????0?????0?????10?0??????0???????1????0?????????0??1??????0???????????????0???????10???????0???????????????0?????1???????????0?????????00????????0??0?00??????????????0?????????????????????????????????0???00??0???????????????0????????0?????????????0??0?????0??????????0??????????????????0????????????0[01]??????001???????0???????????????????0??0?????0?????10111??1???01??2?00???1???????????00??????0?0???0???????????00?????01???000?10?????0?0?????00?????????????0???????????

Ornitholestes

0000010100?1?0210?1111011210?01010010011010??00?00?001011?0[01]12111100000?0????0100?010??000000200010???002?000???0???01??1?0??10???0?1?????????1?1???0000000100[12]01?0000000001?111300????11?001??110???????????1120001000?101????01100?0?000000001??11????01?1??????????????????00001000003011??????1??????????01?00010???1??????????????????000????????30100???0?0021???0010?10??0?001?10111010100101012010?2100110111001??0100001010100100100???????110???0?0??0????????????????1??????????01[01]0?0?00000010101?0?????1?0??1?0????????00?010001?20???00?000?0??0?10???1100??1????0?11???0?1000?10?1????0?010?1?0?0????0000000000?011011000??0??00?0?1110?0???000??000?0???0??0??20?0?0?0?00??0????0?????1????1?000?000?0??00????00????1?11?0??00??????0??0??00?101??1?1?????000??01?01?0001110?00????0?00??201?000?1???0?0????000000100100???0???0???02????0?0?????????0???????[12]0?01?0???0??0???1?001?0?0000000000???0000????0100????0????00?????010?0?0???0?000?????0??????????10?0?0100?????000???0??00????0????0?????????10?10?000?????0??00000?00?01??0????0???????110??0????0???0??0?00??0?1?1??1???????????0?0??0?0?0?0?????00?00???0?0000000???00001?0??????00????0??0???????????1??000?00000????1?0??00010000???0???????0?0????????0?????0??????????000????0???0?????0???????0??0?0?????0?1???110100?????0????????1???0???00??0??????????????????0????0?0??00?0?1???????00???000?1?????????0??00000??00?????00???0?001??0???????0?????0?1001100?????0?????0??????????0??0????????0??00?????1??0011???0?????????????0?00?10????00?0?00?0??0????0???????0?0???00000000???0????0????0???0?0????0??????????

Ornithomimus

0001100001000???01??1100100010111?0000110000000001?0000100011011110011100001100001020000000?0200100????02101000????????10111010?1?101??2?????110??00010[01]??12?????????0001000??011000100?00001000?0?1?01???1?0111010000021011111001101000010?00010010000?0120100000000002001000000000?100200000001010?101???1020010010100100211?110?10002?22000????????40100??01?01210??0010001010?002000111010121100?12010020111101110012111010?00121001001001201100212110000101000102211002?010110231211?00?2000120110011001100????1100?11??1??????010010100?2001000110101?00?1000?0010?0100000??10?000000?11001??0?0?01010?001010000??0?000000?1000?1000?01000??201??110?000?0000000?00??0??20021010?0001000??0????0??101011000000000000?1??0010?11??000?0000???0001?0??0?0001??111?00110000?00?0000?0?1100000?0000000?200?100?0?00110???1000110100100?????0??1?0?2???100010?000????????????0101?00000?000????00??0?0?0001000?00????0??01??00100?000?????00?100?10?000?010000????00?????????001??02?0???0?000?0011?00????0????????1?0???00??0?1000?11?1??00000000?000???00?0?0?0???110??0?0?0010100?010?110?1?0111?011100???0??0??0?0?000?????00?1???01100000000000??01000?0??000???????01??????????1??00??00010????1?1?10?00?0???1???????????0????????0?????0?1?002?????????00?0??0??00?0?01????0??0?00???0?????????????????0????????????010010000??00?0????????????0????0?0?00??00??0?????00?00000?1???0??00000?????0?010????00?00?00000??0???001?????001?1000?00?0000???0??0????00000000????00??0011000??11010??021000101?0??0??0???0??????????00?0?00?0???10000??0??10000000?110000000?1?100?0???0???000???00??????0???

Oviraptor

?1?????[01]2???????????1?0????011111?0????00??02?2???????01?00102111??000????????11???????0??????0??????????????????????????????????1???1??????????0?101?0???12?????????1211010???0?00?1????11?1??????1????????????????????????????1????????????????????0????211?????????????????????1???0?????????0?00??12???1201???0101?0120?10??11?100?1?22102???????1???????????????????????????????????????????????????????????????????????????????????????????????????????????????????????????????????????????????????????????????????????????????0???0?1??????0???????0???????1?00??????????????????????????????????????????0???0????2????00???????????0????????????????0?????0??????????????01?0??0?0???????????????????0?0???????0?01????????????1???0??0??????1?0??0?0??1????????????1???1??0?0?1???????????????????0??0??00????0???1100?0????11????????????????????0?00??0????????????????????????????0???????0?????????1?????0???1????0???00??????????????????????0???????????????????10??0??????????0???0??0?????????????????????0????1?1???????????????????????00?????0??????????1???????????0?????????????????????????????0?????????0??0??110?0100000000??0010???0????0?????????11???0???????0????????????????0???????????0?????????????????????????????0??????????0????????????????????????000??1??????????????????????????????????1???????????????????????????????0??????????????1?????0????????????0?11??0001?1000?010??????1??0???00???0100001??????00???????????????????????????????????????????????01???????????????????????????????????????????????????0????????11???10??????00?????????01????????????????

Parvicursorinae_indet

????????????????????????????10?????????????0????????????????????????????????????????????????????????????????????????????????????????????????1???????0??????????????????00?????????0??????????????2?????????????0?1?0?????????????11??????????1????0???????2??????????????????????????????????????????01????101?011?11?11????11???????0???220?0??????????2??????11?2????00?0110??000?1?????????????????0??????01????0????0?0??????1????????????????????????1??0?????????2?2???0?11??13?????001?0?0?2??10011???0???01?00100110???????????0?01??????01?????????????2??1?1?????????????0???????????0?1?0??????????????1?1???0?????0??0??????0????02?0???0????????????0???????1????2??01???????????2?00????????????1???????????????0????1????????????????????????????????1???????0?????????????????????????????????0???????0????1???1?????????????????????0???????????????????????????????????1???1??????0????????????????????????????????????????0????????????1?0???0?????????????????0?2?????????1??????????????????????????????????0????????????????0?????11???0???????????0???????????1?1????????????0????00?00??????????????????0???????????????????????1??0?0????0???????????????????????????????????0??????????????????????????????????????????1??02???????????????????????01?????????0????0?????2????????0???????????????????????????????????????????????????????????????????????????1?????????????????0????????????????????????1????????????????0???????????????????0??????????????10????????????0???1????????????????????????????????????????????????1?01????????00????0??????111?????0?????????????1???

Patagonykus

?????????????????????????????????????????????????????????????????????????????????????????????????????????????????????????????????????????????????????????????????????????????????????????????????0???????????????????????1?????0???0?0?01????101??11??1????????????100000?11?00011?00?01[34]011102?00[01]??011??0??1100011????0??????????????????00?????????[34]020000?0???????00?0011??????0???01?10100????1000?11?1???????0?0212111020?10???0?0?????[12]?0?1002101010?0000???10221?0?[12]?01011?231211?10??00??0010?????????????????????1?????????1??1?1?????0?1???????0??????????????????0??1?????0?????1?0000?00??0???1?????200???????0?????0?0??????????00?1?0?1???????????????????????????0???????????01??????????0??10?00???0??????????01??1???????????????1???00?????0?????1?????100?????????????1??????????????20????????????0?0??1?20????????0?????????????????????0????????????????????????1?0?2???????0?0????????????????????????0?00???????1??0??0??????????1??0?0??0?0??????????0???0??????????1??????????????????0??1?????????0???????1???1??01?1?1?0???11???0???0???1???????????????00????????????????1????????????????????????0???????????0?000000????00???0??00?????1???0??????????????????????????????0??001?01?????????????????????????????1???02????????????????0????0?01?00???????????0??????????????????????????????????????0??0???????????????????????????????????????????????1?????????????000????1?????????????????????10????????????01??0????????????????111?????????0???00??????010?1??????11???1?????????????????????????1????0??????0??????1??1??????????????0??????000?????0?????0???????0???

Patagopteryx

?????????????????????????????????????????????????????????????0?????????????1?00???0??????????2??1?1???11?????????????????????0????????????????????????????????????????0????????1?????0???0?0?????0????????????0[01]0?1000?[12]?????0?01?0???001??0010?0?0??1???221111111110002?01??000?010?1103011?10?00????1??????0??0?????1??[01]01?1???0?1??????????111?????7?100???????????01???1000?0??0???111100000110?00002????00???10?0010?01021?2011[01]1101??10?200100110??11???00?1?1???1?112????1?????????121000110??0?011?0?10??0?1??010?10???????????1????????0?????????0?????0??0????????????1??0???0??????10???1?????????101?211????0??0???0????????????110????????????0??????????????????2?1?????????1?0???000????????21??0000?0???????????????????0?0???????????0???????????????????1?????????????011?1????????????2?????????????????????0??1??????????????????????1???????????????????????1?????????????????1???????????0?????????????00101?01?????1000????????????????????00????????????????1?????????0???????????????????????????????0??0????????0?000????????????????????1??????0?1?????????0???????1????1?????????????????????????????????????????????????????????????????????????????????????????????????????????????????????????????????????????????1?????????????1??0???????00??????????????????????????????????????????????????????0100?1?????????1????10????????????????????????????????????????????????????1????????0??????0??????????????????????????????????????????1?????????????1?10????????????????0????????????????????????????????0???????????????????????????1?????1??????????????0??????0???????1??

Pelecanimimus

?0?1?00000???2210?0001?11000??1?120000?20000000????000010011101????0???01??1?0?????????0?00??20????????????????????????1[01]?1?????????????????????????0010110110212??00??0?00???01?0?????????0?????0?0?0??0?1?1??10?00?0??0?1?101?0?1????00????????1?????????????0???0??????1??????????1?????0?00?10000101?1?10210??0001001?0211??11?10002?2200000000???????????????????????????????????????????????????????????????????????????????????????????????????????????????????????????????????????????????????????????????????????????00?????1???0???0????0?0???1????????????01?1????????????????0????????0????0?010????????000???????00?10???1???10????????????????????0????0?????0??????1?10???0????????????2????0?1??????????0?????0??????????????????????1????0?0??1????????????0??00????0???????????????????????100????????0??1?0??1???0?0????0???0?????????????01??0????1?????????0??0??????0?????????????000?????????00???????0????00000?????????0??0?????0?0????????????????????10?0?????????????????0?????????????????????0????0?0???????????????????????0??????0??????????10?0????????1011?????????1??????????????????????????0??1???01??????00????????????0????0????1??1??????????????0???0???????????10?????????????????????????????1???????????0????????????0??????0????0????????0?0????0?????????????1???????????????????00??????0??????????????????????0?0?????????????0??????0??????????1?0??0???????????????????0?00?????00??????001??????00?????0???????????????????????????????0???????0?00210??????????????????????????????0?????????0????????1??????0010???0????1?0??00???0?????????0???????????

Pengornis

1010?[01]010000??200?0???0??111??20101000?0?0[01]000?????0?101?0?12???1??0??????????????????????0?1???1???????????????????????????????????????????????????0?10?1?010212???0??0?????????????????????????0????????????????1????????????????????0??????0?0??????0??201?111??1?0?2??1?20111020101010011?0?010?1012??????????????1???10?1???[01]?0?????22?????????11[56]????????41??3?????????????11?????????????????????????????????????1?0??20????????????????????????????????????????1?[01]?1???????????????111?1???????111?1?1???1?1??1?001?????????????????????????????1?0???1?2?1???0???1????????0??1??0?????????????????1??0???11???????????0??????????02????0???????????0????0???????1??1?????1??0??????????00????1????????00??2??????????0????????????????????????0?????????????????????????????????????????????????2?0??????????0???????2???????1????????0?????????????????0??0?1?????????1???????????????????????00?????????????????????10??00??????????????????????0???????00??????????????01????????????????0??????????????1???????1?1??0????????0??1??????0?????0?????????????????11???1????0?0?????????????????0????????????????????????1??1?????????1???????0????0????????????0?????????????????????????????????????????????????????????????1???????????0???????1??0???????????????????0????????????????????????????????????????????00??1??1??10??01????1??0????????0?????????????0??????0???????????????????????????????????0??????1??????111??????????01???????????????????????????1????110?1?????????002????1???????????0???????????????0??0???????????????????????000???0?0?1???1?0????0???0?????????????11??

Protarchaeopteryx

0????0??0001?0?10?12????????????10?????????00?????????????????????????????????????????????????????0???0??????????????????????????????????????????????000?110002????00????????????0???????????????0?????????????[01]???0?0?[12]??1??0????1?????0????????????????1???00???????????1?0?00??1????02?????0?0101?012??0110??0?01010012021???11?1[01]1?1?22??1??????01?????????2?1?1??????00?0?????01??01110?0?0010??020????10?11??????11?0??100?????00??0????2??1???????????????????22?1??20???1??[12]????1?00110???1????011?011?????1100?011?11??1?0??0??????????0?0?????0?0?0???0?11?21????????????0??0?????????????????10????????0??000??????001???0??????0?00?0??1?????????????0???????1????2??01?0??000??????0?????0????0?000???0??????????0?????????????00???????1????00????????1???????0????????0????????????????????????00??????0????1??00?????11????0???1?????????????010?0????1?????????1??????????????????????????????0?????0?????????????0?0??00???0?0???1?0?????00??????0????????????0??01????????????????????????????????????????????0??????????????????0?????0?????????????????10????????0100????1???????????0?????????????????????0???????????????0??????1?????0????0??????????0??????????????????????????????????????????????????????????????????????0???????????????????????????????????0????0??0?12?????1??????????????????????0??????0????????????????????????0?????????????????????????????????0??1??????????????????????0?????00???0??00????????0?????1????????????????????????????1??????????0?00???????????????????????????????????????????????????????0?????10??00???0????????????????????????????????

Protopteryx

101??1??0010?0??0????1???11?????1??????0???00?????????????0????????0?????????????????????00?[01]2??0?0?010?????????????????????????????????????????????00?0?102?0?????00??????????????????????????????????????????????00???????101????????????0??0?0????1?0??2011111??1?1?2?01?10011011?0101???0?0?0101?012?11130????210100101211??11?12????22001111200115?????1??412?30????????????11?????11??????000??????????2?????0???11?0??20????02?????01?0???1011?????1???0????????0?[01]?[12]????????????1?111[01]0????????111?111??21?1101?011?1111??01??????????????0?????1?0???1?0?10?0?????????????1??10??????0???1????0?0?1??0??211??????????00???????????2000?0????????????????0???????1??102??01?0??0?0??????0?????0??????000???2????????00???????????????0???????1??????????????????????0????????0????????????????????????00??????0????1?0?0?????11??????????????????????10??0?????????????????????????????????????????????0????0?????????0101?00?00?????1?????????????0???????00???????????0??01???????????????????????????????1???????0????0?1??????0??00?????0?????0?????????????????11???1????010?????1???????????0?????????????????????02????????1??1?11???1???0????0????0?????????????????????????????????????????????????????????????????????????????????0??????????0???????????????????????????????????????????????????????????????????????1?010?0?????????0????????0?????????????????????????????????0?????????????????????????101???00???111???????????1???????????????????????????1?1???1?????????????0????1????????????????????????????0?????????????????????0??1??10???0???1????????????????????????????????

Rahonavis

?????????????????????????????????????????????????????????????????????????????????????????????????????????????????????????????????????????????????????????????????????????????????????????????????0?????????????????????????????011?110?00?0?0?01??11??1?01201?111?1??????0????????????10[01]010??00010???????????????????????????????????????????????????400210??0?12[23]2???10010?0??0?0020101110100200000120101112012?1101211?01010?10002210100110?0010011?0?10??000110102211112?0?1110221211?1010010100?01011?01001201110011010??1?????1???1???????0???????????11?02??????1???0?002?1?0??10????1?0010?00??01?????00?21?????0?1001??????0???1???000?1?21?0?????0???????????0?1????2?????????0??01???0???????1???2?0?000??0?????????????1????????????????0?????????0?????1?????1??????????????11????????0???0?2????????????0???0?1?00??10??10?????????????????00????000????????????????????00?0?0????????10???????????????????????001????????0?0011?0??????????1000????00???????????0???01???????0?0???????0???????????????0?????0?2??0???10????1010?1??00???????00????0???1???0????????????1??????1????1??????0??1???????????????????0??10?0?01?1101?111111001????0?1???????????????????0120??1?????0?????0?1????000000?????????????0???????????????1?0????????????0??????0??0?0??????????????????????????????????????????????????0?????1??????0?????????000????????????????????????????????1???????????111?????1????????0????????1??????????????????1???????????????????????00???????0??1010?????1?????????001?1??0???????0?0???????????????0??????????????????0000?????000??0?1???????????0??0??????0??????00??

Sapeornis

111[02]?[01]11000[01]?0200?110?0??1[01]0????10101??0?0200001[01][01]?0?101?0?21011[01]1?010?0?????1???10????0[01]00?[012]????????1?110110???????????????????????????????????????000111120021????00100?00???1?000??????0?10???0????????????0???10???????010?????????0???00?0??????0?0?1201101???1?0020?1?000100[01]000100011000001010012?11130?0??210110101111??[01]1?10????22001??????015???001??[34]12?3??0???0??????110???0111000000000012011021[12]01[12]??000211?010200201022????01?[12]20?1011?0???00??0????????1?0?1??????????????101[01]001?0??00011?0110?[12][01]01101?011??1101111?00?1??01???0?0?0???0?0??01?0?1[02]?01???????0????1??10?0????000??????010?1??0??21?002?0??0?0[01][01]0???0??????2000?0??1?0???????????0???????1??0?20?11?0??0?0??1???00?????????0?0020??20???0?????0??????????0???0??????21?0??000?01????1???????01?01????0???1????0????1?????2?0??00??????0????11020??1??11????0???0??????????1??101?0??????????????1??[01]??????????????????????11???0??????[01]???????01000000??00???[01]?0???0???????00??????00???????????00?01????0??0????????0?0????????????1?????000????0????????0?000?????0?????0??1???0????????????01?0?????100????1????1??????0??[01]0???????0?0?1?????00?01100??1?111111??111??????0??1?0???????0?010???????2?00???0??????????????????????????????????????????1???????????0???????1??0?????0?????0???????0??0?0????1???1??????????????????????????????0???10?01?0?0000?0?0?0?0??????0?00???0????????00???0?0????????????0?????????1??????????????1??1???00???1[01]000?????1??00????0??????????0???00??????10?01010?????1?????002?0?11???0???????0?0?????????????0??0????????0??????0??0?01??1[01]?0?0???1???0???????0??0??????0???????1??

Saurornithoides

?00110010001?0??0???010?1001101?110100?100000001?100??010??01??1???0????????1??????????????????????????????????00???1??1??11??????1110???1?0??10????000??0001?111?1?0??010???????1?0??????????????????????????????1????????????????????10????0????????????????????????????????????????????????????????????????????????????????????????????????????????4?1?0????????????1?0?????????0?????????????????????????????????00????10???0??1?00?1000?22001?1?????2??????????????????????????????????1?????2???0011?2?????0?1??011?1?????????1????0?0???????0??100????0?02??1???11??????????01???10?1?????????0???0???????21??00?????????0???????1?0???0?1?2??????????????????????1?0???0?????0???????????0????2???????????0?????02????0?????1????????0????????????0?0??1?????????????0?10????0????????0??????0???2??????11????0??????0?0???????????0???00?0????????0???0??????1????????????1??????0?????0??????0001???????0??????????????????????????0???1????????????????????????????????????0????????0??1??0?????0???0???????????0??????0????????????????????????0??????????1?????????????????00???????????????????1??????0?0????????????1??0?????????????????????0?????????????????1???????1??0???????1??????1?????????????????????0?????????1?????????????0??0??????????????????????????????0???????????????????????????????????????00??0?????????????????????00??0??????0????????00????????????????0????011?????????1??????????????????????????????????0??????????????????????????????????1?000????????002????????????????1?1?????????????0?????????????0???????0????000???001?0?1???0??????????????????????????

Schizooura

1112?21101?0????0?????0??101??1?1?0100?00100000?10?1?101?0?21010?1?0??????0??0????0??????????20???0?????????????????????????????????????????????????0?10??12??????????00?0???????10??????????????????????????????????????????00?????????????????1?????????201??????1010?0?1?00?1??20?0??1??????001010?12?01130????210110101101??11?1?????22000101?1011[67]????01??4???3?????????????11?????1??????0??0????0??????????????0???????????1120????0??????????????????????????????012???????????????21101??0??0?011?0?0???111??0?011?????1????0?1???1????0?0?0???1?0???1?0?11?11????????????0??1??0????????0??????0?2???????10???0?????21???????????3100?0????????????????0???????1??1?21?21????0?0???????0?????????2?0?0???2????0???100??????????????????????1?0????????????0???????????2????0????????????????????????00??????0????0?0?0?????11??????????????????????1???0??1???????????1?????????1???????????????1?1?????????1???1????100??1000?????1?????0???????0???????0????????????0???1???????0??????????????????0???????????0?????1????????????0???????????0?????????????????11???1????0?0?????????????????0??1?????????????????????1??1?????????1????????????0????0?????????????????????????????????????????????????1???????????????????????????????0???????????????????????????????????0????1??????????????????????????????????1?????????1??00????????0????????0?????????????00?????0????????????0???????????0?????????????11???0?????110??????????01?????????????????????????????1??01?????????????02???11???????????????????????????0???????????????????????????11???0???1????????????????????????????????

Segnosaurus

????????????????????????????????????????????????????????????????????????????????????????????????????????????????????????????????????????????????????000101100?010?11000?1100?1?100011???000?1?00?0?1?????????????????????????????????????????????????????????0000010000?0?1?00001020?010??1?0??????????????????????????????????????????????0?1????????4???1??1?????????????????????????0111001002000?0011110122000?0?1010?21??0?21101010110?0220010?110???????0??0?0022110?010??1?013?2?1??0000?0?0?0?0000?01?0?20?0??????10????????0???10??????01????????0??0?00?????01???0??1????????????0???000?0???????2??000200??0?00000??0?2??1???????0?0???20???????0???1110??????0????2??1??????????????0?????2????????0?00??0?0??????0???????????????????0?1??0?????????????????????????????0???110???????????101??1???????0?0???????00??0??0?????0?0?1?????????????????1?????????????????????1???0???????????????????????0??0??????????0??????0?00?010???0??????1??????????????????????????????????0??????????????????????1????????????0????????1?000?0????????????0????????1???????????????0???????1??????????????1?????????????????????????0??0100000000???01???????0?????????????????????0??????????????????????000201?????????????????????1???????????????????0????0??????????????????????0??????????????????????????????????????????????????????????????????????????????????????0?????0????????????????????000??????????????1??0????????????????012????????1???????????????????????0????0???????0?????0???????????????????0?????????????1??????????????????????????0??1??????1??0?????????????????????????????

Shenzhouraptor

1102?11101??????0???0???111?????10??0??[01]01000??????0????20021???1??0???0????????0?????0?????????0?0?0?0?????????????????????????????????????????????000111022220????0?020????????????0?????0?????0????????????????10????????10???0[12]1???0???0??0???0??0??0?201?111111100?00101001101??10000??000001011012?111[23]??0?02101[01]0111111??10?12101?22001011100014????001?312[34]30??1??1??0??00002?101[01]100002[01]0000[01]20[12]1??1[12]?12???00011?[01]10[12]0?[12]01[01]221???010????1011?????0???0??????2?1?11[12]?01?1?013?2?1?[01][01]10000?[01]??00011?010011011?0[01]?[01]01?11101?01?0??1??1????0?0?????0?0?1??00?10?00???????0????1??10?0????00[01]?10???010?1??0????10?2???00?010????0??00??0000?0?2100???0??0????0?????0?1?0012??01?0??000??0???0???1?00???0100000?200??0?????0????1??????????????1??1?0???10?0?????????????00???????0???11??????????????2????00??????0????11020??1??11??????????????????00?110100??2?????????0????1?????0??????????1???00?????0??????1???????01?0100000000??1?0???0?0????1000??0??00???????????0??01???????0????????00????0????????1?0????0??0??0??0????????00?1???0?????0??0???0?????0????11???1?0??010?????1????1??????0??1???0??????????????00?0?010??1?11?111??11??0???00??1????????????10???????2???????????????0?1???????????1???????????0???????????????????0???????1??0?0?????????0???????0??0?0????1?????????????????????????????????01???1??00?10?0???00?00?0????????0????0?????????????0?0??1?????????0??11????????????????110?00?1???00???11000???????000??????????0?????0??????????1????01??????1???0??0????11???0?????????0?????????????0??????????????????0?00??1??10000??0?1???0??????????0??????0???????0??

Shenzhousaurus

100010000100????0???010?100010111?0000?20010000101?0??01000010?????0???01?????000??2??0000?????0????????????????????????????????????????????1?10????00101102??2????00?0??001?1??000?????0???10?????????????????????????????????????????0??010?0?00??0????1?????????????????????????????????????????0???????????0??????00??021???10?[01]0002?22000????????3???0000??01[12]00???0?0??0010?0010?01110101011000?201???01111?1110211?1?000?10101??10?100???010011???00??1??????????????????1???????????????????????????????????????????????????010?1???????0??00??0????00?1?????010????0????????????0??????1????0??10?????1?10??00???00000?11??0??????0???0?101???????00???0??????????0??????1?10??00????????????00?????????00??0??00????0?????????????00??????0?????0?0??1????????????00?00????0???11???????????0??2????00???????0???10?????1????????????0??0????????0???0????????????????0?00??????0?????????0?0?00???????0??000???????0????0?0??0??0???0???0?0??????000?0????????????????????????????00??????0???????????????????????0????0?????1?????????????????????????????1???0?0???????????0?????1????1???????????????????????????0???1?0??????????????????????????0?0??????????????????????0?????????????????????001??????????????0???????1???????????02???????????0???????0?????????0??0?0???????????????????????????????????????1???????????????????????????????0??????????????0?????0???????????????0????????????????????????????00??????00????????0??????????????????????0??????????????00???1??0??02??????????????0????????????????????????????????????0?00?????1100?0??????????0???????0??????0??????????

Shuvuuia

10011001??001???0?0??10011?110??120000?10000000101?0000000001001?00010000000?00100010000000?110000101101001?100010001011[01]?01?11020011?12??10???1???1001011101020??0?000??000???1?1001???000?0??0?2????????[01]?01?[01]01100002?01010001110?0?0100?01000001?0???[01]?110000?01000000100000112000014011102?002???????0???0?????????0??????????2??????????100000??5020000????1220?00?0?1100?0??0100?111010000?0?000020020[01]111??001010?01020?211100000?0?02200100110001?0?0001101022212?2001111?13121?010?2000120110011001000?????????????11???????111??110210??0000?100?0???000?0???1?100001111??100100?1100?1?0000010?1?00??21?110?0?0000?1??0?00?0?11[01]0?21???1?0?0?0?000????1?0???0??0??21?????0??0??00?1?0????020??0??0?0000100??????100?1??1?????0?000??????0??00???0??1????1?????1??0?01??0?0??0?1???00??0?010?01?0?1??10?000?0?????021101?000????0???01?10?????00?1?0??0???00?????????1??10????202??1?0?0?????000??0000??0??0??????00001????0001?00??0??0??00??001?00?0?000??????????????12????????????000?0?0??0???????????????00??0?100?????0?1??0??1????10?1??0?0???0????0???0????0???001010?????1????1?????????????0????0?00?????????1?010000100000???000???0?????0??????1??1??????????????00??0??10????0???0?????????????????????0???????1????????????2????00?????0????0??0?????????0??0??????????????????????????????????????????00?0?????????????????????????0?00????????????00?010?0??????????1?0??0??????1??????????0?011110????11?????000010?1100???00????????????0?0000??????0???01000?????0????02111?0??????0??????0???????0??0??1???????????00??0??11?1?00?010??00?0?00?0??0111?0???0??????000????11??

Sinocalliopteryx

1000?0010001?00???2000011[12]00??101001000100?0000100?01?1101?011??0??0??????0??0??0????200000?1???????????????????????????[01]????0??????????????????0?????10?0??01100?0????0??????11?0?1???1?00?1?????00?0??????????0?000??????000????1????0???10???0???00?0?02110?001?000?20?1?0??0??[12]??0??3?1???0000010011?111101???110100120210??12?20101?22001?????01??????????00[01]110?????0??000000?10?01110?0?0111??02010??10?11?????012?[01]???0?1011100??0110????????????????????????????0?1??????????????00110???0?????11?0??0?[12]?11??0???1?1100010000??1???1??00?0?0??0????10?10111?010??????0???????0?00?????00??0????10???00???????????????0?0?0?0?????000?0?0?110????????0??????01??01????2??01?10?000???????????????????0?????0?0??00????0????1?????????0???????1????0000?1????1???????01?0[12]??0?01??11???1??1??????0????100???0??0????10??0??0??00????????0???????????0?010?0????00????????1?00???0??0???1?0?????0?000????????0???????01??0000000?00????0?0??01?00????000??0??0?????????????0?01?0?0???00????00?0?0???0???????00?0???0??????0?0????0???????????00????0??0????????1?????0????0?????10?????1????1?0????0??1???0????0?0??????00??1???0????????0????????????0????0???????1???0???????1??0??????????????0?0?????????????????????00??????????????????0???????0??0???????????0???????0??0?0????0??????????????????????????????????0??????0??0????????????0??????0?0???01????????00???0?0????????????0??0????????????00????0?0???????00???0??00???????00????????????????0?????0???????????1??0???10??0?002???0??????????????00????????????0??????????????????0?00?????0000000??0?????0????????0?????????????????

Sinornithoides

00?????100???02???2011?????11???11????0??0??0?0????01???0?001??????0???0??????????????0???????????0????1??100????????????????0?????1????????????????00?0?00010111?100??000???????1???????????????0?????????????10??0?0??1??0????1?11????????????01??10?0??[12]1?0010?1100?2001000??0000000?2?10??0?0011?012?1?120????0101001202[01]0??10?100?2?22012????????[34]?????0??21232???10?1??1??0?002?1?10??0???????????1??????1??100?01[01]?0??100101020?010011???01011????1???00??1?0???1?012?0??1??231?????0110?012010001102100?201111011111????????10?1??????2???00??????0?00?02?11?0?1??????01???1??0??0?1??00???0??????0??????2?00?0?0?????00?200???????0000?1???0????0?????000???0???1????2??01?0??0?0??????00?1??2????0?010??00???????????0???1??????????????0??1?0??0?0??1????1???????0????????0???????????????????2???100?0????00???1??00????0110???????1?????????00??000?0???????????????1?0???????0??????0?1????0?????????000???????0??00?000???0?0?0???????0?????000????10????????????0??02?????????????1??00?????????????1??10????????1?0????????????1???0?????00?????0????10????0????0?????1????0??????1??????0?????????????????????02????00??0?000000010???10???0???0???????????0????????????1?????????????1?0?????0???????????????0???????1???????????0???????????10?????00??0?0?????0??0?0????1??????1???????????????????????????0??????0??0????????????0????????0?????????????????????????????????0?00??????1????????0???0?0??????00??????00????????0????????????????????????????00????1??????????0????????????????????0?0?????????????0??????????????????0?00?11?????0??01?0?1???????????????????0??????????

Sinornithosaurus

1010?00100?0?02?0?201101101???101000001200?0000100?0?10100001??????0??002000?011010?0??0000?1??0000??10??011??0???????????0?1?????????????1?1?1?????0012000000[12]01?0001100001???1[01]11?????00011??1??????????????????1?????????????????????????????????????0?20110111111002001?1001?00??0??2?????00?1[01]1?0?2??11201?0?010100111210??10?12110?22011001000013??0?00????????????????2?????????010100???1???0120[01]1?2?0?1211100210?01010?1?1021101001???????11????????????????????????0??1???312?10001?00111?110011?21001001100112000?1??????100?10?01???0?0100001?0??1?01?11?001??????0???11??0??001?1??1??0??0??0??????0??000000??10?0?02??10?01?00000?1?21?????0??0???00?00???01?0??10?01?00?0?0??????00????1??????110?002?00?00??000????11???????000??????1????000??1????1???????01?00??0?0???11???0??????0???2?0??00?0????01???1?0?0??1?1111???????0????2??????1?10?0?????00????????1??0??????00??1?0?????0?000??100???1000????????100?00000??0??1?0?100??????10????????0???????????00?01????0???0??100??000???0???0????1???????0??1?0??1???0?????????????????0??0???0????1?????1?1??1?0??0100???01??????????00?01????????0?????????00?1??00?001001000010021??0??00?0?0???????0??1????0??01??0???0?00???????1?0????00?????0?????????????????1???????????0????0??0????0??????0??0?0????????0?00???11?0?12?????11????0????????????????00?????0??00???????????0??????0?0?0?00????????000000?0????????0???0???????0???????10?0???0000?0???00???10?11?????2?000????0??????????0?1???00????000??010001??1???0?112????1??????0??????00?0??????????0????????????10?0??000?101?1000?10?0?1???0?0????????0??????0??????????

Sinosauropteryx

100000010001?[01]200?00?10?1000??1?1001001101?000?0?01010010?0[01]0?1????01100??00???????1?000?00?120?1???00000???0???????????????????????????????????????00?0?0000010??000?00?0???????????????????????0?0?0??????0??10?00000?0?1000100?10??0[01]0?010?0?01??10?000211?010?1000??0?100000002?00004?1??02001[12]?0011?111210??0111101010210?100?10101?220010????????????????00110????0?0?000?100010001110000001010020[01]0??[01]1?11???0?012?0?0?001011100?001002[12]0?10021[01]???0??1010??102?1?001?0??1?01312?1000110?010?000011?011002011100?011011000?0001??1????0?00?100???0?0?10?10?11?100??????0??1?0??0??000?1?00??0??001001??0???00100?0?00?00001??0???0?00000?012?0????0???01000??01??01?0??2??01?00?100??????00????1????0?0?0?001?00?00????0????1????????00??????00?0??0001?1????1???????01?01????0???11??????1???0????0??100?1???000???10000001??000???0???0??00??????0010?000?0??00??????0?1?01???0??00????0???0??0000????0???0001??????001?00000??00??001??1?0?0???0?000??0??00???????????00?01?????0?00??0000?000???????????00?0???1000???0?0????0?0?00??0???0?????00?0???1???110??0?0????0?0??01001???1????1?0????0???0??0????0???0?????0??1???0000000000?0?0??01000?0????0????0?0?0??????????1??0???0??0???????0?0????00?????0?????????0???????1???????????0???????0??000???0?000?000?????0??0?0????0????0?????000??????????????????1?0000?0??00?00???0???????0????0?0?000?00?????????0???000?1??????????0??0????000?????00?0??000?0?0???00???0??00????01?00?????0?????0?1??0?00??0?????00????1?000?010010?00210?00?1?0?????????00?????????00?0?????0?????0??????0?00?0?00000000???0?????00???????0?????????????????

Sinovenator

000010010001??20010?110?1100101??101000[12]000000???0?011????????????00???0??0??011010??????????2??1?0???0????????11?0?1?11[12]?00100010001??2????????????0010?000102?2?000??0?10????1?1???0??10???????0???????????1?10?1000??1???10??0?1???000??1?00???11????012111?1111100?2?01?10???????00????????0??[01]???????0???1?????0???1?????????????0????0??????????3010000??21[12]?????11???0???00?0??101010000000??002021?211?1111100011?010100201?211?1000????0101110?110?000?0?01???2?0?2?????1?2?1?????0?1000110101011021001[12]????0?11???????????1???1?????2?0??0?1001????1?02?????01??1???02???1?10?10??1?00????????10???00??200?0[02]?0?0000??0??0???0??0?0?0?1?21???????0???????????????0??2?????????????????0?????2?1?0?1?????0000??00????00????1???????00????0?0?????00??0?????????????01?01????0???11??????????1??02???10??1??????????10?01?1?0??0???????0???????????0????0?????10????0???11?0??0???0????????????0001????00??000???0???0010???????00001??00??0??0????????????0??????????0??0??2????????01???0????????????0????1??????00????1??????1????????????0?????0??????????1???????01?0??????00??0?1????1??1??????????????????????????0?1?1?0?00100000001002?10????0?000????1??????0100000000?0????0?0???????????0??0?0????0?????????????????1???????????????????0??010?????????0??????????0?0???????????????????????????????????????0??????0??0?????????????????????????00????????00????????????????1????0????0?1?????1??0?1?00???0?????1??0????011??2????????0????????????1???0?????0?????10011????0???002???11???????????000?????????????????????????????0???????0??0000??0?0???10??0??????0????????1??????????

Sinraptor_dongi

10000001000110010000110010000000100000120020001000001011211100100111000020000000011110100[01]1010000101010010010110001011112?0?0101100001010101101010010000000000000?0000100100??1110010001000000011210110011211002101100020111001001110001000010010010?0000310??0000000000000?00?????????????????0???????1???????10????00??????0110111????122001100000??300000?0????????10?1000??????0?0?01[12]1112201100002[01]010100011?10110122[01]000111010101001[12]001201100212000001101100102212000112011001122100011000100000010100100201100000110????????0?0000?0012000?1100000???0?101?11?0001000?10111010??0000110000?0000?000?00000100?100000000??01010000?00?00000?10?1?01??100??0?2000000101??200001?0????11?0?00000?11?10011????00?000000110?00100111100???0001??000?0???001001211110101200?1001101?010?110?000010?000000?0?0??0?101000?0??01?000010?0011?110?010012?010??0????0?00?00010100101110000000100110000???00?00000000010000001000100?00??0000001010000010??10??0?????0??0??????????0??00?0000000?100?100000000000????000?0?0??1?10???100?111000?0???00?0??10?00?0?0?0??000?1????0??00??000??100110010???11?000000010001?00?00000?01??00?00??000000000????????10000???000000000001??00000???1??0?00000000000100??00??100?0000??01?????00100000?00?10010010?1?100??0?00?00??0??00000???0010?00?00?0???????????????0???00000?1001?0?010?000000?00?00??????????00???00000?00?00?0?0??0010000000?11000??01?0?[01]101100000????00100?0?0001?0???000???????010100000?00000?00010?000??00??00?0?000??0000100000101?00011?00001?00001?01??00001100?01000?10?11000??0?0?0100?0?0000?0000??000?0000??0?0?00???00?1100?000???0010

Sinusonasus

0000?00100?1????0?2?110?1100??10110000?2010000?1?0?0??0100002??1???0????????????????????????????????????????????????????????????????????????????????0012?000102?1?000?0??????????1????????????????????????????????????????????????????????????????????????????????????????????????????????????????????????????????????????????????????????????????????3??0?????21[12]320???1?1??0??000?20?????????????????????????????????1???????????0200??001?220?10??????1?????????????1?012????1??????????0110??1[12]?10??11?2?00?201111011?1?????????1??????????????00???0???00?0???1??010????????1?1?????000?1???????????0???????21??0??????????0???????1?0?000?1?2?0????????????0???????1?0??20?0???0??0??????????????0??????1?????????00????0?????????????00????????????000??1????1????????1?01??0?0????????0??????1???????????1???????????0?1???????????0???0???????????0???0????????????????1??0??????0?????0??11?0?00?????????000???????????????????????0?????0?0??????00?????0?????????????0??2?????????????1???0????0???????????????????????????????????????????????0?0????????10?????????????????0????????????????0????????????????????????1?00???????????????????????????0??????????????????????01?????????????????????????????????????????????????????????????????0??????????????0??????????0?0???????????????????????????????????????0?????????????????????????????????????????????00?????????????????????????????????1????????????????????????????????????????0????????????????0???????????1??01????????002????????????????000???????????????????????????0??????00?01?000?000?0?0??0??0??????????????????????????

Syntarsus_kayentakatae

1010?00100010020010010011011??00010100120010000000?0????01100?01?10???1???0100000001?0?0?000?1?0000????010010????????0?110????0?????????????????????0?00000000000??0?0100000?000100????1000000?0?0???0000???0?1???0???1??????2[12]??????1?????????0??????????1?0?????????????000?????????????????????????0??????????????????????0??????????002?????????01??????????????????????0??????????1?????0?3???????????????????0????0?0??010??????????10?10?011020?0?00??0?2?????21?200??1??0?1001211?111??????1???????????????1?????????????????????0?0000??1??0???0????????0???0?0??????0???1??????00????????0011??0???00??000??00?0???1??0??0??0???0???0??????0??1???????????0?1?0??0???0?00??0????10????????????0???????0?????000100??010?1?1?0?????000???????????111?11????100011??00?00000111?0111111?00??1??????0??????1?000??00??1?00???0?0?0??00??0??001??????000????01??00?000?0011??1???0?002??0?00????1000?1000?????000????01??0????0???10?0?00??00????1??00???????00?????????0??0???100????1?0??????0????00??????????1???01????100?1??10???????0????1???????0???0????1???????????00???112??0??????1????????????????0?0??0?????????0????????????????????1????0???00???????1??0???????????0??00?????????0??0?????????00???00?????0?????00?00?0100101?0?100???00???00??0??0??0???0??????0?0??????????????????????0000000??00?1010?0???????????????????????????0?00000?00????????00?0?000??0??0??0??10????0000?1?????0??00??000??????000?1?0?????0????00??0?00??0??0???1????????????????0????00????10?00?21?????0?0?????????0??????????0???0??0??0????????0???????????00???00????0???0????0???0??????????01?????

Tanycolagreus

??0?????0002?0?1??20??????0????????????????0?00????0??001?1[01]10?1???????????????????????0?01????100??010??1010????????????????????????????????????????????0??????????????0?????21???????1?????????0?????0??????0????????????????0?????0?000?0000?0?1?????00211000001000020?100000001000003010?000001??0110?011010??011100120210101212?10?222001????????????0???????????????0?1?????001?????????????????????????????????0?[23]11???01????????????0120110??100000?01010001?221[12]001?11011?131211??011000?0000001110110?2?1110000110?????????0???01?0?2?010?0?????1?????0?11?1?????0?0100??0??0???0?1?0000?000?0?0?1??0??1000?0?0?????00?2?1????0??0000?0???10???1?0???000100??0?1????2?000?10?10010????00??????1???1000???00?0????????0???11???????00???????1000???0001???11?00110?01?0???0??101????01?1?00???0?00???10??????00?0000?00???0?11?0???0??0??0?[12]?????0?101?00?0????1???????11??001000?0??0??????????0???000???????????01?0100?000???0?0001??????0???010??????000??????????000?01??0??????0?0???000????0????????1?0???0?0?0??0??111???00000?00000?????0??0??110??1?????0???0?????001????0??????1?011??0??10?????????????????00??0?00?0??????0?????0?10?0?0???0????????00?????????????0?00??00??????0??000?????100???0?1??????????0????0??000001?0?????????????0?????00?0?01??????????????0?1??0?????????????????????????0??00???????0?00???????????0???????00???0001???????????????1??0??????00??0??????0????????0????????????00??????00??????0?0?0??????????0??????000???????0??00?0?????10010?0???00???1?0?????????0?????????????0?00??????0?0??????0????000?????0??0?0???0??0???????00????0???????????

Tawa

1010?00000?0?02?0?0000001?1???20001100?110?0000?0??00?0001120110?1?0?00?2??0??0?????1110000?100????????021000????????0100????1???????0??????????????0?00?00001000?0??0?0??00???1????????0?0?0????00???????????11??00???0????01?0??10???[01]?????0????1???????211??00?????????????00??1??0??3?00??????00?002?0??0011??000?0?0?021?0??01000?1202001????????0???0??0??0?????????0??0??0???1??000?01?2????0??2001?00??11?100021[12]?010???00??????????0?1?010?2????0????001??0?1?1?0?1??01??000?21?00001????0????011?01?0?10?0??????1??????????0?????0000?0???0???0?00????0?0??20???????01??1???0?00???????????????0?0?0?0?00??0??0?????000?00???0??000?0?????1???????0????????0??00????20??0?00?00011????00??0?1????01??0????????00????0????011????????0????????0??000?11??1?1??111??00?11????0?????00010?0??0??00??0??00??0??????001?0?0???0?1????????00???1???????0?0??0?????0??????????1?0??????0???0???????00?00????0???0000????????1????0???0??????????0???0??0????????0?????????????0??0???0????0?????????????????0??????????0??????0??0???0?0????0??????00?????????????1??????????????????021?001????1?????????0????????0?0???????0??1????10??????0????????????0?1??0???????1???????????0??0???0????????0??????????????????????????????????0????????0?011?????0??0?00000?????????1??????0????????????????????????0????????????????0??????0???????????????0?????????????0????????00?????0010101?????????00?????0?????0??0???001?????????0?????????????0??????0????????????????????????????0??00??0??0??0021?????????????0???0????????????????????????????????0????????00???0????0?????????????0?????????????????

Tianyuraptor

??0?????00????0???0?11?0??????1??0?00?1?0??00?0????????????????????????0???????0???????0?00?????????????????????????????????????????????????????????0????00000000?0???????????????????????????????????????????????10?????????0?????1???0??000????????0??00201??11??100?2??1?00?0000??1??3?????0?0010??12???120????0101[01]011021???11?121?1?221120??0??01??1??????[23]12[23]?????0?1??2????0?2???11?????00000?12010?211?12?11??0111????????00200?10010???01?11????1?????????????????2???????????????0?10???1???1?11??????0?????????1?????????00??????1??????1??????0??1?00?1??2?1??????0???????0????1????1?1?????1??0??????1????????00?00????1??????0??0???2110??????????0????????????????11????0?0??????0??????????????????0?????0??0????????????????????????1?0?????????????????????????????????????????????0???????01?????????????1??0??0??11???????????????????11?0??00????????????????????????0?????????????00?????????1???????????1???0000????????0???????????00??????0???????????1???01???????00???????????????????????????????????0????????????????????????0???????????1?????1????0?????1??????0????1???????????????????????????????10????0??????0??????00????0????????????????????????2???????????????0?1?0?????????1???????????????????????????????0???????0??????????????????????0??0?????????????????????????????????????????0????????????????????????????????????0????????????????????????????????????????????01????????0??????????00?01????????0??????????????????????????????????1??0???????????????1???????????????????????????????????????????????0?00????000?010?1???????????????????????1??????????

Troodon

??????????????01????01?0???1????????0???0??0????????1?010?1010?????0??0010011011011200?0100?2????????????????000000?101101110000??110012????????????000010001?[01]1[01]?100?0010???????110???1??????????????????????????1????????????????????????????????????0??????????????????????????????????????0??0???01??????010??????????????????????????????????????4?????????????????1?????????????1???????????????????????????????0??????????????????????????????????2???????????????????0?11??2[23]?211?001?1?0?[12]??11011?21??020111101111???????????1??0??1?2??1?0011?????????2??1??0??????0?0???0?1??1??1?1?0?????????????0??????0?000???????0?????101???000?0???1??0????0?0??0??0???0110??2??0???0???????0??0002??2???1??01??????????2????0?1??1????0??????0011????????0???11?????????????????0??0????????0?10???0??0????????????00?????????1???0?1???00???0???0????1??????0?0?????0??00????1????????0????1???????????1?0??????001?0???01????????????????01?01?0?????010?????????1[01]111121??1???01???00????????1??????0000000??????????1??????1??0???1???????????000??????0??????????????????1?00???????????????????0?00??1????0??0??00???????2?1??0??????????????????????01???0???????0??????????????????????1?00??0??00?????????0?????????????????00????????????????0??0?0??0??0??????0?0?????????000???1?????????????1??01???????????????????????????????????????0??????01???????1?0???00000000?????????000??????????01????1???0?100??????????0???????000???1??????00????????????????0??0???0????10000????1???0021???10??????????1010????01???0?????????0???????00???0??010?000??0??1?0?10??0????0?0?????????10????????

Tugulusaurus

????????????????????????????????????????????????????????????????????????????????????????????????????????????????????????????????????????????????????????????????????????????????????????????????????????????????????????????????????????????????????????????????????????????????????????????????????????1??1?11000?10???0???????????????????0?????????????????1?0?????????0??1??????????????????????????????????????????????????????????????0110?1?02111000?010?1?01?2212??1101?11?011211?????????????0???????0????????????0?????????0????0??????1??????????????????????????????0??????????????0?1?00?????????0??10?1????????????1?????????????????????????0????0???????????????????0???0?1???????0?????1???10??????0??????????????1??????????????????????????0?????1?0?1???0?????????????????????????????0???0????????0?00????????0????0?0???????????????????????????????????????????0???????????0??0??????????????????????????????????????0?1??????0???1?????????0????????????????????????????????0?????????????????????????????????1???????????1??????????0???1?????0?????????????????????????????0?1????????????????????????0???????????????????????10????????????????????????????????0?1?????????????0????????????????????????????????????0??1?0????????????????????????01?????????????????????????????????????????????????????????????????????????????????????????0??????????????1????????????????????0?????????????????????00????????????????0???????????????????0?????????????0??????????????????????1????????????????????????????????????????????0????????????????????????0???????0?????0???????????

Tyrannosaurus

10010001000210020120110011001010100000121000010100001101111011110111101120000111021101011010020110000100210101000000001110000210100012110100111010010000000000000000000000010121000101010001100211100101112010?2001100020010001101[12]1100100000001001000000221100000100001001000100000010040100010002?01?101011010000[01]01000?0??0??12?2?????220010????00131011000010121101001000101000010001110111211210120000201111011111131210101101111010000012001002121000111010011022110001010110231211000110001201100111001001011000001100???????00101000002111000110001000?10101101010010011111010000100110000?000001100000012000021000000000100010100040000012110?010?00010001000002100??2000100010001000000000?11010011000011100000011??0010011?1000?10001000100010?0000012?111000110001001?01001001100010010001100000?00010100000100111010000000000010000101120111000???00000?100?000020111001011100011000000000?010001000000100000?11000000000?000?000100110?0011010000?00000????????000?000000000000000010000000000?00?000010000111000010001110000000000000000100000001?00001100000??000000000100110010110100110000010001000?00000??100000100000000000000000000100000?1001??2?00001?000000???1??0000111110???101011200001??0?1?0??1112101??????001000?0??1002??00000000?00??010000000100000?00?000010?????????????0???0010011001???0100000000?00?00?????0????0000?00?000101000100000210000001?11000?0010001111100000?00000000?000000?0???0000000?000101010000111000100?0?00000000010110000??0010000111101?0002100000100001?100?00000??01?0?0000?00100001?0110010?00000000000000000001?0000?00000100001010011????0010

Unenlagia_comahuensis

???????????????????????????????????????????????????????????????????????????????????????????????????????????????????????????????????????????????????????????????????????????????????????????????????????????????????????????????0??1??0?10??00101??1???1??32?1??11????????1????0010[01]0?0??2???0??0??????????????????????????????????????????????????????4?1??????????????1???????????????011101002000?0120111212?02?1100212101000?100122??1?0110??010?1100?10??00????????1???1????????????????????????????????????????????????????????0???????????1?????????1??1?1???????0????????????????????1???0???????1??1???0?20???????1001?0????1?????????????21?0?????????1???????????????????????????1?1?????????????????0?00??0??????????????????????????????0??00????0???????????1???????????????11????????0?????2??????????????????1?0???0??1?1???????????????????????00?????????????????????????????????????????????????????????????01????????01?0???0??????????????????????????????1?????????????000????????????????????????????????????????????0???????????????????0??????1???????????????0???????0????1????????????????????????????????0??0??0110?0????????????????1??????????0????????012???????????????0?1????????????????????????0??????????0????????2?????????0?????????0??????????????????????????????????????????????????????????0????????????????0???????????????????????????????????????????????11???????????????????????0??????????????????1?????????????????????????????????????????????1???????????????????????0???????????????0??1???????????????????0??????0??????????????1??????0??????0????????10

Utahraptor

??0?00?100011001???0??????0????????????????????????????????????????????????????????????????????????????????1????????????????????????????????????????????1?????????0?0????????????????????????????????????????????????????????0??1??00??2?????1????11????02?????????1??????1?0??0?????????????????????????????????????????????????????????????2????????????????0????????10????1??0?0????01??00???1???????1???1??1???1???1[12]?1???0?10???????????[12]2001??1????????00???0102211??00????1?23???????????1?0???0????????0???????????[01]???????????0?0??????????????????????????????????????0??????????0?????0?00????0????0??10??0?0?????????2??????????????????10??????????????????????????????0???0??0????????????1?????????????0?0???????????1???????00?????????????????????????????????0?????????????????????????????0??????????????0?????????????????????0?2???????????????????????????1??0????0?????????0000?????????????0?????????0?????????????00????????0???????0?????0?????????????0????1???????????????1???????????????????????????????1??????????????????????0?????????0????????????????????????????????????????????????????????????????????????????????10?????????????????0??0??????011?00?0???????????????????????1????????????????????????????????????????????0??????????????????????????????????????????????????????????????0???????????????????????????????????00???????????????????????????????0????????????????????????????00????????????????????????????????????????????????????????????????1???????????????????????????????????????????????????????0?0?1?????00?????????????????????????????????????

Vegavis

?????????????????????????????????????????????????????????????????????????????????????????????????????????????????????????????????????????????????????????????????????????????????????????????????0????????????????????????????????????????????????????????201??????1????001?2?1???1?11????????????????????????????????????????????????????????????????8??2?????????????????????????????11????????????????????0????????0?????????2????????????????1????2??1?????????????2??????????????????12?????????1??11??????????????????????????????????????????????????????0?????????????????????????????21???1?????????????21???????????????????????????????????????????????????????????2????????????????????????????2???01??????????????????????????????????????1?????????????????????????????????????????????????1???????????????????????????????????????????????1?????????????????????????????????????????????????????????????????????1?0??????????????????????????????????1?????????????????????????????????????????????????????????????????????1?????1????????????????????????????????1????01????????????????????????????????????????????????????????????????????????1??????????????????????????????????????????????????????????????????????????????????????????????????????????????????????????????????????????????????????????????????????1?????????????1?0????????????????????????????????????????????????????1??????????????????????????1?????????????2???????????????????????????1??0?01???????????????????????????????????????????????0??0????????????????????????????????????????????????0?????????????????

Velociraptor

10000001000110?0010011011010101010100011001000010010110100102011110011012000101101010010100?21000000010020020000000001111?00110010101?010??111101101000210000010[01]000000000000111?01100?????1?0???0?0??????2?11120?100??21?10101?1?11?0010?000101111110?012211001111100020?10100010[01]0000020????000011??12?1?12010?0011100120210??11?11112?2211200100001401000010[12]024200010?1002??0000201011101[01]001100012011121001211100011?[01]1010010102000101111200101210?11000000?1?10221?012001111023121101010001100101011001001101101012010?????????00010?0102011010000000?10?021110001?0100002?110?0000000010010100000100100000200001?000010000200100010000000102110?0?0?00012001000?001010?20001?0010001?00010000?010??00?0000002000?00??000010011???00?1000???0?01000?0000012???1??0?11011?00?00?0???11???00?00?00?002?0?01100100000???110000?1?1111???0?0?01?002001?0?01100?0?00?00?00002?111001??0?000??1?000?0?0?001001000001000??11??00100?0000000?000101110?0?00010000?00000????????01100?01?0000???00?1001?0000?00????0??01?0100000?010000011?0?0100001?0?010??001000??000?110?00?1?10?0101?0100??001?0111?0?0000?010?????0?00000????0?2?1000020010010000100211000?01?0?0????0??00?01010010011?000?0000?????0?110000000???1?0?0???0???0???????1??0??????1?02???0??0?00?00000000000???0???0?000000?01?????????????0???0????????????0??0000????00000??????????0000000?0?000?001??????00000000011???0??00000?101?00001?0000020??100000?00??0001?010000111020000000?0??00?0????00100000?0??000?0010000??1?010?1121000001???01?00?0?0?00???1??????0?00???0??????000??00000010000001000?00?0000????0?10000??00000????????

Xiaotingia

1112??11?0?0????0?0?11???00???2?1000??10?11?0?0?1?????01?0??1???1??01?????????????????????????????0??0??????????????????????????????????????????????0?00??0012?1???0??1??0??0??1010??????001?0?0?0?0???????????????????21???10?????????0???1????0???10??00200??101?10??????????1????????2?0????00101??12???1[23]??????101?012?211??10?02?12?201?2??????013?????11?????????1????????????????10??1??0100??120101?1??111?0??2?????????2?????1???0????????????????????0??????????1?????????????????1????????????????????011?1?0011??11???1????????01??????0????1????0?00?11?001??????0[02]???0??0??0??????????????1?????????????2?????0?0?0???0???1?00???00?2?????????0???10???????1??1?2??01????0?1??????00????0???????????????????????0???????????????????1??1??????????????1???????1????????0?0?1???????????0????????11??????0?????10??0?1???11?????????????????????10?????????????????????????????????0????????????????????01??1?????1????0?????0??1???1?0???????00??????0????????????????1???????0????0?????????????????????????00???1?????????????????????????00??????????1?????0??0?1??????0?00??1??????????????1????????0??????????2?0?111?0??1?1?????????0??????????????0??????????????2?????????????????????????????1???????????????????????????????0??????????0????????????????????????0????1??????????????????????????????????0??????????0?00?????????????0???0?0???????????00?????0???????????????1???????????111???????0???????????00??????????????????????????????????0???????????1??1???1?????001???1???????????????????????????????????????????????????1?[01]??10???0???1?100??????????0?????????????????

Yanornis

101??2?10110?[01]200?00?????11?????1[01]10???0???00????????10??002??111??0???0????????0?0??????????20???1?????????????????????????????????????????????????0010?10[01]?0212??0??[12]0?????????????????????????0??????????????????????1????01????????0???00?0????????0?1201?111?110102?01?10111020?0101?1?1100010?1012?111[23]00???210100101101??12?1?????2200112120?117??2?011???????????????????1??????1[01]??0?0?????????????1??????????11??1?20?[12]0??200?????0????????????????????????????2?2??????????????1210?1??0??1?011?0110???11?00?011?11????0??1????????????0?????1?0?1?000?10?00???????0????0??10?0?????0??0????0?0?1??0??21100?????0??10???????????3100?0????????????????0???????1??012??01?0??0?0??1???00????1??????0000?02????????111?????????????00????0??1?0????0???????????????0???2????0????1???????????????????00??????0????1???0?????11????????0?????????1???10??0??1??????????????1??????????????????????1?1???????00????????010?1110100????1?????0???????0?0?????0?????????????0?01???????????????????????????????1???????0????0?1????1???000?1???0?????0?????????????????11???1????010?????1????1??????0?????????????????????00?1????????????1??1?????????0????0??????????1??????????????????????????????????????????????????????????1???????????0??????????0???????????0??????????000???????????????????????????????????????0??????1??1????????????0????????0?????????????0????0?0????????????0?????????1??????????1?0??11??0?00???10???????????02???????????????????????????1?4???1?????????????02???1????????????????????????????0????????????????????????????????0?0?????1?????????????????1???????0??

Yixianornis

1????211?11??02?????????????????1??????????????????????????????????????0???0???001????????????????1???1?????????????????????????????????????????????0??0?101?021??????200????????????????????????0??????????????????????????????1??????0??????0?1????2?00?201?111??10102001?1011??2??0??1?1??10000011012?111300???210112101101??12?1?????220011??20?117??2???????????????????????11????110?????0000?????????11?11??0??010?0??20?2010201??00?0???01?0??0??1?????????1???2?2?2??????????????1210????1?11?011???00???11?00?011?111?1?0????????1???00?0???????0??0000?1??10???????0????0??00??????1????1????1?????0??2111?2????0??10????0??????3110?0????????????????0??????????0121?01?0??0?0??1???0??????????0?000???20?????????1?????????00??00??????01?0????0?2?????????????0????????0????1????????1??????????00??????0????1???0??1??11????????0??????????0?110??0?????????????????1???????????????????????????0?????????????0010011101?0?1000?????0?????0?0?0?????01???????????00?01???????????????????????????????1???????0?1??0??????1?1??00?????0?????0?11???0????1?????11???1????0?0?????1????1??????0??1?????????????????????1??????1?????1?1?11?0?????0????0??????????0??????????0???????????????????????????????????????????????1???????1???0??????????0???????????0???????0??000????1??????????????????????????????????0???10?1?010??0?00?01000????????0?????????????0??????0????????????0?????????1?????????????1?11????00???10???????????02???????????????0???????????1?4???10?1?????????002???11???????????????????????????0????????????????????????1????0??0?0?????1???????0??0??????1??????????

Yutyrannus

1000000100?200220?201100010???20100000121110211?00?01111?10111101111101?2??0??11?11?0011001?110?1?0????021010???????????????????????????????1?1?????000000000000000??100100101?10101??????001??1?0???0??1?2?1??2??0100???????0????1????2???0?0??0????0?00?211?000??0???10?100??00?2??0?03010??100011?001???1101???0110000??21??111?101?1?22001???????????????12?0?1[01]0????00????10??01?0?1?1011?3112??020?00??1?10??1?1011?2?0[01]??00?1??0?0?10?1???1??2?1??0?1?1?1??01?2?1?0?0?????102??????0010001?0???0?11?0???02??1??0?0?10?11?01??000????00?????101??00??????1?011101???????12?1????0??000????0????????1?0?0???10??00?0001????0?0????1??00000?00?10?????0100???0??00??2??0??20?01??1?0?0??????0?????1????????0??1?????00????0?????1???????100????1?1?0??001??1????1???????01?????1?0???1????1?????10?????0?000011??0?00??110?0?????00????????00?10??????00?0???0????0?????????1??0?0????0???0?0???0?0?0100??????????0????????0????0???0???00?0?1?0???????00??????0??????????00???00?0???????0?10???0??1010???0??????????11????10??0?00?????00???????????00?????????1???????????0?????100????????????????0?????????0?0?000???????????0??0??????0???????1??0?0????0????0??00??0??????????0???1?10?????????0???????????0????????????????????0?????0??0????0??0?00?????00?0??0???????0????0??010?????????????????0????????????????00??????????????????????????0?0?001?00????????10?0???0??1?????0??00??0????????????00??????0???????00???????0???1???00??0?00??0?????????????010000??????1?00110????000121?0???????11211?????????????????0???????????0????????0?????00000000??01?0??0???00???0?????????????????

Zanabazar

?00111010001002[01]01??010?1001101?110100?1001101010100?1???0?01?1?1?00??0000011011010100?0100?1?????????????????010?0?10112111?00???1100?2????????????0000100010111?100?0010???????110??????????????????????????????????????????????????????????????????????????????????????????????????????????????????????????????????????????????????????????????????40101?0??????????111?????????0?????????????????????????????????????????????????????????????????????????????????????????0??1??23????010??????2??1????1?1?????????????????????????0??0?0?1?0???00?100????????1??0?0?1?????????1??1???0?1??00???0?0???0???0???????00?????????0??????0??0???0?????1???????0???????????0??0???0??????????????????????2???1?????????????02????0????1?????????0?0??????????0?0????????????????0?10?0??0?????????10???00??0??0????11?0?0???????0?11???0??????0???00??12??????0??????????10??000??????1?????001??1?0?0????0001?????????01???11???????????????????1??1?0??????1???????????????????????????0?0??????00010?1?0?00?1?00??????????10????????????1?????????????0????000????????????????0???00????00??????????????????????????1?0?00?????????1??0???????????????????????????0???????0???1??0???????0???????1?00???????????????????????????????0???1????????????????0??0?0??0??10??????????????????00??0???????????????????????????????????00??????????????????????????0??0?????0?1?0??000000?00?????????0?0?????????001????1???????00????????????????????????0?????????0?????????????????????????1?000????????0021???1???????????????????????????????????????????0??????????000??00?1???10??0??????????????????????????

Zhongjianornis

1012?11?0100????0??????????????????????????000?11??0?????0????????????????0????00?????????????????????????????????????????????????????????????1?????0000??12?????????120???0???????0??????????????????????????????????????????1?????????1???????1?????????201??????1?0????1?10?11??1?0100?11?000010?1??2????3?????2?012?111201??1[01]?1?????22000??????01?????????4???3??????????????1????011?????000?????0????12?11??????1????????[12]?10?20??00?01?????????????????????????0?1?2??????0???????111??01?1?????11???1????0??01?101????????????????1??????0???????1?10?00?10?011???????????1??1???????????11???????2???????00?????????21???????????[23]100?0????????????????0???????1??0?0?001?0??0?0??0???00???????????000???2??????????0???????????????0????????0????????????1???????0????????0????????????????????????00??????0????0???0??1??11??????????????????0???0???0???????????????????????????????????????0????????????0??????????0100????0???0?????????????0??????00???????????????01?????????????????????????????????????????0??0??????1??0?0????????????0???0???????0??????????1?????1?????????????????????1????????0?????????????0???????????1???????0????0??????????????????????????????????????????????????????????????????????????????????????0???????????????????????????????????????????????????????????????????????????1??????1????????????????????????0????????????????????0?????????????????????????????????????????????????10????????????0?????????????????????????????????1??1??????????02???1????????????????????????????0???????????????????????????11???0???1????????????????????????????????

Zhongornis

?????????1??????????????????????1???????????????????????????????1?????????????????????????????????????????????????????????????????????????????????????????12???????????????????????????????????????????????????????????????????????????0?????????????0????201??????1?0????1?10?1??1??0??1?????????0????2????20????0101?012?001??1??1?0?[12]???00[01]??????01[45]????????41??3???1??0??0????1????????????????????????????????????????????????122????0????????1???????????????????1?1?1????????0???????1[01]0???1????011?0?00??111101?011?111????????????1??????0?????????????0??0?1?????????????1????????????????????????????????????0?????0????????????[01]110?0????????????????0???????1???????01?0??0?0??????00???????????00????????????????????1?????????????????1??????????????1?????????????????????????????????????????????????0????1??????????????????????????????1????????????????????????????????????????????????????????????????????1???0?????????0???????0?????00?????????????????????????????????????????????????????????????????0??????????????0????????????0????????????0???????????????????????????1?????????????????????????????????????????????????????????0??????????????????????????????????????????????????????????????????????????????????????0???????????????????????????????????????????????????????????????????????????1?0?0??0?????0????0????0????????0?????????????????????????????????0??????????????????????????????????????????????????0??????????????????????????????0??10????????????0????????????????????????????????????0????????????????????????11???0???1?????????????????????????????0??

Zuolong

1000?00100?1100100001101??000?10?0000???0?10??????????0???0?10?????0????2000??111111?1?0?000????010?0100?1000???????????????????????????????????1???????0?????[01]0[01]?0?0????????????????????????????0?0?0????1??111000?0??2001??0?0??1????0??????????????????2????????????????????0??1??00030??????000?????????????????????????????????????????01????????300000010?0????010?000???00??0?0????101?10?1???02??0011??11?1???01??0?0001????????????0110010?2100000??1001?01???2?????????1?????????0??0?010000101?0?1?0??????????????????????0???0??0?2?0??00??0??1?????0?????????1?0?????????00?0?01??????????0?0??1000?00??0000??0???0??00??00??????0??0?1?0?????100?????00???0??0?????????0??0???????0?0?????1??11??0?0?00????0?????0?????????????????0?0??????00??0???????00011???????00??1??????1?0010??0???????0???00?????000????0???0?00????1??1????1??????00????00???????????1??11??0001????????0??00?1?0????100??????0??00?????????????00?01??0?0??00?????0??0????0??????????????001?00???????????????????0??????????????0???1??0?????00??1?00????00??????0???????00????000????0??0?????0??0?1????1??1????????0???00???0?0?????0??1??1?????????000?????10???????0?????00001??????0??????000????0????????1?????1??1???????????0?0????1???0?0?0??000???1??0????0??00???????0?0??????????????????????????????????00?????00?0????0???00???????????????????0???????0?????000?????????????????????????????????????????????????????????????????????????10???????????????????????????????????????0???????????2????????????????????????????????????????????????????????????00????????????????????????????????????????

;

ccode + 0 5 8 11 13.15 24.26 32.33 35 37 39 42.44 46 56 59.61 64 72 81.83 90 92.93 104 107 120 125 128 133 155 158 160 165 167 174 186 191 193 202 207 215 221 227 231 245 249.250 263 268 274 278 280 283 286 290 292 295 300 313 315 321 323.324 327.330 333.334 337 339 341.342 351 353 355.357 365 368 372 381.382 384 386 394.395 400 404 408 410 413 416 420.421 426 429.430 438 441 453.457 459 462 467.468 475 477 482 496 504 516 542 544 563 569 574 587 593 596 601 606 609 619 626 635 642 650.651 653.654 675 678 684 687 708 732 736 745 752 820 842 849 934 936 939 948 967 990 1020 1049 1054 1110 1142 1168 1182 1205 1215.1216 1251 1354 1393 1406 1432 1446;

proc/;

## References for supplementary material

Agnolín FL, Novas FE (2013) Avian Ancestors: A Review of the Phylogenetic Relationships of the Theropods Unenlagiidae, Microraptoria, Anchiornis and Scansoriopterygidae. Springer

Brett-Surman MK, Paul GS (1985) A new family of bird-like dinosaurs linking Laurasia and Gondwanaland. Journal of Vertebrate Paleontology 5:133–138.

Brusatte SL, Vremir M, Csiki-Sava Z, Turner AH, Watanabe A, Erickson GM, Norell MA (2013) The Osteology of *Balaur bondoc*, an Island-Dwelling Dromaeosaurid (Dinosauria: Theropoda) from the Late Cretaceous of Romania. Bulletin of the American Museum of Natural History 374:1–100.

Chiappe LM (2002) Osteology of the flightless *Patagopteryx* *deferrariisi* from the Late Cretaceous of Patagonia (Argentina). In: Chiappe LM, Witmer LM (eds) Mesozoic Birds: above the heads of dinosaurs. University of California Press, pp 281–361.

Chiappe LM (1993) Enantiornithine (Aves) tarsometatarsi from the Cretaceous Lecho Formation of northwestern Argentina. American Museum Novitates 3083:1–27.

Clarke JA, Norell MA (2002) The morphology and phylogenetic position of *Apsaravis* *ukhaana* from the Late Cretaceous of Mongolia. American Museum Novitates 1–46.

Godefroit P, Cau A, Dong-Yu H, Escuillié F, Wenhao W, Dyke G (2013) A Jurassic avialan dinosaur from China resolves the early phylogenetic history of birds. Nature 498:359–362.

Goloboff PA, Carpenter JM, Arias JS, Esquivel DRM (2008a) Weighting against homoplasy improves phylogenetic analysis of morphological data sets. Cladistics 24:758–773.

Goloboff PA, Farris JS, Nixon KC (2008b) TNT, a free program for phylogenetic analysis. Cladistics 24:774–786.

Lee MSY, Cau A, Naish D, Dyke GJ (2014) Sustained miniaturization and anatomical innovation in the dinosaurian ancestors of birds. Science 345(6196): 562–566.

Longrich NR, Currie PJ (2009) *Albertonykus borealis*, a new alvarezsaur (Dinosauria: Theropoda) from the Early Maastrichtian of Alberta, Canada: implications for the systematics and ecology of the Alvarezsauridae. Cretaceous Research 30:239–252.

Norell MA, Makovicky PJ (1997) Important Features of the Dromaeosaurid Skeleton Information from a new specimen. American Museum Novitates 3215:1–28.

Norell MA, Makovicky PJ (1999) Important Features of the Dromaeosaurid Skeleton II: Information from Newly Collected Specimens of *Velociraptor* *mongoliensis*. American Museum Novitates 3282:1–45.

O’Connor JK, Averianov AO, Zelenkov NV (2014) A confuciusornithiform (Aves, Pygostylia)-like tarsometatarsus from the Early Cretaceous of Siberia and a discussion of the evolution of avian hind limb musculature. Journal of Vertebrate Paleontology 34:647–656.

Senter P (2007) A method for distinguishing dromaeosaurid manual unguals from pedal “sickle claws”. Bulletin of Gunma Museum of Natural History 11:1–6.

Swofford DL (2002) PAUP*. Phylogenetic Analysis Using Parsimony (*and Other Methods). Version 4. Sinauer Associates, Sunderland, Massachusetts.

Turner AH, Makovicky PJ, Norell MA (2012) A Review of Dromaeosaurid Systematics and Paravian Phylogeny. Bulletin of the American Museum of Natural History 371:1–206.

Walker C, Dyke G (2009) Euenantiornithine birds from the Late Cretaceous of El Brete (Argentina). Irish Journal of Earth Sciences 27:15–62.

Wagner GP, Gauthier J (1999) 1,2,3 = 2,3,4: A solution to the problem of the homology of the digits in the avian hand. Proceedings of the National Academy of Science USA. 96:5111–5116.

Xu X, Choinere J, Pittman M, Tan Q, Xiao D, Li Z, Tan L, Clark J, Norell MA, Hone DWE, Sullivan C. 2010. A new dromaeosaurid (Dinosauria: Theropoda) from the Upper Cretaceous Wulansuhai Formation of Inner Mongolia, China. Zootaxa 2403: 1–9.

Xu X, Pittman M, Sullivan C, Choiniere JN, Tan Q-W, Clark JM, Norell MA, Wang S (2015) The taxonomic status of the Late Cretaceous dromaeosaurid *Linheraptor exquisitus* and its implications for dromaeosaurid systematics. Vertebrata PalAsiatica, 53: 29-62.

Zhang Z, Chiappe LM, Han G, Chinsamy A (2013) A large bird from the Early Cretaceous of China: new information on the skull of enantiornithines. Journal of Vertebrate Paleontology 33:1176–1189.

Zhou Z, Zhang F (2003) Anatomy of the primitive bird *Sapeornis* *chaoyangensis* from the Early Cretaceous of Liaoning, China. Canadian Journal of Earth Sciences 40:731–747.
